# Proceedings of the Frontiers in Retrovirology Conference 2018

**DOI:** 10.1186/s12977-018-0436-z

**Published:** 2018-09-07

## Oral presentations

### Session 1: Entry and uncoating

#### O1 Visualization of the productive uncoating of single HIV-1 in living cells

##### Ashwanth C. Francis^1^, Gregory B. Melikyan^1,2^

###### ^1^Division of Paediatric Infectious Diseases, Emory University School of Medicine, Atlanta, Georgia, USA; ^2^Children’s Healthcare of Atlanta, Atlanta, Georgia, USA

**Correspondence:** Ashwanth C. Francis

*Retrovirology* 2018, **15(Suppl 1)**:O1

HIV-1 uncoating, which involves a partial or complete loss of capsid proteins (CA), is one of the most enigmatic steps in virus entry. By engineering a tetrameric CyclophilinA-DsRed (CypA-DsRed) fusion protein we generated a non-invasive label for the viral CA to report single HIV-1 uncoating [1]. Here, to identify productive uncoating events that lead to the virus nuclear import and infection, we performed extended time-lapse imaging and analysis of eGFP-encoding HIV-1 pseudoviruses co-labeled with INsfGFP and CypA-DsRed from 0 to 24 h post-infection. Single particle analysis of virus uncoating and nuclear import revealed that HIV-1 nuclear entry proceeds through steps of virus docking at the nuclear envelope (NE), followed by an accelerated loss of CypA-DsRed and nuclear penetration of INsfGFP complexes. The loss of CypA-DsRed at the NE reflected virus uncoating, since similar reduction in the CypA-DsRed fluorescence and in the CA signal of INsfGFP complex, as determined by immuno-fluorescence, is observed upon nuclear import. In agreement with the previous fixed cell studies, a subset of CypA-DsRed can remain associated with nuclear IN complexes and these complexes can be tracked for several hours, suggesting that HIV-1 undergoes terminal uncoating at the NE. Interestingly, however, a fraction of nuclear IN complexes disappears at varied times post-nuclear entry, and this loss of IN signal strongly correlates with subsequent expression of the eGFP reporter of infection. The N74D CA mutant, which uses alternative nuclear entry pathways, also uncoats at the NE, but fails to sufficiently penetrate into the nucleus and exhibits peripheral disappearance of IN complexes prior to eGFP expression. The > 3-fold slower kinetics of CypA-DsRed loss after the N74D mutant docking at the NE compared to wild-type viruses suggests the involvement of host factors at the NE in the accelerated uncoating and nuclear penetration of HIV-1. Collectively, our data demonstrate that CA-dependent steps of docking and uncoating at the NE are pre-requisites for HIV-1 nuclear import and infection. This work was supported by the NIH R01 grant AI129862 to G.B.M.

**Keywords:** Live cell microscopy; Capsid; Uncoating; Nuclear Import

**Reference**

1. Francis AC., Marin M., Shi J., Aiken C., Melikyan GB. Time-Resolved Imaging of Single HIV-1 Uncoating *In Vitro* and in Living Cells. PLoS Pathog. 2016; Jun 20; 12(6):e1005709.

#### O2 Mimicry of a +TIP binding motif by HIV-1 capsid coordinates early steps of infection

##### Eveline Santos da Silva, Michael K. Delaney, Mojgan H. Naghavi

###### Department of Microbiology-Immunology, Northwestern University Feinberg School of Medicine, Chicago, IL, USA

**Correspondence:** Mojgan H. Naghavi

*Retrovirology* 2018, **15(Suppl 1)**:O2

Upon entry, HIV-1 exploits microtubule (MT) filaments for transport to the nucleus. Within the host cell, dynamic MTs continuously grow and shrink to explore the intracellular environment through a process of “search and capture”. Their dynamic behavior is controlled by a small and highly specialized family of proteins known as plus-end tracking proteins (+TIPs). Although many viruses are known to exploit MTs for infection, how +TIPs might contribute to this process was unclear until recently. Our work provided the first direct evidence that a virus, HIV-1 actively stabilizes MTs by targeting two distinct +TIPs to control both its trafficking and uncoating. We found that soon after entry, the HIV-1 matrix protein binds the +TIP Kif4 to rapidly induce MT stabilization. This initial induction is further enhanced by incoming capsid (CA) targeting a second +TIP complex consisting of the formins, Diaphanous 1 and 2, offering a hand-off strategy for amplification of the levels of stable MTs as the virus proceeds through early infection.

Here, we tested the ability of other +TIPs such as cytoplasmic linker protein-170 (CLIP-170), known to promote MT growth as well as linkage to intracellular cargoes, and its partner dynactin (DCTN1) in influencing early HIV-1 infection. We found that while both CLIP-170 and DCTN1 bind in vitro assembled HIV-1 CA-NC complexes, these factors exert opposing effects on CA stability as well as early infection in multiple cell types including natural target cells, suggesting a potential competition between these factors for association with incoming capsids. Indeed, validating this competition, we found more CLIP-170 bound to CA-NC complexes in DCTN1 depleted cells while addition of DCTN1 reduced the amount of CLIP-170 on these complexes. In an attempt to understand why various +TIPs associate with HIV-1 capsid, domain analysis revealed the unexpected discovery of a common +TIP binding motif within HIV-1 capsid. Fusion of the housekeeping protein GAPDH to this +TIP binding homology sequence conferred on GAPDH the ability to interact with CLIP-170 or DCTN1. Collectively, our findings highlight how +TIP binding motif mimicry within HIV capsid creates functional modules for various +TIPs found at the ends of growing MTs, enabling the virus to induce global MT stabilization and coordinating different steps of early infection.

**Keywords:** HIV-1; Capsid; Uncoating; Trafficking

#### O3 Single virus imaging of HIV-1 entry with fluorescently labeled capsid

##### Irena Zurnic^1^, Lieve Dirix^1,2^, Veerle Lemmens^1,2,^ Susana Rocha^2^, Frauke Christ^1^, Johan Hofkens^2^, Jelle Hendrix^2,3^, Zeger Debyser^1^

###### ^1^Laboratory of Molecular Virology and Gene Therapy, Department of Pharmacological and Pharmaceutical Sciences, KU Leuven, Leuven, Belgium; ^2^Molecular Imaging and Photonics, Department of Chemistry, KU Leuven, Heverlee, Belgium; ^3^Faculty of Medicine and Life Sciences and Biomedical Research Institute, Hasselt University, Hasselt, Belgium

**Correspondence:** Irena Zurnic

*Retrovirology* 2018, **15(Suppl 1)**:O3

At present, a consensus HIV-1 uncoating model is lacking, mostly due to conflicting results on intracellular capsid distribution, which can benefit from a robust method of imaging functional viruses containing labeled CA.

We fluorescently labeled CA within an NL4.3-based molecular clone and evaluated replication of these labeled viruses. We generated dually labeled VSV-G pseudotyped particles containing C-terminally eGFP-tagged CA (CA-eGFP) and Vpr-transincorporated integrase with a C-terminal mCherry fusion (IN-mCherry). Since the construct encoding CA-eGFP by itself did not allow viral particle release, we mixed it with a plasmid encoding wild type, unlabeled (WT) CA at a 1:10 ratio during virus production. These mixed CA particles restored infectivity in single-round infection experiments to the level of particles containing only IN-mCherry, used in parallel as a reference.

We evaluated the potential to study HIV-1 entry at a single virus level by confocal microscopy. We investigated the cellular distribution and intensity of fluorescent, particle-derived CA and IN in fixed HeLa P4 cells at 6 h post infection. At this point, colocalisation of fluorescent CA and IN was observed in 20–30% of all cytosolic complexes. CA-eGFP containing complexes accumulated in the perinuclear area, but only 10–15% of these complexes also contained IN-mCherry. Using both CA-eGFP and immunocytochemistry, we confirmed the presence of CA in the nucleus, which rarely colocalized with IN-mCherry. Under PF74 treatment, the number of nuclear complexes containing labeled IN decreased 15-fold and those with labeled CA decreased 5-fold, suggesting a nuclear import block. Of note, due to the low level incorporation of CA-eGFP, it was impossible to reliably measure the fine changes in intracellular CA-eGFP complex intensity. However, since the CA-eGFP signal is persistent for up to 6 h post infection, this labelling allows us to characterize its nuclear import. Importantly, the intracellular distribution and fluorescence intensity of IN-mCherry complexes were unaffected by CA-eGFP labeling. The inhibition of CA-eGFP labeled viruses with PF74 suggests that at least some of the dually labeled particles undergo bona fide uncoating and nuclear import.

Directly labeled CA allows single virus imaging of HIV-1 pre-integration steps and represents a suitable system to track HIV-1 particles, the viral PIC and the fate of the particle-derived capsid.

**Keywords:** Retrovirology; Capsid; Label; HIV-1 entry; Confocal Microscopy

#### O4 Ultrastructural conformation states of HIV-1 during the early steps of viral infection

##### Guillermo Blanco-Rodriguez^1^, Anastasia Gazi^2^, Jacomine Krijnse-Locker^2^, Pierre Charneau^1^, Francesca Di Nunzio^1^

###### ^1^Department of Virology, Pasteur Institute, Paris, France; ^2^Ultrapole, Pasteur Institute, Paris, France

**Correspondence:** Guillermo Blanco-Rodriguez

*Retrovirology* 2018, **15(Suppl 1)**:O4

When HIV-1 enters in the cells, the capsid (CA) core containing the viral genetic material is released in the cytoplasm. The core is composed by ~ 1500 capsid monomers organized in hexamers or pentamers to give rise a conical shape of 120 × 60 × 40 nm. This structure acts as a shield against cellular antiviral sensors and maintains an adequate environment for the reverse transcription. However, the capsid is not a passive but it is a dynamic structure that interacts with several cellular factors required for a successful infection. Therefore, the lack of appropriate tools to study the dynamics of viral core rearrangements during HIV-1 cytoplasmic journey towards the nuclear pore complexes (NPCs) generates a controversy in the field.

In this study we applied immunofluorescence assay coupled to immunoelectron microscopy to investigate the state of viral replication complexes at the NPC. In particular, we used a VSV-G pseudotyped or Env wild type HIV-1 containing a HA tag fused to the integrase (IN). Importantly, these viruses infect target cells similarly to the unmodified HIV-1.

We observed that the number of viral complexes containing both CA and IN are reduced during the time of infection. Interestingly, we were able to detect intermediate states of viral replication complexes. We observed assembled cores at the NPC that we identified by their morphology and by direct gold labeling against CA. We also observed viral complexes translocating the NPC and entering into the nucleus. Interestingly, the assembly CA state can be modulated with the use of drugs. For example when we treated our cells with PF74 (a drug that strongly interacts with the hydrophobic pocket formed between the NTD-CTD domains of CA hexamers, the same region bound by cellular factors like CPSF6 and Nup153) we observed a dual effect dose-dependent. HIV-1 infected cells in presence of low doses of PF74 show an increase of CA-IN colocalizations in the cytoplasm with respect to the cells infected without drug, suggesting a core stabilization effect. When high doses of PF74 have been used, the effect was the opposite than the one observed at low doses, less colocalizations CA-IN are detected, suggesting a loss of intact cores.

Overall we highlighted different states of viral cores during early steps of infection. We also observed that the CA state can be modulated using PF74 in a dose dependent manner, inhibiting the interaction with key nuclear factors or inducing a premature uncoating.

**Keywords:** Retrovirology; Conference; Leuven; Belgium

### Session 2: RT and integration

#### O5 Sensing of chromatin structures by the carboxyterminal domain of retroviral integrases

##### D. Lapaillerie^1,7^, E. Mauro^1,7^, C. Miskey^2^, Z. Ivics^2^, C. Thambo-Roorick^3^, J. Toutain^3^, O. Delelis^4^, P. Gouet^5^, M. Ruff^6^, P. Lesbats^1,7^, V. Parissi^1,7^

###### ^1^Fundamental Microbiology and Pathogenicity Laboratory, CNRS-University of Bordeaux, Bordeaux, France; ^2^Division of Medical Biotechnology Paul Ehrlich Institute. Langen, Germany; ^3^Hôpital Pellegrin, University of Bordeaux, Bordeaux, France; ^4^CNRS, Cachan, France; ^5^MMSB-Institute of the Biology and Chemistry of Proteins, Lyon 1 University, Lyon, France; ^6^IGBMC, Département de Biologie Structurale Intégrative, CNRS, Strasbourg, France; ^7^International Associated Laboratory (LIA) of Microbiology and Immunology, CNRS/University de Bordeaux/Heinrich Pette Institute-Leibniz Institute for Experimental Virology, Viral DNA integration and chromatin dynamics Network (DyNAVir)

**Correspondence:** V. Parissi

*Retrovirology* 2018, **15(Suppl 1)**:O5

Retroviral integration, catalyzed by integrase (IN), is not random into the host and the search for suitable chromatin loci is a complex multifactorial mechanism. The final association between the incoming IN/viral DNA complexes (intasomes) and the nucleosomal target DNA is a key step in this process. Previous works from our lab and others indicate that retroviral INs can directly bind histone components of the nucleosome via their carboxy-terminal domain (CTD) [1, 2]. Furthermore, in other retroviruses–related integrases, the CTD carries a chromodomain that directly binds chromatin [3]. We, thus, wondered whether the retroviral INs CTD might have a chromatin binder function that could participate in the viral DNA insertion into host chromatin.

Using a structure–function approach we have shown that the different retroviral INs do not interact similarly with the nucleosome in vitro and mutations of the binding site in the HIV-1 IN chromatin binding property using a chromosome spreads model led us to demonstrate that IN possesses an intrinsic property to recognize specific chromatin regions. Further analyses showed that the IN CTD is responsible for in this chromatin binding function and the presence of histone tails is required in the process. Additionally, LEDGF/p75 IN cofactor was shown to modulate the recognition of these chromatin regions by HIV-1 IN. Introduction of IN CTD mutations in lentiviral vectors led to a partial retargeting of their cellular integration sites toward distinct chromatin regions.

Taking together, our data indicate that HIV-1 IN CTD can sense chromatin structures and participates in the selection of the optimal integration sites within the host chromosomes. Modulation of this new function by previously identified host factors associated with chromatin and transcription apparatus, as the histone chaperon FACT [4], will also be discussed.

Our work highlights and new function in retroviral INs that could constitute an attractive target for future potential therapeutic applications and a new tool for controlling viral vectors insertion sites in gene transfer and gene therapy approaches.

**Keywords:** Integration; Chromatin; Transcription; HIV-1

**References**

1. Maskell D. P. et al. Structural basis for retroviral integration into nucleosomes. *Nature* (2015).
2. Benleulmi M. S. et al. Modulation of the functional association between the HIV-1 intasome and the nucleosome by histone amino-terminal tails. *Retrovirology* **14,** 54 (2017).
3. Llorens C., Fares M. A., Moya A. Relationships of gag-pol diversity between Ty3/Gypsy and Retroviridae LTR retroelements and the three kings hypothesis. *BMC Evol Biol* **8,** 276 (2008).
4. Matysiak J. et al. Modulation of chromatin structure by the FACT histone chaperone complex regulates HIV-1 integration. *Retrovirology* **14,** (2017).

#### O6 Cryo-EM structures of lentiviral intasomes

##### Allison Ballandras-Colas, Nicola Cook, Valerie E. Pye, Peter Cherepanov

###### Chromatin Structure and Mobile DNA Laboratory, The Francis Crick Institute, London, UK

**Correspondence:** Peter Cherepanov

*Retrovirology* 2018, **15(Suppl 1)**:O6

Retroviral integration is mediated by the intasome, a nucleoprotein complex comprising a multimer of integrase (IN) assembled on viral DNA ends. Unfavourable biochemical properties of HIV-1 IN required the use of hyperactive and solubilizing mutations, which by their nature dramatically change properties of the protein. By contrast, IN from the Maedi-visna virus (MVV), an ovine lentivirus, is soluble and highly active in vitro in the presence of the common lentiviral host factor LEDGF. We have now determined the structure of the MVV intasome-LEDGF complex at 4.4 Å resolution using single-particle cryo-EM. The intasome comprises a homo-hexadecamer of IN with a tetramer-of-tetramers architecture. The conserved intasomal core (CIC), previously observed in non-lentiviral systems, is formed between two IN tetramers, with a pair of C-terminal domains (CTDs) from flanking tetramers completing the synaptic interface. The structure revealed two preferential LEDGF binding sites on the CIC, suggesting an evolutionarily conserved mode of binding for the chromatin targeting factors by the retroviral intasomes. The hexadecameric architecture of the intasome is necessitated by the alpha-helical nature of the linker connecting the catalytic core domains (CCDs) and CTDs in lentiviral INs and the propensity of these proteins to form tetramers in solution. Although these features are conserved in HIV-1 IN, the relatively low level of amino acid sequence conservation (< 30%) limits the use of MVV as the model for the development of HIV-1 IN inhibitors. To develop a model suitable for the studies of HIV-1 IN strand transfer inhibitors and the mechanism of viral resistance to these small molecules, we characterized IN proteins from diverse simian immunodeficiency viruses (SIVs). IN from one SIV isolate, which displays 75% amino acid sequence identity with HIV-1 IN, is highly competent at concerted integration and readily forms active nucleoprotein complexes with viral DNA in vitro. Electron microscopy revealed that the SIV IN-DNA complexes assembled in vitro represent linear polymers with the repeating unit harbouring a full CIC. We show that the SIV intasomes are amenable to high-resolution structural characterization and therefore represent an attractive model to study HIV–1 IN strand transfer inhibitors.

**Keywords:** Cryo-EM; Intasome; MVV; SIV

#### O7 Nuclear Localization of Transcriptionally Active Proviruses

##### Ryan C. Burdick^1^, Claire Deleage^2^, Jacob D. Estes^2,3^, Wei-Shau Hu^4^, Vinay K. Pathak^1^

###### ^1^Viral Mutation Section, HIV DRP, NCI, Frederick, MD, USA; ^2^AIDS and Cancer Virus Program, Leidos Biomedical Research, Inc., Frederick, MD, USA; ^3^Vaccine and Gene Therapy Institute and Oregon National Primate Research Center, Oregon Health & Science University, Beaverton, OR, USA; ^4^Viral Recombination Section, HIV DRP, NCI, Frederick, MD, USA

**Correspondence:** Ryan C. Burdick

*Retrovirology* 2018, **15(Suppl 1)**:O7

HIV-1 nuclear import and integration into the host DNA are critical steps in viral replication. We recently captured the nuclear import of APOBEC3F-YFP- and IN-YFP-labeled viral complexes using live-cell microscopy and showed that these viral complexes moved away from the nuclear point of entry but remained near the nuclear periphery. A comparison of the movement of viral complexes to those of proviral transcription sites suggests that HIV-1 complexes quickly tether to chromatin at or near their sites of integration. Several groups have also shown that viral DNA is primarily located near the nuclear periphery; however, a recent report concluded that viral DNA integration occurs in genes located throughout the nucleus, and that disruption of the CA-CPSF6 interaction results in the peripheral distribution of viral DNA. Previous studies did not distinguish between integrated and unintegrated viral DNA, and our studies using DNA-fluorescence in situ hybridization showed that both can be detected in the nuclei of infected cells 24 h after infection. To compare the peripheral location of nuclear viral complexes to their sites of integration, we developed an RNA-fluorescence in situ hybridization assay to detect transcriptionally active proviruses. Combinations of low multiplicity of infection, integrase inhibitor raltegravir, and reverse transcriptase inhibitor nevirapine were used to show that nascent HIV-1 RNA from transcriptionally active integrated proviruses was detected. In addition, the HIV-1 RNA transcription site can be detected in the nuclei of infected cells and can be used as a surrogate for integrated proviral DNA. The integrated proviruses were located near the nuclear periphery and their nuclear penetration distance was not different than that of A3F-YFP or IN-YFP labeled viral complexes but was different than a random simulation. The N74D of A77V CA mutant viruses which do not bind CPSF6 also integrated near the nuclear periphery, and their nuclear penetration distance was not different from WT proviruses, indicating that CPSF6-binding defective mutants integrate at peripheral locations that are indistinguishable from the wild-type viruses. In conclusion, we developed a new assay to detect transcriptionally active proviruses and show that viral DNA integrates at sites near the nuclear periphery in newly infected cells. In addition, these results provide new insights into the three-dimensional organization of the human genome.

**Keywords:** Integration; Fluorescence in situ hybridization; Capsid; Transcription

#### O8 Recombination Is Required for Efficient HIV-1 Replication

##### Jonathan M. Rawson^1#^, Olga A. Nikolaitchik^1#^, Brandon F. Keele^2^, Vinay K. Pathak^3^, Wei-Shau Hu^1^

###### ^1^Viral Recombination Section and ^3^Viral Mutation Section, HIV Dynamics and Replication Program, NCI, Frederick, MD 21702, USA; ^2^AIDS and Cancer Virus Program, Leidos Biomedical Research, Inc., FNLCR, Frederick, MD 21702*, USA*

**Correspondence:** Wei-Shau Hu

*Retrovirology* 2018, **15(Suppl 1)**:O8

^#^Equal contribution

HIV-1 packages two genomic RNA copies into every virus particle, which allows reverse transcriptase (RT) to frequently switch between the genomes during viral DNA synthesis, resulting in recombination. Recombination can generate viral variants that escape from the host immune response or resist antiviral treatments, but it is not yet clear whether recombination is strictly required for viral replication. Currently, there are two prevailing hypotheses: the forced copy-choice model proposes that RNA breaks force RT to switch templates to complete reverse transcription, whereas the dynamic copy-choice model posits that template switching is not necessarily forced and is controlled by the relative balance of RT’s polymerase and RNase H activities. To test these two models, we engineered near full-length HIV-1 constructs in which recombination was blocked in defined regions of the genome by reducing sequence homology. If RT must switch between broken RNAs to complete reverse transcription, blocking recombination will significantly reduce viral infectivity. In contrast, if not all recombination is forced, some viruses may still be able to maintain infectivity. Each of these engineered constructs contained a marker gene and a defective green fluorescent protein (*gfp*) gene. Recombination can restore a functional *gfp* gene; hence, *gfp* reconstitution was used as a surrogate indicator for recombination. We found that blocking recombination in part of the viral genome significantly reduced *gfp* reconstitution rates without affecting overall viral titers. Further, single-genome sequencing (SGS) analyses of recombinant proviruses revealed that blocking recombination led to large deletions exhibiting hallmarks of non-homologous template switching. Collectively, these data indicate that HIV-1 requires recombination to successfully replicate and maintain genome integrity. However, the observed decreases in *gfp* recombination frequencies suggest that not all recombination events are forced, implying that recombination occurs through both forced and dynamic copy-choice mechanisms.

This work was supported in part with federal funds from the NCI; NIH under contract HHSN261200800001E.

**Keywords:** HIV-1; Recombination; Reverse transcription; Replication

### Session 3: Transcription and assembly

#### O9 Determinants of HIV-1 genomic RNA specific encapsidation by the Pr55^Gag^ precursor

##### Noé Dubois^1^, Redmond Smyth^1^, Bill McKinstry^2^, Johnson Mak^2,3^, Jean-Christophe Paillart^1^, Roland Marquet^1^, Serena Bernacchi^1^

###### ^1^Architecture et Réactivité de l’ARN, Université de Strasbourg, Strasbourg, France; ^2^Commonwealth Scientific and Industrial Research Organization, Livestock Industries, Australian Animal Health Laboratory, Geelong, Victoria, Australia; ^3^Institute for Glycomics, Griffith University, Melbourne, Victoria, Australia

**Correspondence:** Serena Bernacchi

*Retrovirology* 2018, **15(Suppl 1)**:O9

The HIV-1 Pr55^Gag^ precursor specifically selects the genomic RNA (gRNA) from a large variety of cellular and spliced viral RNAs (svRNAs) and drives the virus assembly at the plasma membrane. To gain a better understanding of the selection process, we analyzed by fluorescence spectroscopy the interactions between Pr55^Gag^ and a large panel of viral RNA fragments encompassing the main packaging signal (Psi) and its flanking regions. We showed that the gRNA harbors a high affinity binding site that is absent from svRNA species, suggesting that this peculiarity might be crucial for the selection of the HIV-1 genome. We observed that few copies of Pr55^Gag^ specifically associate with the 5’ region of the gRNA, and that the internal loop of stem-loop 1 (SL1) in Psi is crucial for the specificity of the interaction. Furthermore, our analysis supports the existence of a long-range tertiary interaction involving sequences upstream and downstream of the Psi region that would thus promote the optimal exposure of SL1 for efficient Pr55^Gag^ recognition. Altogether, our results shed light on the molecular mechanisms allowing the specific selection of gRNA by Pr55^Gag^ amongst a variety of svRNAs, all harboring SL1 in their first common exon [1,2].

During the viral assembly the C-terminal p6 domain of Pr55^Gag^ has been shown to be sequestered by cellular and viral factors, this way promoting viral particle release. Here we tested whether the p6 domain also contributes to the RNA binding specificity to favour gRNA encapsidation. To this aim we compared systematically Pr55^Gag^ and Gagp6 binding to a panel of viral and cellular RNAs. Gagp6 is a truncated form of Pr55^Gag^ lacking the p6 domain usually used as a default surrogate for wild type Pr55^Gag^ for in vitro analysis. We observed that p6 deletion resulted in a similar affinity for all tested RNAs and none of the major signals, regulating the specific binding of Pr55^Gag^ to gRNA, impacts on Gagp6 binding. We propose that in the context of full-length Pr55^Gag^ p6 interaction with the NC domain regulates the gRNA binding specificity. In sum these results demonstrate a novel role of the p6 domain in the specificity of Pr55^Gag^-RNA interactions, and strongly suggest that the p6 domain contributes to the discrimination of HIV-1 gRNA from cellular and svRNAs, which is necessary for its selective encapsidation.

**Keywords:** HIV-1 Gag precursor; Specific gRNA selection; Encapsidation; Protein-RNA binding affinity.

**References**

1. Abd El-Wahab E.W., Smyth R.P., Mailler E., Bernacchi S., Vivet-Boudou V., Hijnen M., Jossinet F., Mak J., Paillart J.-C., Marquet R. *Nat Commun*, 5 **2014**,4304.
2. Bernacchi S., Abd El-Wahab E.W., Dubois N., Hijnen M., Smyth R.P., Mak J., Marquet R., Paillart J.-C., *RNA Biol,* 14 2017, 90–103.

#### O10 ESCRT-II functions by linking to ESCRT-I in HIV budding

##### Bo Meng^1^, Julia Kenyon^1,2^, Andrew Lever^1,2^

###### ^1^Division of Infectious Disease, Department of Medicine, University of Cambridge, Cambridge, UK; ^2^Yong Loo Lin School of Medicine, National University of Singapore, Singapore

**Correspondence:** Bo Meng

*Retrovirology* 2018, **15(Suppl 1)**:O10

HIV uses the ESCRT protein pathway to bud from infected cells [1]. The roles of ESCRT-I and -III in HIV budding are firmly established, however the participation of ESCRT-II in this process has been controversial [2]. Previously we utilised a CRISPR-Cas9 generated EAP45 knockout cell line to investigate this and to eliminate any residual ESCRT-II that might confound the knockdown approach [3]. Using this cell line we showed unequivocally for the first time that ESCRT-II is important for efficient HIV budding and that ESCRT-II is also apparently involved in other viral processes. Here, extending from our previous observations we have studied the effects of EAP45 on post integration events in the HIV life cycle.

Overexpression of EAP45 in EAP45 knockout cells led to rescue of viral release. Using specific mutations in EAP45 allowed us to ascertain which domains were responsible for rescue of budding. We showed that at steady state this rescue is only observed in the presence of Gag and Gag/Pol but not for a sole Gag expressor. This suggests that the size of cargo determines the usage of ESCRT-II and that ESCRT-II plays a role at an early budding stage. Finally, we observed that EAP45 may be functioning through the YPXL-ALIX pathway as partial rescue is seen in PTAP but not YPXL mutated viruses. Our study clarifies the role of ESCRT-II in HIV budding and reinforces that ESCRT-II plays a crucial role in this process.

**Keywords:** HIV; Budding; ESCRT; ALIX

**References**

1. Sundquist, W.I., Kräusslich, H.G. HIV-1 assembly, budding, and maturation. *Cold Spring Harbor Perspectives in Medicine*. **2012**; 2:1–24.
2. Langelier, C., von Schwedler, U.K., Fisher, R.D., De Domenico, I., White, P.L., Hill, C.P., Kaplan, J., Ward, D., Sundquist, W.I. Human ESCRT-II complex and its role in human immunodeficiency virus type 1 release. *Journal of Virology*. **2006**; 80:9465–9480.
3. Meng, B., Ip N.C., Prestwood L.J., Abbink T.E, Lever A.M. Evidence that the endosomal sorting complex required for transport-II (ESCRT-II) is required for efficient human Immunodeficiency virus-1 (HIV-1) production. *Retrovirology*. **2015**; 12:72.

#### O11 The 5’ polyadenylation signal regulates HIV-1 genomic RNA production and packaging

##### Redmond P. Smyth^1^, Maureen R. Smith^2^, Anne-Caroline Jousset^1^, Laurence Despons^1^, Géraldine Laumond^3^, Thomas Decoville^3^, Pierre Cattenoz^1^, Christiane Moog^3^, Fabrice Jossinet^1^, Marylène Mougel^4^, Jean-Christophe Paillart^1^, Max von Kleist^2^, Roland Marquet^1^

###### ^1^Université de Strasbourg, Strasbourg, France; ^2^Department of Mathematics and Computer Science, Freie Universität Berlin, Berlin, Germany; ^3^Fédération de Médecine Translationnelle de Strasbourg, Université de Strasbourg, Strasbourg, France; ^4^Université de Montpellier, Montpellier, France

**Correspondence:** Roland Marquet

*Retrovirology* 2018, **15(Suppl 1)**:O11

Non-coding RNA regulatory elements are important for viral replication, making them promising targets for therapeutic intervention. However, regulatory RNA is challenging to detect and characterise using classical structure–function assays. Here, we present in cell Mutational Interference Mapping Experiment (in cell MIME) as a way to define RNA regulatory landscapes at single nucleotide resolution under native conditions. In cell MIME is based on (i) random mutation of an RNA target, (ii) expression of mutated RNA in cells, (iii) physical separation of RNA into functional and non-functional populations, and (iv) high-throughput sequencing to identify mutations affecting function. We used in cell MIME to define RNA elements within the 5’ region of the HIV-1 genomic RNA (gRNA) that are important for viral replication in cells [1]. We identified three distinct RNA motifs controlling intracellular gRNA production and two distinct motifs required for gRNA packaging into virions. Our analysis reveals the ^73^AAUAAA^78^ polyadenylation signal within the 5’PolyA domain as a dual regulator of gRNA production and gRNA packaging, and demonstrates that a functional polyadenylation signal is required for viral packaging even though it negatively affects gRNA production. Our data indicate that even though premature cleavage and polyadenylation of gRNA is strongly repressed by binding of U1 snRNA to the major slice donor site, repression is not complete and single mutations in the polyadenylation signal strongly increase the gRNA steady-state levels. Unexpectedly, the polyadenylation signal appears as a positive packaging signal even though comparison of in vitro [2] and in cell MIME suggests that it does not directly recognize the Pr55Gag precursor which is the major player of gRNA packaging and viral assembly.

**Keywords:** HIV; Genomic RNA; RNA processing; RNA packaging

**References**

1. Smyth, R.P., Smith, M.R., Jousset, A.-C., Despons, L., Laumond, G., Decoville, T., Cattenoz, P., Moog, C., Jossinet, F., Mougel, M., Paillart, J.-C., von Kleist, M., Marquet, R., *Nucleic Acids Res.*, **2018** 10.1093/nar/gky152, ahead of print.
2. Smyth, R.P., Despons, L., Huili, G., Bernacchi, S., Hijnen, M., Mak, J., Jossinet, F., Weixi, L., Paillart, J.-C., von Kleist, M., Marquet, R., *Nat. Methods*, 12(9) **2015**, 866–72.

#### O12 Determinants of HIV-1 nuclear import kinetics

##### Adarsh Dharan, Amani Eddins, Edward M Campbell

###### Department of Microbiology and Immunology, Loyola University Chicago, Maywood, IL, USA

**Correspondence:** Edward M Campbell

*Retrovirology* 2018, **15(Suppl 1)**:O12

The process of HIV-1 nuclear import and integration, whereby the viral genome translocates through the nuclear pore complex and successfully integrates into the host genome, has remained one of the least well understood process in the HIV-1 life cycle. However, numerous studies suggest that the spatiotemporal regulation of this process is critical in allowing the virus to avoid detection by cytoplasmic sensors, which would otherwise trigger the induction of antiviral genes capable of inhibiting viral infection or replication. Therefore, understanding the kinetics by which the viral genome traverses the nuclear pore complex (NPC), and the viral and cellular determinants influencing this process, may allow the development of strategies designed to perturb this process and thereby stimulating host cell responses capable of preventing viral replication. However, despite being the subject of numerous studies, there are currently no methods that allow the kinetics of nuclear import to be directly monitored. Currently, the formation of two long terminal repeat (2-LTR) circles is the most commonly used assay to measure HIV-1 nuclear import. However, given the indirect nature of this assay, 2-LTR circles assay does not provide reliable information regarding nuclear import kinetics and may not reflect the nuclear import of viral genomes which will go on to productively integrate into the host genome. To circumvent this, we have developed an assay that allows us to monitor the nuclear import kinetics (NIK) of the HIV-1 genome. This assay blocks active transport through the NPC by inducing the drug induced dimerization of Nup62, a central pore protein of the NPC. Utilizing the NIK assay, we have now the ability to measure HIV-1 nuclear import kinetics in HeLa cells and THP-1 cells differentiated into macrophages and currently we are extending this assay to understand HIV-1 nuclear import in primary T cell and human microglia cells. Using this assay, we can determine how specific cellular and viral factors influence the kinetics of HIV-1 nuclear import.

**Keywords:** Retrovirology; HIV-1; Nuclear import; Capsid; CypA; Nucleoporin; CPSF6

#### O13 In depth HIV-1 transcriptome characterization using Oxford Nanopore MinION sequencing

##### Nam Nguyen Quang^1^, Sophie Goudey^1^, Ammara Mohammad^2,^ Sophie Lemoine^2^, Emmanuel Ségéral^1^, Jean-Christophe Paillart^3^, Clarisse Berlioz-Torrent^1^, Stéphane Emiliani^1^, Sarah Gallois-Montbrun^1^

###### ^1^Institut Cochin, Université Paris Descartes, Paris, France; ^2^Institut de Biologie de l’Ecole Normale Supérieure, Paris, France; ^3^Architecture et Réactivité de l’ARN, Université de Strasbourg, Strasbourg, France

**Correspondence:** Sarah Gallois-Montbrun

*Retrovirology* 2018, **15(Suppl 1)**:O13

HIV-1 splicing is a crucial step of the virus replication as it impacts both the production of the viral genome and the viral proteins expression. Through the use of 4 major splice donor and 7 acceptors sites, this cotranscriptional process allows the production of more than a hundred different transcripts from a single 9-kb pre-mRNA. HIV-1 transcripts are classified into three major classes of viral RNAs: 9-kb Unspliced (US), 4-kb singly spliced (SS) and 2-kb multiply spliced (MS) RNAs. As imbalance of the different viral RNAs species can have dramatic effects for viral production, this process is thightly regulated.

Short read deep sequencing techniques have enabled the identification of new splice junctions and gained new informations on the nature of HIV transcripts and their abundance. Pacific Biosciences (PacBio) sequencing technology, by allowing the sequencing of HIV-1 RNA stretches up to 2629 bp, even allowed the identification of new HIV-1 isoforms.

In this study, we used the Oxford Nanopore Technologies (ONT) MinION, another long read sequencing platform, to further study the HIV-1 mRNAs population in HIV-1 transfected or infected HeLa cells, as well as in infected CD4 T cells. 1D cDNA sequencing of HeLa samples provided between 313,695 and 481,824 reads that could be aligned uniquely either to the human or to the NL4.3-HIV-1 genome. Viral reads represented 2.5 to 3.4% of the total reads with the longest sequenced stretch of HIV cDNA of 9038 bp. Between 2960 to 10,672 reads with a mean read length of 1338.6 to 1439.5 nt were used to deconvolute each individual HIV-1 isoform unambiguously, giving for the first time a full picture of the representation of each isoform in HIV-1 expressing samples. Several splice junctions that were recently identified in other strains were confirmed in NL4.3 strain. Differences between transfected and infected cells, as well as differences in between cell types were studied. Finally, we assessed the performance of MinION sequencing technology to quantitativaly compare HIV-1 isoforms between a wild type sample and a sample in which splicing has been artificially disturbed.

This work shows that MinION sequencing is a powerful tool to study in details the composition of HIV-1 mRNA population in cells.

**Keywords:** HIV-1 splicing; RNA isoforms; MinION; Long read sequencing

#### O14 The Genomic Distribution of Stably Expressed Proviruses

##### Dalibor Miklík, Filip Šenigl, Miroslav Auxt, Jiří Hejnar

###### Laboratory of Viral and Cellular Genetics, Institute of Molecular Genetics, Academy of Sciences of the Czech Republic, Prague, Czech Republic

**Correspondence:** Dalibor Miklík

*Retrovirology* 2018, **15(Suppl 1)**:O14

Retroviral integration is known to be nonrandom and biased toward the genomic regions with certain characteristics. The epigenetic landscape at the site of integration can substantially affect the transcriptional activity of the provirus but the relation between targeted genomic loci and the proviral activity remains poorly understood. In our studies we focus on the characterization of the proviral integration sites of long-term stably active proviruses.

We examined the expression of retroviral vectors in ENCODE Tier1 human K562 cell line. Using minimal vectors derived from avian sarcoma leukosis virus (ASLV), murine leukemia virus (MLV) and human immunodeficiency virus type 1 (HIV) we compared various retroviral genera with different integration preferences. Tracking expression of vector-carried EGFP in cellular clones derived from EGFP-positive cells we evaluated the stability of the proviral expression and identified proviral integration sites in clones with stable expression of EGFP during long-term in vitro culture.

ASLV is prone to the provirus silencing, but in the rare clones that keep stable EGFP expression for at least 60 days, the proviruses were accumulated in short distance downstream to active promoters [1, 2]. This sharp distribution of stably active ASLV proviruses was released to more distal parts of gene bodies when additional CpG island core element is inserted into ASLV long terminal repeat region. More frequent stably active proviruses of CpG island-modified ASLV were, however, still close to other regulatory elements—enhancers. Comparing the integration sites of stably expressed ASLV- MLV- and HIV-derived vectors we found that the proximity to enhancer areas are common feature observed for long-term active proviruses [3].

From our data we conclude that the genomic areas around active promoters are the permissive sites for silencing-prone promoters driving the long-term transgene expression. We also observe stably active proviruses of different origin to be associated with the enhancer regions. Our results point out the importance of considering the integration site in retroviral vector applications where stable transgene expression is the desired outcome.

**Keywords:** Vectors; Integration; Stable expression; Epigenetics

**References**

1. Šenigl F., Auxt M., Hejnar J., *Nucleic Acids Research* Volume 40, Issue 12 **2012**, 5298–5312.
2. Šenigl F., Miklík D., Auxt M., Hejnar J., *Nucleic Acids Research*, Volume 45, Issue 22 **2017**, 12752–12765.
3. Miklík D., Šenigl F., Hejnar J., *Viruses*, 10(3) **2018**, 116.

#### O15 The Biochemistry of HIV-1 Uncoating and Nuclear Import

##### Felipe Diaz-Griffero

###### Department of Microbiology and Immunology, Albert Einstein College of Medicine, NY, USA

**Correspondence:** Felipe Diaz-Griffero

*Retrovirology* 2018, **15(Suppl 1)**:O15

The HIV-1 core is composed of ~ 1800 monomers of capsid assembled in a conical structure. Upon viral membrane fusion, the HIV-1 core is delivered into the cytoplasm, where the uncoating process of the virus takes place. Uncoating is biochemically defined as the dissociation of monomeric capsids from the HIV-1 core over time. Over the years, we have developed assays to measure uncoating, binding to capsid, and capsid stability. Our investigations have revealed that uncoating is linked to reverse transcription by using TRIM5alpha RING domain mutants, and reverse transcription inhibitors. In addition, we showed that the surface of the HIV-1 core is dynamic and could expose and hide proteins domains, depending upon changes within or outside the core. The process of exposing and hiding domains on the surface of the core creates a communication system between the inside and the outside. Using capsid binding assays, we found that inhibition of HIV-1 by NES-CPSF6 and MxB correlates with the ability of these proteins to bind capsid. Using the fate of the capsid assay, we have correlated inhibition of HIV-1 by NES-CPSF6 and MxB with inhibition of uncoating (stabilization of the core). These experiments suggested that stability of the core is important for productive infection. For example, increase or decrease stability of the HIV-1 core causes changes in infectivity. Our capsid binding assay showed that drugs such PF74 and BI-2 prevents the binding of CPSF6 to capsid. Interestingly, the use of PF74 and BI-2 in the fate of the capsid assay showed that these drugs accelerate uncoating, in a way mimicking the effects of TRIM5alpha and TRIMCyp. Using our capsid binding assay, we showed that the HIV-1 core binds to the nucleopore component Nup153 in an FG-dependent manner, and this binding is sensitive to PF74 and BI2, suggesting that these drugs not only prevent the binding of CPSF6 to the core but also several other host proteins. These results suggested that preventing the binding of host proteins to the core in the early steps of replication destabilizes the core terminating the infection. Destabilization of the core is achieved by missing interactors or the availability of interactors that destabilize the core such as TRIM5alpha. Stabilization of the core is achieved by specific interactors or by the modulation of events inside the core such as reverse transcription. Biochemical models of uncoating and nuclear import will be discussed.

**Keywords:** HIV-1; Uncoating; Nuclear Import; Capsid binding; Fate of the capsid

### Session 4: Pathogenesis and evolution

#### O16 Temporary early ART limits the viral reservoir but increases virus diversity

##### Yara L. Verschoor^1^, Jelmer Vroom^1^, Jan M. Prins^2^, Ben Berkhout^1^, Alexander O. Pasternak^1^

###### ^1^Laboratory of Experimental Virology, University of Amsterdam, The Netherlands; ^2^Department of Internal Medicine, Academic Medical Center of the University of Amsterdam, Amsterdam, The Netherlands

**Correspondence:** Alexander O. Pasternak

*Retrovirology* 2018, **15(Suppl 1)**:O16

Early initiation of ART is one of the most promising strategies for an HIV cure. Temporary ART initiated during primary HIV-1 infection (PHI) lowers the virological set point and defers the restart of ART during chronic infection (CHI). To elucidate the mechanisms behind these effects, we measured the virus diversity and reservoir size in patients treated with temporary ART during PHI. Levels and HIV genetic diversity of plasma viral RNA, cell-associated (CA) HIV RNA and DNA were analyzed in HIV-infected patients who had participated in a randomized controlled trial of 24 or 60 weeks of temporary ART versus no treatment during PHI [1] and subsequently (re)started ART during CHI after a median of 2.5 years without treatment. We performed single-genome sequencing of HIV-1 p6-PRO-RT region (1.42 kb) and estimated nucleotide and amino acid diversities by computing mean pairwise distances. First, we compared the on-ART proviral diversities in the same patients and at the same time points on ART between PHI and CHI ART periods. CA HIV DNA diversity was significantly lower during PHI ART than during CHI ART (p = 0,023, Wilcoxon signed rank test). Secondly, we measured levels of plasma HIV RNA, CA HIV RNA, and CA HIV DNA at the virological set point (36 weeks after ART interruption or randomization). Levels of all these markers were significantly lower in patients who had been treated with temporary ART as compared to the untreated patients. No significant difference was observed in the HIV nucleotide diversity of these markers between the treated and untreated patients. Surprisingly, the amino acid diversities of plasma HIV RNA (PRO) and CA DNA (PRO-RT) were significantly higher in treated vs. untreated patients (p = 0.005 and p = 0.028, respectively, Mann–Whitney test). These effects were not mediated by drug-resistance mutations as none were found in PRO or RT in any patient after early ART interruption. In summary, temporary early ART resulted in lower HIV reservoir but higher virus diversity after treatment interruption compared to the patients who did not receive early ART. Early ART might have augmented HIV-specific cellular immune responses, resulting in the development of immune escape HIV variants with reduced viral fitness upon ART interruption.

**Keywords:** ART; Virus diversity; Viral reservoir; Primary HIV-1 infection

**Reference**

1. Grijsen ML, Steingrover R, Wit FW, et al. No treatment versus 24 or 60 weeks of antiretroviral treatment during primary HIV infection: the randomized Primo-SHM trial. *PLoS Med*. **2012**;9(3):e1001196.

#### O17 HIV-1 Isolates from West Africa Show Higher Fitness than to those from East Africa

##### Omotayo Farinre^1^, Kamini Gounder^1,2^, Marcel Tongo^3^, Jonathan Hare^4^, Jill Gilmour^4^, Jaclyn Mann^1^, Thumbi Ndung’u^1,2,5,6^

###### ^1^HIV Pathogenesis Programme (HPP), Nelson R. Mandela School of Medicine, UKZN; ^2^Africa Health Research Institute (AHRI), Nelson R. Mandela Medical School, UKZN; ^3^KwaZulu-Natal Research Innovation & Sequencing Platform (KRISP), Nelson R. Mandela Medical School, UKZN; ^4^Human Immunology Laboratory, International AIDS Vaccine Initiative (IAVI), Imperial College of Science, Technology and Medicine, London, UK; ^5^Ragon Institute of MGH, MIT and Harvard University, Cambridge, USA; ^6^Max Planck Institute for Infection Biology, Berlin, Germany

**Correspondence:** Omotayo Farinre

*Retrovirology* 2018, **15(Suppl 1)**:O17

The HIV-1 epidemic in sub-Saharan Africa is heterogeneous with diverse viral subtypes that are unevenly distributed and differing prevalence across regions. The biological relevance of this diversity has implications for developing preventive and therapeutic interventions. West Africa has a prevalence rate of 2% with one dominant CRF, whereas East Africa has a prevalence rate of 5%, with subtype A1 predominant and multiple CRFs identified. We explored whether there are biological differences that may explain the characteristic epidemic spread between both regions.

Phylogenetic and functional analyses of the Gag-protease region were used to characterize HIV-1 subtypes from ART-naïve plasma samples from ineligible blood donors and a village population in Cameroon (n = 91) and a general population that included discordant couples, heterosexuals and MSMs in Kenya, Uganda and Tanzania (n = 162). Replication capacity of patient-derived chimeric viruses generated was measured using a green fluorescent reporter-based cell assay.

CRF02_AG (55%), other recombinants (26%), pure subtypes (19%) were identified in West Africa. In East Africa, we identified subtypes A1 (57%), D (19%), AD (15%) and AC (6%). Viruses encoding Gag sequences derived from West Africa had a significantly higher replication capacity overall (0.95 ± 0.02) than those encoding Gag (0.84 ± 0.03) sequences derived from East Africa (p = 0.0017). Furthermore, Gag inter-subtype recombinant viruses from West Africa had a significantly higher replication capacity (0.97 ± 0.02) than inter-subtype recombinant viruses from East Africa (0.85 ± 0.04) (p = 0.0053).

Recent studies suggest that HIV-1 subtypes with lower replication capacity are preferentially transmitted and are expanding more rapidly. Our data is consistent with this hypothesis as Gag sequences from East Africa, where the HIV prevalence is higher, were overall less fit in vitro than those from West Africa. These findings may account in part for the low HIV prevalence in West Africa.

**Keywords:** HIV-1; Subtype-Diversity; Sub-Saharan Africa; Replication capacity

#### O18 HIV-1 infected T cell profiling reveals dysregulation of a putative NK receptor

##### Sandra Dehn^1^, Herwig Koppensteiner^2^, Sebastian Bolduan^1^, Marius Codrea^3^, Carolina Russo^2^, Tanja Bauer^2^, Sven Nahnsen^3^, Rika Draenert^4^, Michael Schindler^1,2,*^

###### ^1^Institute of Medical Virology and Epidemiology of Viral Diseases, University Hospital Tübingen, Tübingen, Germany; ^2^Institute of Virology, Helmholtz Zentrum Munich, German Research Center for Environmental Health, Neuherberg, Germany; ^3^Center for Quantitative Biology (QBiC), Eberhard Karls University Tübingen, Tübingen, Germany; ^4^Medical Clinic and Policlinic IV, Ludwig-Maximilians-University Munich, Munich, Germany

**Correspondence:** Michael Schindler

*Retrovirology* 2018, **15(Suppl 1)**:O18

HIV-1 potently evades the host immune response and a main mechanism of viral immune evasion is to manipulate plasma membrane immune receptors. To obtain an unbiased overview of the membranous fingerprint and the modulation of various membrane receptors in the course of HIV-1 infection, we established a flow cytometry based medium-throughput screening procedure. By this strategy, we profiled the expression levels of 332 membrane receptors on the cell surface of productively HIV-1 infected primary CD4+ T cells in direct comparison to uninfected cells.

We were able to confirm plasma membrane receptors already described in the literature and discovered a variety of previously unknown or poorly described receptors which are modulated by HIV-1. One of our most striking and significant hits is a receptor recently characterized as essential for NK cell response. Interestingly, the magnitude of HIV-1 mediated NK cell receptor modulation was much more pronounced in primary T cells as the effect of HIV-1 on CD155 and NTB-A, other NK cell receptors previously suggested to be modulated by HIV-1. Our mechanistic investigations revealed that the NK cell receptor is internalized and degraded by the concerted action of the HIV-1 accessory proteins Nef and Vpu. While Nef leads to receptor internalization and sequestration in a perinuclear compartment, Vpu induced intracellular degradation, presumably through the lysosomal compartment. A comprehensive mutagenesis approach revealed that NK cell receptor internalization and degradation was independent of known motifs in Nef and Vpu important for dysregulation of for instance Tetherin, CD4 and MHCI. Furthermore the antagonizing activity of Nef and Vpu was conserved between lentiviral Nef and Vpu proteins of various primate lentiviruses and most pronounced in primary alleles, suggesting an ongoing evolutionary pressure on this function.

The functional role of this receptor has only been characterized for NK cells, but so far nothing is known about its role on CD4+ T cells. Therefore, we currently perform comprehensive OMICs with overexpression and knockout cells to elucidate the functional relevance of this receptor and to thereby possibly identify a novel mechanism of how HIV-1 evades the immune system.

**Keywords:** HIV-1; Receptor modulation; Immune evasion; NK cell response

#### O19 Effect of HIV-1 subtype C LTR genetic variation on disease outcome

##### P Madlala^1^, S Nzimande^1^, E Blom^1^, N Masango^1^, T Ndung’u^1,2,3^

###### ^1^HIV Pathogenesis Programme (HPP), Nelson R Mandela School of Medicine, University of KwaZulu-Natal, Durban, South Africa; ^2^Ragon Institute of Massachusetts General Hospital, Massachusetts Institute of Technology and Harvard University, Boston, Massachusetts, USA; ^3^Africa Health Research Institute (AHRI), University of KwaZulu-Natal, Durban, South Africa

**Correspondence:** P. Madlala

*Retrovirology* 2018, **15(Suppl 1)**:O19

The human immunodeficiency virus type 1 (HIV-1) 5’ long terminal repeat (LTR) is a promoter that drives viral gene transcription. Inter- and intra-subtype LTR genetic variation has been observed in different populations. The HIV-1 LTR is divided into unique 3 (U3), repeat (R) and unique 5 (U5) regions. The U3 region is further divided into core promoter, enhancer and modulatory domains that contains the transcription factor binding sites, which are important for LTR transcription activity. Interestingly, 3 or 4 NF-κβ binding sites (BS) present only in HIV-1 subtype C LTR (C-LTR) makes C-LTR a stronger viral promoter than other subtype LTRs that contain two or one such sites. Therefore manipulation of HIV-1 LTR provides a potential therapeutic strategy for suppressing or inducing latent viral gene expression. However, genetic variation of the HIV-1 LTR and its impact on disease outcome has not been well characterized in acute infection. We hypothesize that HIV-1 LTR genetic variation during acute infection may modulate viral replication and disease outcome.

Viral RNA was extracted from plasma samples obtained from 14 patients in the HIV Pathogenesis Programme (HPP) acute infection cohort at two time points, within 3 weeks of infection (B2) and at approximately 1 year post infection (B7), using the QIAGEN Viral RNA Mini kit and reverse transcribed into viral DNA using SuperScript III OneStep kit. Nested PCR was performed to specifically amplify U3 region of HIV-1 LTR using the KAPA HiFi PCR kit. Nested PCR products were sequenced using the BigDye cycle sequencing kit v3.1 and Phylogenetic relatedness to compare and evaluate intra- and inter-patient diversity was performed by Neighbour-Joining trees with 1000 bootstrap replicates.

Sequences from all 14 patients were confirmed as HIV-1 subtype C due to the presence of ≥ 3 NF-κβ BS and strong bootstrap value. The B2 and B7 sequences from the same patients showed monophyletic groups. Our data show that 3/14 patients were infected with viruses that contained 4 NF-κβ BS. However, there was non-significant trend towards low viral loads in patients infected with 4 NF-κβ BS viruses (p = 0.11), probably due to very small number and the presence of detrimental mutation in either the TATA box (TA**T**AA to TA**A**AA), Sp1i (GGGGAGTGGT**C** to GGGGAGTGGT**T**) or RBE III (ACTG**C**TGACA to ACTG**A**TGACA) BS.

More samples to be sequenced and HIV-1 LTR transcriptional activity to be assessed.

**Keywords:** HIV-1; LTR; Variation; Acute infection

### Session 5: Restriction/innate immunity

#### O20 HIV-1 exploits cell–cell spread to modulate resting CD4 T cell permissivity to infection

##### Ann-Kathrin Reuschl, Maitreyi Shivkumar, Dejan Mesner, Xenia Snetkov, Tafhima Haider, Clare Jolly

###### Division of Infection and Immunity, University College London, 90 Gower Street, London, WC1E 6BT, UK

**Correspondence:** Ann-Kathrin Reuschl

*Retrovirology* 2018, **15(Suppl 1)**:O20

*In vivo*, HIV-1 and its reservoir can be detected in resting CD4 T cells. *In vitro*, however, non-activated T cells are refractory to the virus and require mitogenic stimulation to become permissive to cell-free infection. This discrepancy in reported permissivity poses a major challenge for current research and hampers functional studies of host–pathogen interactions in HIV-1. The role of cell–cell spread, the most efficient form of viral dissemination, remains largely unaddressed in the context of this paradoxical observation.

Through a global phospho-proteomic SILAC-screen, we recently revealed dynamic changes to intracellular signalling in infected and uninfected T cell populations during cell–cell spread of HIV-1[1]. These findings led us to hypothesise that signalling induced at the virological synapse may overcome intracellular restriction of HIV-1 in resting CD4 T cells.

To address this, we co-cultured HIV-1 infected primary T cells with non-activated autologous target CD4 T cells to monitor HIV-1 dissemination into resting cells. Our data reveals that, unlike cell-free infection, cell–cell spread from infected to uninfected T cells allows HIV-1 to efficiently access resting T cells leading to productive infection without the need for artificial activation. HIV-1 hijacks cell–cell contacts to propagate infection and drives plasticity of resting CD4 T cells in an envelope-fusion dependent manner. Interestingly, resting memory CD4 T cells are preferentially targeted by this mode of HIV-1 spread, which is in strong agreement with HIV-1 pathology.

We propose that signalling at the virological synapse allows HIV-1 to exploit cell–cell spread by conditioning uninfected resting CD4 T cells for infection, thereby shaping the immunological niche in which the virus resides.

**Keywords:** Resting T cells; Cell–cell spread; HIV-1; Permissivity

**Reference**

1. Len A., Starling S., Shivkumar M., Jolly C., *Cell Reports,* Volume 18, Issue 4, p1062–1074.

#### O21 Rev-dependent Expression of HIV-1 Intron-containing RNA Induces Type I IFN Responses

##### Hisashi Akiyama, Caitlin M. Miller^2^, Chelsea R. Ettinger^1^, Rahm Gummuluru^1^

###### ^1^Department of Microbiology, Boston University School of Medicine, Boston, MA, USA; ^2^Department of Pathology, Boston University School of Medicine, Boston, MA, USA

**Correspondence:** Hisashi Akiyama

*Retrovirology* 2018, **15(Suppl 1)**:O21

A hallmark of HIV-1 infection in vivo is systemic chronic immune activation. While in vivo studies have suggested chronic low-level production of type I interferon (IFN-I) as the driving force for aberrant immune activation, the molecular mechanisms for persistent IFN-I signaling have remained unclear. Myeloid cells play important roles in production of IFN-I upon pathogen sensing, and are found persistently infected with HIV-1 in various tissues of HIV-1^+^ individuals, even on prolonged cART. Therefore, in this study, we examined the hypothesis that HIV-1 infection of myeloid cells induces IFN-I responses and contributes to chronic immune activation.

Monocyte-derived macrophages (MDMs) were derived from CD14^+^ cells isolated from PBMCs and were infected with HIV-1, and expression of CD169, a myeloid cell specific interferon stimulated gene (ISG), and IP-10 was measured as markers of immune activation. Establishment of productive HIV-1 infection in MDMs resulted in robust upregulation of CD169 and IP-10, which was abrogated upon treatment of MDMs with HIV-1 entry, reverse transcription, integration or viral transcription inhibitors or upon treatment with an IFN-I neutralizing reagent (B18R), suggesting that activation of MDMs is dependent on de novo viral gene expression and is mediated by soluble IFN-I. Infection of MDMs with a panel of HIV-1 mutants revealed that none of HIV-1 structural or accessory protein expression was required for MDM activation. Interestingly, we found that Rev–CRM1-dependent nuclear export of HIV-1 intron-containing RNA was necessary for ISG induction and MDM activation, while CTE–TAP-dependent export of HIV-1 intron-containing RNA failed to induce MDM activation. Moreover, alteration of intra-cytoplasmic trafficking of HIV-1 intron-containing RNA, and relocalization away from peripheral membrane sites attenuated innate immune activation of MDMs, suggesting that not only expression but also trafficking itinerary of HIV-1 intron-containing RNA in cytoplasm affects detection by a host nucleic acid sensor and induction of IFN-I responses in MDMs.

In conclusion, our findings suggest that persistent expression of HIV-1 intron-containing RNA including aberrant transcripts observed in HIV-1^+^ individuals on cART is subject to localization-dependent sequence-independent sensing mechanism, which contributes to chronic immune activation in vivo.

**Keywords:** Innate immune activation; Myeloid cells; Intron-containing RNA; RNA trafficking

#### O22 Cell cycle-dependent regulation of SAMHD1 dephosphorylation at T592

##### Kerstin Schott^1^, Nina V. Fuchs^*,1^, Rita Derua^*,2^, Bijan Mahboubi^3^, Esther Schnellbächer^1^, Janna Seifried^1^, Christiane Tondera^1^, Heike Schmitz^1^, Caitlin Shepard^3^, Alberto Brandariz-Nuñez^4^, Felipe Diaz-Griffero^4^, Andreas Reuter^5^, Baek Kim^3,6^, Veerle Janssens^2^, Renate König^1,7^

###### ^1^Paul-Ehrlich-Institut, Host-Pathogen Interactions, Langen, Germany; ^2^Department of Cellular and Molecular Medicine, KU Leuven, Leuven, Belgium; ^3^Children’s Healthcare of Atlanta, Center for Drug Discovery, Department of Pediatrics, Emory University, Atlanta, USA; ^4^Department of Microbiology and Immunology, Albert Einstein College of Medicine, Bronx, USA; ^5^Paul-Ehrlich-Institut, Division of Allergology, Langen, Germany; ^6^Department of Pharmacy, Kyung-Hee University, Seoul, South Korea; ^7^SBP Medical Discovery Institute, La Jolla, USA

**Correspondence:** Kerstin Schott

*Retrovirology* 2018, **15(Suppl 1)**:O22

*Contributed equally

SAMHD1-mediated restriction of HIV-1 replication is part of the antiretroviral defense program. This restriction is strictly controlled by dephosphorylation of T592 in SAMHD1. We demonstrate that SAMHD1 dephosphorylation at T592 happens in proliferating cells during mitotic exit, an important transition between M and G_1_ phase. Concomitantly, we observed a pronounced drop of all four dNTPs in G_1_ compared to S and G_2_/M phase. Strikingly, as soon as cycling HeLa cells enter G_1_ phase, HIV-1 infection resulted in reduction of RT products dependent on the presence of dephosphorylated SAMHD1. To validate that SAMHD1 could be antivirally active in the G_1_ phase of HIV-1 target cells, we infected primary CD4^+^ T cells and arrested them in mitosis using nocodazole. Interestingly, we detected higher RT products in mitotic cells, displaying high SAMHD1 pT592-levels, compared to CD4^+^ T cells that entered G_1_ phase (accompanied by SAMHD1 dephosphorylation at T592) after nocodazole wash-out. Intriguingly, a less pronounced difference in RT products could be observed in the presence of Vpx, after degradation of SAMHD1.

Using different proteomics approaches, cell-based and in vitro-biochemical assays, we revealed the phosphatase holoenzyme PP2A-B55α to dephosphorylate SAMHD1 at T592. PP2A-B55α is a key mitotic exit phosphatase in mammalian cells, supporting the conclusion that T592 in SAMHD1 is a mitotic substrate of PP2A-B55α. Moreover, we identified basic residues flanking T592 in SAMHD1, a known recognition motif shared only by B55 substrates, responsible for interaction with PP2A-B55α.

We propose the following model: In proliferating cells, the G_1_ stage of the cell cycle represents a short window where SAMHD1 could be antivirally active. SAMHD1 is dephosphorylated at T592 by PP2A-B55α trimers in cycling cells every time cells exit mitosis. As a consequence, active SAMHD1 could inhibit reverse transcription of HIV-1 in G_1_ phase. Whether reduced cellular dNTP pools or other dNTPase-independent functions, triggered upon mitotic exit, are responsible for HIV-1 inhibition in G_1_ phase, will need to be determined. By contrast, SAMHD1 is phosphorylated from S to M phase and rendered inactive against HIV-1. We speculate that HIV-1-infected cells are reprogrammed to avoid progression to the end of mitosis, where PP2A-B55α would be active and remove CDK-mediated phosphorylations. Thereby, HIV-1 actively evades dephosphorylated, restrictive SAMHD1 present in G_1_ phase in cycling cells.

**Keywords:** Restriction factors; SAMHD1; Phospho-regulation; PP2A

#### O23 HIV-1 Vpr counteracts CTIP2-mediated cellular response to viral infections

##### F. Forouzanfar^1^, S. Ali^1,2^, C. Ducloy^3^, H. El Mekdad^1^, M. El Maassarani^1^, A. Aït-Ammar^1^, C. Wallet^1^, M. De Rovere^1^, E. Boutant^4^, F. Daouad^1^, F. Margotin-Goguet^5,6,7^, C. Moog^3^, C. Van Lint^8*^, C. Schwartz^1*^, O. Rohr^1*^

###### ^1^Université de Strasbourg, Louis Pasteur, Schiltigheim, France; ^2^Institute of Microbiology, University of Agriculture, Faisalabad, Pakistan; ^3^Fédération de médecine translationnelle (FMTS), Université de Strasbourg, Strasbourg, France; ^4^Université de Strasbourg, Illkirch, France; ^5^Institut Cochin, Paris, France; ^6^CNRS, UMR8104, Paris, France; ^7^Université Paris Descartes, Sorbonne Paris Cité, Paris, France; ^8^Department of Molecular Biology, Université Libre de Bruxelles, Gosselies, Belgium

**Correspondence:** C. Schwartz

*Retrovirology* 2018, **15(Suppl 1)**:O23

Associated with chromatin-modifying enzymes, the cellular cofactor CTIP2 contributes to HIV-1 gene silencing in latently infected reservoirs [1, 2, 3] that constitute the major block toward an HIV cure. We find that productive infections of CD4+ T cells result in a deep decrease of CTIP2 expression suggesting that CTIP2 may contribute to the cellular response to a viral aggression. Indeed, we show that CTIP2 expression is induced by interferon alpha to repress HIV-1 gene transcription and strength the cellular antiviral response. However, we found that the virus has developed a strategy to overcome this major transcriptional block. Productive HIV-1 infection resulted in deep CTIP2 depletion mediated by the viral accessory protein Vpr [4]. Associated to the Cul4A-DDB1-DCAF1 ubiquitin ligase complex, Vpr targets CTIP2 to degradation via the proteasome pathway. Interestingly, Vpr specifically targets CTIP2 proteins associated with heterochromatin-promoting enzymes, therefore impeding transcriptional silencing and favoring HIV-1 replication.

Altogether, our results (i) strengthen that Vpr is a key virulence factor and, (ii) suggest that CTIP2 constitutes the first restriction factor targeting the transcription step of the HIV-1 life cycle.

**Keywords:** HIV-1; CTIP2; Vpr; Restriction factor

**References**

1. Cherrier, T. et al. CTIP2 is a negative regulator of P-TEFb. Proc Natl Acad Sci U S A, 110, 12655–60 (2013)
2. Marban, C. et al. Recruitment of chromatin-modifying enzymes by CTIP2 promotes HIV-1 transcriptional silencing. Embo J, 26, 412–23 (2007).
3. Marban, C. et al. COUP-TF interacting protein 2 represses the initial phase of HIV-1 gene transcription in human microglial cells. Nucleic Acids Res, 33, 2318–31 (2005).
4. Lahouassa H et al. HIV-1 Vpr degrades the HLTF DNA translocase in T cells and macrophages. Proc Natl Acad Sci U S A, 113(19):5311–6 (2016)

### Session 6: Other retroviruses

#### O24 New insights into Bovine Leukemia Virus (BLV) transcriptional regulation

##### Maxime Bellefroid^*^, Estelle Plant^*^, Anthony Rodari, Benoit Van Driessche, Sylvain Fauquenoy, Caroline Vanhulle, Lorena Nestola, Arsène Burny, Carine Van Lint

###### Service of Molecular Virology, Department of Molecular Biology, Université Libre de Bruxelles, Belgium

**Correspondence:** Maxime Bellefroid

*Retrovirology* 2018, **15(Suppl 1)**:O24

*These authors contributed equally to this work

Bovine leukemia virus (BLV) is a B-lymphotropic oncogenic deltaretrovirus infecting cattle and closely related to human T-cell leukemia viruses I and II (HTLV-I and II). Despite the well-established repression of the 5’LTR-driven viral gene expression, we and others have discovered and characterized two alternative viral promoters [1, 2, 3, 4], allowing a high expression of viral miRNAs [2, 3] and antisense viral transcripts [4], potentially contributing to tumor development. In addition, our data have suggested a collision phenomenon between the RNAPIII transcribing the miRNA cluster and the RNAPII coming in an antisense orientation from the 3’LTR [1]. These latter results have indicated that transcriptional interference could be seen as a new mechanism used by BLV to regulate its three transcriptional activities.

In this work, we investigated the interplay between the three BLV promoter activities and showed putative critical functions of the transcriptional interference to drive or repress BLV transcriptional activities. In addition, we highlighted the implication of new transcription factors in BLV transcriptional and epigenetic regulations but also in BLV-mediated pathogenesis.

Overall in this study, we further investigated new alternative ways used by BLV to regulate its transcriptional and epigenetic status and provided new fundamental insights into BLV transcriptional and epigenetic regulations which could explain the escape from the host immune system and/or the BLV-induced pathogenesis.

**Keywords:** Bovine Leukemia Virus; Transcription; Epigenetic; Regulation

**References**

1. Van Driessche B., Rodari A., Delacourt N., Fauquenoy S., Vanhulle C., Burny A., Rohr O., Van Lint C., *Characterization of new RNA polymerase III and RNA polymerase II transcriptional promoters in the Bovine Leukemia Virus genome*, Sci Rep 6, **2016**.
2. Rosewick N., Momont M., Durkin K., Takeda H., Caiment F., Cleuter Y., Vernin C., Mortreux F., Burny A., Georges M., and Van den Broeke A., *Deep sequencing reveals abundant noncanonical retroviral microRNAs in B*-*cell leukemia/lymphoma,* PNAS, 110, **2013**.
3. Kincaid R. P., Burke J. M., Sullivan C. S., *RNA virus microRNA that mimics a B*-*cell oncomiR*, PNAS, 109, **2012**.
4. Durkin K., Rosewick N., Artesi M., Hahaut V., Griebel P., Burny A., Georges M., Van den Broeke A., *Characterization of novel Bovine leukemia Virus (BLV) antisense transcripts by deep sequencing reveals constitutive expression in tumors and transcriptional interaction with viral a microRNAs,* Retrovirology 13:33, **2016.**

#### O25 Apolipoprotein E is an HIV-1-inducible inhibitor of viral production and infectivity in macrophages

##### Yasuo Ariumi^1^, Rokeya Siddiqui^1^, Mikinori Ueno^1^, Hesham Nasser^1^, Ryota Koba^1,2^, Farzana Bhuyan^1^, Osamu Noyori^1^, Mariko Yasuda-Inoue^1^, Takayuki Hishiki^3^, Sayaka Sukegawa^4^, Eri Miyagi^4^, Klaus Strebel^4^, Shuzo Matsushita^1^, Kunitada Shimotohno^5^, Shinya Suzu^1^

###### ^1^Center for AIDS Research, Kumamoto University, Kumamoto, Japan; ^2^Laboratory of Veterinary Microbiology, Department of Veterinary Medicine, Nihon University, Fujisawa, Kanagawa, Japan; ^3^Department of Microbiology and Cell Biology, Tokyo Metropolitan Institute of Medical Science, Tokyo, Japan; ^4^National Institute of Allergy and Infectious Diseases, National Institutes of Health, Bethesda, Maryland, USA; ^5^Research Center for Hepatitis and Immunology, National Center for Global Health and Medicine, Chiba, Japan

**Correspondence:** Yasuo Ariumi

*Retrovirology* 2018, **15(Suppl 1)**:O25

Apolipoprotein E (ApoE) belongs to a class of cellular proteins involved in lipid metabolism. ApoE is a polymorphic protein produced primarily in macrophages and astrocytes. Different isoforms of ApoE have been associated with susceptibility to various diseases including Alzheimer’s and cardiovascular disease. ApoE expression has also been found to affect susceptibility to several viral diseases, including Hepatitis C and E, but its effect on the life cycle of HIV-1 remains obscure. In this study, we initially found that HIV-1 infection selectively up-regulated ApoE in human monocyte-derived macrophages (MDMs). Interestingly, ApoE knockdown in MDMs enhanced the production and infectivity of HIV-1, and was associated with increased localization of viral envelope (Env) proteins to the cell surface. Consistent with this, ApoE over-expression in 293T cells suppressed Env expression and viral infectivity, which was also observed with HIV-2 Env, but not with VSV-G Env. Mechanistic studies revealed that the C-terminal region of ApoE was required for its inhibitory effect on HIV-1 Env expression. Moreover, we found that ApoE and Env co-localized in the cells, and ApoE associated with gp160, the precursor form of Env, and that the suppression of Env expression by ApoE was cancelled by the treatment with lysosomal inhibitors. Overall, our study revealed that ApoE is an HIV-1-inducible inhibitor of viral production and infectivity in macrophages that exerts its anti-HIV-1 activity through association with gp160 Env via the C-terminal region, which results in subsequent degradation of gp160 Env in the lysosomes.

**Keywords:** HIV-1; Apolipoprotein E; Macrophage; Restriction factor

#### O26 SAMHD1 suppresses the NF-κB and interferon pathways induced by viral infections

##### Serena Bonifati^1^, Shuliang Chen^1,2^, Zhihua Qin^1^, Corine St. Gelais^1^, Karthik M. Kodigepalli^1^, Bradley S. Barrett^3^, Sun Hee Kim^1^, Jenna M. Antonucci^1^, Katherine J. Ladner^4,5^, Olga Buzovetsky^6^, Kirsten M. Knecht^6^, Yong Xiong^6^, Jacob S. Yount^7^, Denis C. Guttridge^4,5^, Mario L. Santiago^3^, Li Wu^1,4,7^

###### ^1^Center for Retrovirus Research, Department of Veterinary Biosciences, Ohio State University, Columbus, OH, USA; ^2^School of Basic Medical Sciences, Wuhan University, Wuhan, P.R. China; ^3^Department of Medicine, University of Colorado School of Medicine, Aurora, Colorado, USA; ^4^Department of Cancer Biology and Genetics, Ohio State University, Columbus, OH, USA; ^5^Comprehensive Cancer Center, Ohio State University, Columbus, OH, USA; ^6^Department of Molecular Biophysics and Biochemistry, Yale University, New Haven, CT, USA; ^7^Department of Microbial Infection and Immunity, Ohio State University, Columbus, OH, USA

**Correspondence:** Serena Bonifati

*Retrovirology* 2018, **15(Suppl 1)**:O26

Sterile alpha motif and HD-domain-containing protein 1 (SAMHD1) restricts infection of retroviruses and certain DNA viruses by reducing the intracellular dNTP pool. SAMHD1 has been proposed to act as a negative regulator of interferon (IFN) and inflammatory responses to viral infections, although the molecular mechanisms of SAMHD1-mediated control of antiviral innate immunity remain unclear. We discovered that SAMHD1 suppresses the innate immune responses to viral infections and inflammatory stimuli by inhibiting nuclear factor-κB (NF-κB) activation and type I IFN (IFN-I) induction. SAMHD1 knockdown in human monocytic cells or primary macrophages infected with Sendai virus (SeV) or HIV-1, or treated with inflammatory stimuli, results in significantly higher activation of NF-κB and IFN-I signaling pathways compared to control cells. Likewise, exogenous SAMHD1 expression in HEK293T cells or SAMHD1 reconstitution in knockout THP-1 monocytic cells suppresses NF-κB activation and IFN-I induction by SeV infection or inflammatory stimuli. Mechanistically, we found that SAMHD1 blocks NF-κB activation by interacting with NF-κB1/2 and reducing phosphorylation of the NF-κB inhibitory protein IκBα. SAMHD1 also binds to the inhibitor-κB kinase ε (IKKε) and IFN regulatory factor 7 (IRF7), thus decreasing IRF7 phosphorylation by IKKε and leading to suppression of the IFN-I induction pathway. Interaction between endogenous SAMHD1 and NF-κB or IFN-I pathway proteins were confirmed in primary human macrophages. Interestingly, our data suggest that the dNTPase activity of SAMHD1 is not required for the suppressive effects. *In vivo* studies using splenocytes from *SAMHD1* knockout and heterozygous mice further confirmed suppression of NF-κB activation by SAMHD1, suggesting an evolutionarily conserved role of SAMHD1. Our findings unveil novel functions of SAMHD1 in the regulation of the innate immune response to viral infections and inflammatory stimuli, and provide insights for the potential use of SAMHD1 as a therapeutic target for the treatment of viral infections.

**Keywords:** SAMHD1; Type I interferon; NF-κB; Viral infection.

#### O27 Antagonism of HUSH complex by HIV-2/SIVsmm Vpx and some Vpr lentiviral proteins

##### Ghina Chougui^1,2,3^, Soundasse Munir-Matloob^1,2,3^, Roy Matkovic^♯,1,2,3^, Michael Martin^♯,1,2,3^, Marina Morel^♯,1,2,3^, Hichem Lahouassa^1,2,3^, Marjorie Leduc^1,2,3,4^, Bertha Cecilia Ramirez^1,2,3^, Lucie Etienne^5^, Florence Margottin-Goguet*^,1,2,3^

###### ^1^Inserm, U1016, Institut Cochin, Paris, France; ^2^CNRS, UMR8104, Paris, France; ^3^Université Paris Descartes, Sorbonne Paris Cité, Paris, France; ^4^3P5 proteomic facility of Paris Descartes University, Paris, France; ^5^CIRI – International Center for Infectiology Research, Inserm U1111, Université Claude Bernard Lyon 1, CNRS UMR5308, Ecole Normale Supérieure de Lyon, Univ Lyon, Lyon, France

**Correspondence:** Florence Margottin-Goguet

*Retrovirology* 2018, **15(Suppl 1)**:O27

^♯^Contributed equally to the work

HIV-1 and HIV-2 are both responsible for AIDS, however HIV-2 may be characterized by low viremia and a higher tendency for latency compared to HIV-1. The HIV-2/SIVsmm lineage displays a viral protein with no equivalent in HIV-1/SIVcpz, Vpx that helps overcome an early post-entry block through SAMHD1 degradation. Though Vpx is absolutely required in myeloid cells, its deletion impairs replication in some PBMC and primary T cells, where SAMHD1 restriction does not operate. In addition, the existence of unidentified restriction factors counteracted by Vpx has been proposed in different settings.

Through a large-scale proteomic screen, we identified a new Vpx target: HUSH (« Human Silencing Hub » containing TASOR, MPP8 and Periphilin), a complex involved in position-effect variegation and epigenetic silencing of Line-1 transposable elements. Down-regulation of this host complex is observed in VLP treated primary cells and HIV-2 infected cells. Vpx binds HUSH and induces its proteasomal degradation, through the recruitment of the DCAF1 ubiquitin ligase adaptor, independently from SAMHD1-antagonism. As a consequence, Vpx is able to reactivate HIV latent proviruses, unlike Vpx mutants unable to inactivate the target. Furthermore, the antagonism of this epigenetic restriction is lentiviral species-specific, which is typical of molecular “arms-races” between viral antagonists and host restriction factors. Our results further suggest HUSH antagonism as an ancient function of primate lentiviruses that preceded the birth of Vpx and of SAMHD1-antagonism capacity.

Altogether, our results identify an epigenetic regulator as a lentiviral restriction factor counteracted by Vpx, therefore providing a molecular link between intrinsic immunity and epigenetic control.

This work was supported by grants from the “Agence Nationale de la Recherche sur le SIDA et les hépatites virales” (ANRS), SIDACTION, “Fondation de France” and “Fondation pour la Recherche Médicale” (FRM), amfAR, FINOVI and ANR Labex ECOFECT.

**Keywords:** Vpx; Vpr; Restriction factors; Latency

### Session 7: Novel antiviral strategies

#### O28 An RNA-binding compound that specifically blocks HIV-1 RNA encapsidation

##### Carin K. Ingemarsdotter^1^, Jingwei Zeng^1^, Ziqi Long^1^, Andrew M.L. Lever^1,2^, Julia C. Kenyon^1,3,4^

###### ^1^Department of Medicine, University of Cambridge, Cambridge, UK; ^2^Department of Medicine, National University of Singapore, Singapore; ^3^Department of Microbiology and Immunology, National University of Singapore, Singapore; ^4^Homerton College, University of Cambridge, Cambridge, UK

**Correspondence:** Carin K. Ingemarsdotter

*Retrovirology* 2018, **15(Suppl 1)**:O28

NSC260594 is a quinolinium derivative that was previously identified in a target-based assay to inhibit the interaction between the HIV-1 stem-loop 3 (SL3) RNA and Gag, displaying potent antiviral activity [1]. In this study, we investigated the effects of this compound on individual stages of the viral life cycle to ascertain whether its anti-viral activity was specific to the viral packaging stage. In addition, we studied the structural effects of NSC260594 binding to the HIV genomic RNA (gRNA) by SHAPE (selective 2’OH acylation analysed by primer extension) and dimerization assays.

Treatment of cells with NSC260594 did not reduce the number of integration events of incoming virus and treatment of virus producing cells did not affect the levels of intracellular Gag protein or viral particle release as determined by western blot. However, when levels of HIV-1 RNA packaged into virions were investigated, NSC260594 treatment resulted in up to 82% reduction in the amount of gRNA incorporated into virions, and a similar reduction in particle infectivity, without affecting intracellular levels of gRNA. SHAPE analyses revealed that NSC260594 had a stabilizing effect on the wild type RNA structure that was not confined to SL3 but was propagated across the extended RNA packaging signal (ψ) structure. This effect was specific to wild type HIV-1 RNA as it was not seen in a packaging mutant lacking SL3.

This study suggests that NSC260594 acts as a specific inhibitor of HIV-1 RNA packaging without affecting other viral functions. By binding to SL3, NSC260594 prevents its interaction with Gag but also stabilises not just SL3 but the structure of the wider ψ region. This confirms previously published data [1] using isolated SL3 RNA, that SL3 is structurally labile in the presence of Gag and that flexibility at this site is needed for the ψ region to be able to adopt different conformations. Since HIV-1 viral replication is otherwise unaffected by NSC260594 the flexibility of SL3 appears to be a prerequisite for HIV-1 RNA packaging and identifies this process as a novel, highly specific drug target. This study demonstrates that development of a new class of antiretroviral drugs targeting the viral packaging process by binding to the viral genomic RNA is achievable.

**Keywords:** HIV-1; Antiretroviral drugs; Packaging; RNA structure

**Reference**

1. Bell N.M., L’Hernault A., Murat P. et al., *Biochemistry* **52**, (2013) 9269–9274.

#### O29 LEDGIN treatment during virus production induces a latent HIV reservoir

##### Gerlinde Vansant, Lenard Vranckx, Irena Zurnic, Frauke Christ, Zeger Debyser

###### Molecular Virology and Gene Therapy, KU Leuven, Leuven, Belgium

**Correspondence:** Gerlinde Vansant

*Retrovirology* 2018, **15(Suppl 1)**:O29

Persistence of latent, replication-competent provirus is the main impediment towards a cure of HIV infection. Various strategies to eliminate the viral reservoir are being explored. We have recently proposed a novel block-and-lock strategy to reduce the functional HIV reservoir by LEDGIN-induced retargeting of HIV integration (Vranckx et al., 2016). LEDGINs are antivirals that inhibit the interaction between HIV integrase and the host factor LEDGF/p75 (Christ et al., 2010). LEDGIN treatment during infection inhibits integration (early effect), while addition of LEDGINs during virus production results in progeny virus with morphological and replication defects (late effect) (Christ et al., 2012; Desimmie et al., 2013; Jurado et al., 2013). We recently showed that LEDGIN treatment retargets integration of residual proviruses out of transcription units, rendering the virus more latent and resistant to reactivation (Vranckx et al., 2016). We now investigated the impact of LEDGIN treatment during virus production on integration site selection and latency. In these experiments, submicromolar concentrations of LEDGINs were added during virus production in 293T cells. Integration sites of progeny virus were determined by Illumina sequencing. To evaluate latency and reactivation, different cell lines were infected with a double-reporter single round virus and analyzed by flow cytometry.

LEDGIN treatment during virus production inhibited infection in a dose-dependent manner and resulted in a latent reservoir containing up to 95% of latent cells. This reservoir was refractory to reactivation. Integration sites of viruses produced in the presence of LEDGINs were shifted away from features associated with active transcription but enriched for markers linked to latency. In a multiple round infection experiment, human primary CD4+ T cells were infected with wild type NL4.3 virus in the presence of LEDGINs or raltegravir. Although both inhibitors reduced infection, only the residual provirus established under LEDGIN treatment, but not raltegravir, was hampered for reactivation.

In conclusion, LEDGIN treatment during production of virus resulted in a residual reservoir that was predominantly latent, similar to the phenotype previously observed for LEDGIN treatment during infection. Overall, these data show that LEDGIN treatment during acute infection might be an attractive approach to achieve an HIV remission by a block-and-lock strategy.

**Keywords:** HIV latency; LEDGF/p75; LEDGIN; HIV cure

### Session 8: HIV cure minisymposium

#### O30 Development of a novel method for HIV eradication: “lock-in and apoptosis”

##### Hiroshi Tateishi^1^, Halil Ibrahim Ciftci^1^, Ryoko Koga^1^, Kazuaki Monde^2^, Kensaku Anraku^3^, Kotaro Koiwai^4^, Fumiaki Yumoto^4^, Toshiya Senda^4^, Masami Otsuka^1^, Mikako Fujita^5^

###### ^1^Department of Bioorganic Medicinal Chemistry, Facylty of Life Sciences, Kumamoto University, Kumamoto 862-0973, Japan; ^2^Department of Microbiology, Faculty of Life Sciences, Kumamoto University, Kumamoto 860-8556, Japan; ^3^Department of Medical Technology, Kumamoto Health Science University, Kumamoto 861-5598, Japan; ^4^Structural Biology Research Center, Institute of Materials Structure Science, KEK/High Energy Accelerator Research Organization, Tsukuba, 305-0801, Japan; ^5^Research Institute for Drug Discovery, School of Pharmacy, Kumamoto University, Kumamoto 862-0973, Japan

**Correspondence:** Mikako Fujita

*Retrovirology* 2018, **15(Suppl 1)**:O30

Eradication of HIV reservoir from the body is a final goal of current AIDS study. For this purpose, “kick and kill” strategy has been tried, but “kill” device remains to be inefficient. Recently, we showed a clue to unprecedented strategy to HIV eradication named “lock-in and apoptosis”, in which efficient kill of HIV reservoir is expected [1] (Fig. 1).

**Fig. 1:**
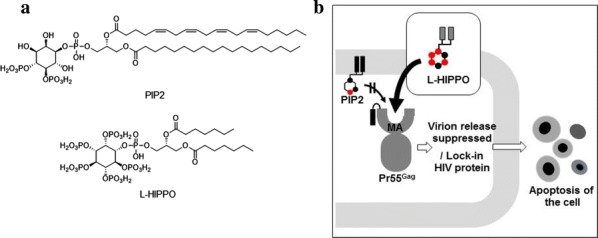
**a** Structures of PIP2 and L-HIPPO. **b** Principle of “lock-in and apoptosis” strategy

Inositol phospholipid PIP2 (Fig. 1a) was found to bind to MA domain of HIV-1 Pr55^Gag^ protein to be critical for virus release. We succeeded to synthesize an artificial compound named L-HIPPO (Fig. 1a) which binds to MA 70-fold stronger than that of PIP2 derivative [1]. Introduction of the L-HIPPO into a cell expressing HIV-1 proteins suppressed virus release, and finally induced apoptosis of the cell (Fig. 1b) probably by lock-in of HIV-1 proteins. This strategy together with latency reverting by agents should eradicate HIV.

To make this strategy amenable to clinical use, we are trying to elucidate the mechanism of apoptosis induction and improve this method. We have been investigating how L-HIPPO binds to MA using purified MA expressed in *E. coli*. Biophysical characterization of L-HIPPO and MA interaction and crystallization trial for X-ray structure analysis of the complex are in progress. In this symposium, we will introduce our new strategy and report the progress.

**Keywords:** HIV eradication; Apoptosis; Pr55^Gag^; MA; Inositol phospholipid

**Reference**

1. H. Tateishi, K. Monde, K. Anraku, R. Koga, Y. Hayashi, H.I. Ciftci, H. DeMirci, T. Higashi, K. Motoyama, H. Arima, M. Otsuka, M. Fujita, *Scientific Reports,* 7, **2017**, 8957.

#### O31 Heterogeneous HIV reactivation patterns of DSF and combined DSF + romidepsin treatments

##### Anna Kula^1,2^, Nadège Delacourt^1^, Sophie Bouchat^1*^, Gilles Darcis^1,3,4*^, Veronique Avettand-Fenoel^5,6^, Roxane Verdikt^1^, Francis Corazza^7^, Coca Necsoi^8^, Caroline Vanhulle^1^, Maryam Bendoumou^1^, Arsene Burny^1^, Stéphane De Wit^8^, Christine Rouzioux^5,6^, Olivier Rohr^9,10^ and Carine Van Lint^1^

###### ^1^Service of Molecular Virology, Département de Biologie Moléculaire (DBM), Université Libre de Bruxelles (ULB), Gosselies, Belgium; ^2^Malopolska Centre of Biotechnology, Jagiellonian University, Krakow, Poland; ^3^Laboratory of Experimental Virology, Department of Medical Microbiology, Academic Medical Center of the University of Amsterdam, Amsterdam, The Netherlands; ^4^Infectious Diseases Department, Liege University Hospital, Liege, Belgium; ^5^Service de Virologie, Université Paris-Descartes, AP-HP, Hopital Necker-Enfants Malades, Paris, France; ^6^Université Paris descartes, Sorbonne Paris Cité, EA 7327, Paris, France; ^7^Laboratory of Immunology, Brugmann University Hospital, Université Libre de Bruxelles (ULB), Bruxelles, Belgium; ^8^Service des Maladies Infectieuses, CHU St-Pierre, ULB, Bruxelles, Belgium; ^9^Institut de Parasitologie et de Pathologie Tropicale, EA7292, University of Strasbourg, Strasbourg, France; ^10^Institut Universitaire de Technologie Louis Pasteur de Schiltigheim, University of Strasbourg, Strasbourg, France

**Correspondence:** Anna Kula

*Retrovirology* 2018, **15(Suppl 1)**:O31

*Equal contribution

Few single latency reversing agents (LRAs) have been tested in vivo and only some of them have demonstrated an effect, albeit weak, on the decrease of latent reservoir. Therefore, other LRAs and combinations of LRAs need to be assessed to increase potency of latency reversal. Here, we evaluated the potential of combined treatments of therapeutically promising LRAs, disulfiram and romidepsin.

We assessed the reactivation potential of individual disulfiram or simultaneous or sequential combined treatments with romidepsin in vitro in latently-infected cell lines of T-lymphoid and myeloid origins and in ex vivo cultures of CD8+-depleted peripheral blood mononuclear cells (PBMCs) isolated from 18 HIV-1^+^ cART-treated individuals.

We demonstrated heterogeneous reactivation effects of disulfiram in vitro in various cell lines of myeloid origin and no latency reversal neither in vitro in T-lymphoid cells nor ex vivo, even if doses corresponding to maximal plasmatic concentration or higher were tested. Disulfiram + romidepsin combined treatments produced distinct reactivation patterns in vitro. *Ex vivo*, the combined treatments showed only a modest beneficial reactivation effect when used simultaneously as opposed to no viral reactivation for the corresponding sequential treatment.

Exclusive reactivation effects of disulfiram in myeloid latency cell lines suggest that disulfiram could be a potential LRA for this neglected reservoir. Moreover, distinct reactivation profiles pinpoint heterogeneity of the latent reservoir and confirm that the mechanisms that contribute to HIV latency are diverse. Importantly, disulfiram + romidepsin treatments are not potent ex vivo and most likely do not represent an effective drug combination to achieve high levels of latency reversal in vivo.

**Keywords:** HIV reactivation; Latency reversing agents; Disulfiram; Romidepsin; Heterogeneity of HIV latent reservoir

#### O32 Targeting HIV latency with metabolic cues

##### V Le Douce^1^, T McGinty^2^, JC Valle-Casuso^3^, SD Rezaei, S O’Reilly, A Macken^2^, W Tinago^2^, AM McCartin^1^, A Ait-Ammar^1,4^, O Rohr^4^, C Van Lint^5^, A Sáez-Cirión^3^, PW Mallon^2^, VW Gautier ^1^

###### ^1^UCD Centre for Research in Infectious Diseases, School of Medicine, University College Dublin, Ireland; ^2^HIV Molecular Research Group, Catherine McAuley Education and Research Centre, Mater Misericordiae University Hospital, Dublin, Ireland; ^3^Institut Pasteur, Unité HIV Inflammation et Persistance, Paris, France; ^4^University of Strasbourg, EA7292, FMTS, IUT Louis Pasteur, Schiltighem, France; ^5^Université Libre de Bruxelles, Service of Molecular Virology, Institute for Molecular Biology and Medicine, Gosselies, Belgium

**Correspondence:** V. W. Gautier

*Retrovirology* 2018, **15(Suppl 1)**:O32

Memory CD4+ T cells display unique quiescent metabolism that is intimately linked to their resting state. The tight relationship between metabolic pathways and HIV replication suggests that metabolic requirements for HIV reactivation are not met in HIV latent reservoirs.

In this context, we propose that targeted inhibition/activation of key metabolic junctions could adjust metabolism in a way that may be beneficial for HIV latency reversal. Here, we screened small drug molecules targeting OXPHOS, Glycolysis, FAO, FASN, mTOR, or AMPK in primary CD4+ T cells latently infected with HIV in vitro and examined their impact on HIV transcription. Drug treatments resulting in enhanced HIV gene expression were selected and tested in ex vivo HIV latency reactivation assays using CD8-depleted PBMCs isolated from HIV+ patients on suppressive cART for their capacity to trigger virion production. We present results of the primary and validation screens and focus on the drug ZAP1, which resulted in HIV production in ex vivo HIV reactivation assays from 11 out of 24 of HIV+ donors and worked in a synergistic manner when combined with Bryostatin in 5 out of 7 HIV+ donors. Of note, ZAP1 did not modulate expression of activation or proliferation markers (CD69, CD25, KI-67) on resting CD4+ T cells from healthy donors, but modulated their metabolic profiles.

Our results strongly suggest that metabolic drugs and Zap1 in particular could act as a new class of Latency Reversing Agents as part of new therapeutic approach for HIV latency reversal.

**Keywords:** HIV persistence; T cell metabolism; HIV latency reversal; Metabolic reprogramming

**Acknowledgements:** This project has received funding from the Irish Health Research Board and from the *European Union’s Horizon 2020 research and innovation programme* under grant agreement No 691119—EU4HIVCURE—H2020-MSCA-RISE-2015.

#### O33 The effect of Analytical Treatment Interruption on neuro inflammation, restriction factor expression and reservoir size in HIV infected individuals

##### M-A De Scheerder, C Van Hecke, S Rutsaert, L De Clercq, M Sips, L Vandekerckhove

###### HCRC, Ghent University Hospital, De Pintelaan 185, 9000 Gent, Belgium

*Retrovirology* 2018, **15(Suppl 1)**:O33

**Background**: Analytical treatment interruption (ATI) provide critical information about time to viral rebound (TTVR), but also, combined with extensive patients sampling, can give broader insights on the origin of viral rebound and can help identify potential biomarkers to predict viral rebound post-treatment interruption. Nevertheless, little is known about the safety of ATI and their long-term impact on patients’ health. We assessed the effect of ATI on the HIV reservoir, the expression of restriction factors and the impact on neuro-inflammation and neuronal injury. Furthermore we tried to identify correlations between TTVR, patient characteristics/virological and immunological parameters.

**Methods**: PBMCs, plasma and CSF were collected from 11 participants of the HIV-STAR study (NCT2641756) at different time points (Fig. 1). Digital droplet PCR was used to measure total HIV DNA. Neurofilament light (NFL) and YKL-40 protein were measured in CSF as markers of neuronal injury and neuroinflammation, respectively. In addition, neopterin, tryptophan and kynurenine were measured both in plasma and CSF as markers of immune activation. Quantitative real-time PCR was performed to determine the expression of known HIV-1 restriction factors (RF), cofactors and interferon stimulated genes (ISGs). Statistical Friedman’s, post hoc Dunn’s analysis and Spearman correlation were performed.

**Fig. 1:**
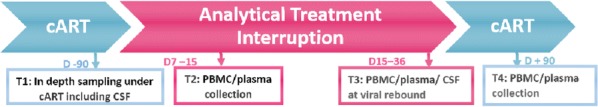
Sample flow HIV-STAR study

**Results**: No significant difference in total HIV DNA in PBMCs was observed between T1 and T4 (p = 0.2061). No significant increase in NFL or YKL-40 in CSF was observed between baseline and viral rebound (T1 versus T3). Furthermore, markers of immune activation did not increase during viral rebound. For three RF a significant or borderline significant difference in expression between T1 and T2 (APOBEC3G, SLFN11) and T1 and T3 (MX2) was observed. A significant difference in expression levels was defined between T1 and T3 for ISGs (IFIT1 and MX1). We did not identify significant correlations between expression of RF, participant characteristics (time since primary infection, time on cART, time before cART initiation), virological (total HIV-1 DNA in PBMCs, zenith viral load) and immunological parameters (CD4 nadir) and TTVR.

**Conclusion**: ATI did not increase total HIV load, nor did it reveal signs of increased neuronal injury or inflammation. RF increased in response to viral rebound, however their expression returned to baseline after treatment reinitiation. Overall, our data supports that ATI is safe and if combined with close monitoring and reinitiation of treatment, this intervention can be considered as the final and most comprehensive read out of HIV cure trials.

**Keywords:** Analytical treatment interruption; HIV Cure; Time to viral rebound; HIV reservoir

## Poster presentations

### TOPIC 1: Entry and uncoating

#### P1 Deciphering the early steps of HIV-1 infection by 3D super resolution microscopy

##### Elena I. Rensen^1^, Andrey Aristov^1^, Viviana Scoca^2^, Mickaël Lelek^1^, Pierre Charneau^2^, Christophe Zimmer^1^, Francesca Di Nunzio^2^

###### ^1^Imaging and Modeling Unit, Institute Pasteur, Paris, France; ^2^Molecular Virology and Vaccinology Unit, Institute Pasteur, Paris, France

**Correspondence:** Elena I. Rensen

*Retrovirology* 2018, **15(Suppl 1)**:P1

HIV-1 infection does not require nuclear envelope breakdown during mitosis. Thus, the passage of the viral preintegration complex (PIC) (~ 56 nm) through the nuclear pore complex (NPC) (inner channel ~ 39 nm diameter) is an obligated step for the virus to integrate its genome into the human host genome and replicate. The viral genome is enclosed in a ~ 100 nm conical shell composed of multimers of capsid (CA) protein, which serves as a reaction chamber for the reverse transcription of the RNA genome to DNA. It has been shown that several components of NPC participate in HIV-1 nuclear import and interact with HIV-1 capsid. The reverse transcription complex (RTC)/PIC must shed at least some of its CA, in a tightly regulated step known as uncoating, because the CA core is larger than the NPC channel width.

Studying uncoating using biochemical bulk assays does not provide information at the single cell level, whereas conventional light microscopy has a resolution of 200–300 nm, exceeding the size of the core, RTC/PIC and NPCs. Therefore, a consensus on the detailed timing and location of uncoating events, and on PIC interactions with NPCs, in successfully infectious virions containing the viral genome, is hitherto missing.

We implement an imaging approach to study the subcellular location and morphology of the viral genome, capsid shell and its interactions with NPC in single cells. We performed 2D and 3D super resolution localization microscopy in HIV-1 infected cells, using click chemistry to label reverse transcribed DNA, and immunostaining to label CA, integrase (IN) and nucleoporins. This technique enables us to analyze the subcellular fate of infectious viral particles, with a particular emphasis on the nuclear translocation and integration step.

We present preliminary results showing fluorescently labelled components of the NPC and labelled HIV-1 DNA, CA and IN in macrophage like cells, which are natural targets of HIV-1 in humans. Furthermore, we show morphological changes of these complexes relative to cytoplasmic and nuclear complexes highlighting the dynamics of vDNA on its way from the cytoplasm to its site of integration.

This approach allows for single virus imaging of the early steps of HIV-1 infection in the cytosol, at the nuclear pore complex and in the nucleus and thus might help to further understand the link between reverse transcription and uncoating, as well as nuclear import and integration in natural target cells.

**Keywords:** Super-resolution microscopy; Uncoating; Reverse transcription; Macrophages

#### P2 Superinfection interference and receptor usage of the newly emerging subgroup of avian leukosis virus

##### David Přikryl, Jiří Plachý, Filip Šenigl, Vít Karafiát, Anna Koslová, Dana Kučerová, Markéta Reinišová, Daniel Elleder, Jiří Hejnar

###### Department of Viral and Cellular Biology, Institute of Molecular Genetics, Prague, Czech Repubilc

**Correspondence:** David Přikryl

*Retrovirology* 2018, **15(Suppl 1)**:P2

Avian sarcoma and leukosis viruses (ASLV) have traditionally been classified based on the diversity of their envelope glycoproteins, antigenic cross-reactivity, superinfection interference, and host range into A to J subgroups. Specific host cell receptors explaining these virus specificities are assigned to subgroups A, B, C, D, E, and J. Recently, new ALV isolates emerging in South-East Asia and Japan cluster together as an independent subgroup with gp85 genetically distant to the classical ASLVs A-C [1].

We investigated this virus using recombinant reporter vector RCAS equipped with the env sequence of the prototype isolate JS11C1. The host range was very broad and similar to A subgroup, but we also observed some differences, e.g. the strong resistance of guineafowl embryo fibroblasts. We also showed the inability of the new virus to infect cells preinfected by subgroup A virus but not by viruses of other subgroups.

In support to our observations, we successfully conferred the susceptibility to the new ALVs by ectopic expression of Tva receptor in originally resistant mammalian cells. Vice versa, we abrogated the susceptibility of chicken DF-1 cells by CRISPR/Cas9-mediated knock-out of the *tva* receptor gene.

We also investigated pathogenicity of new ALVs by inoculating chicken eggs. All chickens infected by new ALVs died or were euthanized within two months after hatching because of extensive pathology (stunting, heart hypertrophy, atrophic hypoplastic bone marrow, …), while the control group infected by ALV subgroup A showed no significant pathology.

Taken together, we suggest that this new virus, designated ALV-K, represents a new ALV subgroup, which shares its entry receptor with ALV-A. Another example of such receptor sharing is Tvb, which enables entry of ALV subgroups B, D, and E.

**Keywords:** Retrovirology; Conference; Leuven; Belgium; Avian; Pathology; Receptor

**Reference**

1. Li et al. Isolation, identification and evolution analysis of a novel subgroup of avian leukosis virus isolated from a local Chinese yellow broiler in South China. Arch Virol. 161: 2717–25, 2016.

#### P3 Different susceptibility to sCD4 of acute and chronic HIV in a transmission cluster

##### Mélanie Bouvin-Pley^1^, Marie Leoz^2^, Emmanuelle Roch^1^, Alain Moreau^1^, Nicolas Bellini^1^, Olivia Blake^3^, Fabrizio Mammano^3^, Martine Braibant^1^, Jean-Christophe Plantier^2^, Denys Brand^1^

###### ^1^INSERM U1259, Université de Tours et CHRU de Tours, France; ^2^Normandie Université, UNIROUEN, EA2656 GRAM, Rouen, France; ^3^INSERM U941, Université Paris Diderot, Paris, France

**Correspondence:** Mélanie Bouvin-Pley

*Retrovirology* 2018, **15(Suppl 1)**:P3

HIV-1 transmission leads to a genetic bottleneck with a single or a few variants of the donor quasispecies establishing an infection in the new host. Our study aimed to better characterize this genetic bottleneck by comparing the phenotypic properties of envelope proteins from acute and chronic infections within the particular context of a HIV-1 transmission cluster.

Our transmission cluster involved four patients at the very early stage of infection and a potential chronically-infected donor. A set of 155 *env* sequences (22 to 31 full-length envelope genes per patient) was obtained by using Single Genome Amplification. Thirteen envelopes representative of the individual virus populations were selected to generate pseudoviruses. Their infectivity and susceptibility to entry inhibitors and to interferon were investigated.

The genotypic analyses confirmed that infection was likely established by a single variant in three patients and by two variants in the fourth case. However, the transmitted sequences harbored no evident common signature and they were scattered through various genetic lineages. The phenotypic analyses showed no difference in infectivity, CCR5 tropism, susceptibility to the CCR5 antagonist Maraviroc and to the fusion inhibitor Enfurvitide between acute and chronic viruses. Susceptibility to type-I interferon was also similar. The only property that distinguished transmitted viruses was their higher resistance to soluble CD4, as compared to variants from the chronically infected donor (p = 0.008; Mann–Whitney U test). Interestingly, the transmitted viruses also exhibited an enhanced sensitivity to occupation of the receptor CD4 by the anti-CD4 monoclonal antibody LM52. A significant inverse correlation was found between LM52 and sCD4 IC_50_ values (Spearman r = − 0.692, p = 0.011), suggesting that the Env glycoproteins from transmitted viruses bind less efficiently to CD4 than those of chronic viruses.

In conclusion, we observed the genetic bottleneck expected after HIV transmission with a limited number of variant identified in the four patients in acute infection. Interestingly, among the different genotypic and phenotypic properties explored here, only the susceptibility to sCD4 and to CD4-binding antibody distinguished transmitted viruses from the chronic isolates. Our data support the hypothesis that Transmitted/Founder viruses are unable to efficiently infect cells expressing low levels of CD4.

**Keywords:** HIV-1; T/F virus; Transmission cluster

#### P4 HIV-1 cell-to-cell transfer and dissemination in myeloid target cells are mediated by an envelope-dependent two-step cell fusion mechanism

##### Maorong Xie^1^, Lucie Bracq^1^, Brigitte Raynaud-Messina^2^, Camille Ciccone^1^, Maeva Dupont^2^, Isabelle Maridooneau-Parini^2^, Christel Verollet^2^, Jerome Bouchet^1^and Serge Benichou^1^

###### ^1^Institut Cochin, Inserm U1016, CNRS UMR8104, Université Paris-Descartes, Paris, France; ^2^Institut de Pharmacologie et de Biologie Structurale, Université de Toulouse, CNRS, Université Paul Sabatier, Toulouse, France

**Correspondence:** Maorong Xie

*Retrovirology* 2018, **15(Suppl 1)**:P4

Dendritic cells (DCs), macrophages as well as osteoclasts (OCs) are emerging as target cells of HIV-1 involved in (i) sexual transmission, (ii) virus dissemination in different tissues, and (iii) establishment of persistent virus reservoirs. While the mechanisms of virus cell-to-cell transmission toward these myeloid cells remain poorly understood, we recently reported a novel very efficient mechanism for virus transfer and dissemination in macrophages trough a two-step cell fusion process, leading to the formation of long-lived and highly HIV-1-productive multinucleated giant cells (MGCs) [1]. Here, we show that HIV-1 uses similar cell-fusion mechanisms for virus transfer from infected T lymphocytes to OCs and immature DCs (iDCs) and subsequent dissemination in these target cells. The establishment of contacts with infected T cells leads to heterotypic cell fusion for the fast and massive transfer of viral material in OC and iDC targets. This process subsequently triggers homotypic fusion with non-infected neighboring OCs and iDCs for intercellular virus dissemination. Both cell fusion steps are mediated by viral envelope-receptor interactions, and are highly efficient for macrophage-tropic CCR5- and CXCR4-using viruses, dual-tropic R5X4 viruses, and to a lesser extent for non-macrophage-tropic R5 viruses. Interestingly, these cell-to-cell fusion processes result in the formation of infected MGCs able to produce fully infectious virus particles. Together, our results reveal original mechanisms for viral transfer and dissemination in HIV-1 myeloid cell targets and for the formation of the MGCs observed in vivo in lymphoid and non-lymphoid tissues of HIV-1-infected patients. They contribute to a better understanding of the cellular processes involved in virus transmission, dissemination, and formation of viral reservoir during HIV-1 infection. 
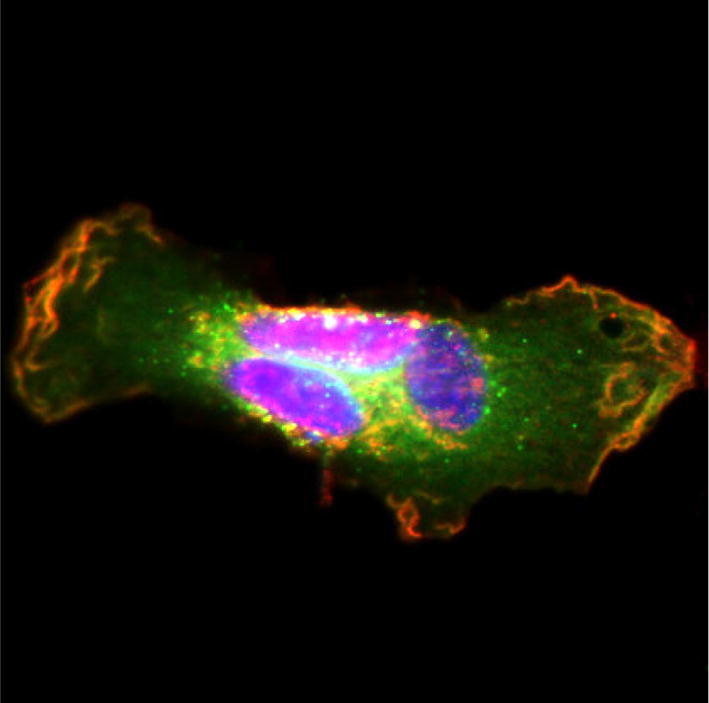


T cell-dendritic cell fusion induces multinucleated giant cell (MGC) formation. DC-SIGN (red), HIV-1 Gag (green), T cell nuclei (magenta), DRAQ5 (blue).

**Keywords:** HIV-1 cell-to-cell transfer; Cell fusion; CD4+ T cells; Myeloid cells

**Reference**

1. Bracq, L., Xie, M., Lambele, M., Vu, L.T., Matz, J., Schmitt, A., Delon, J., Zhou, P., Randriamampita, C., Bouchet, J., *Journal of Virology*, vol. 91 no. 24 **2017**, 01237–17.

### TOPIC 2: RT and integration

#### P5 Cargo binding of LEDGF/p75 is dependent on DNA interaction

##### Tine Brouns^1,2^, Herlinde De Keersmaecker^2^, Noriyuki Kodera^4^, Toshio Ando^4^, Willem Vanderlinden^2,3^, Jan De Rijck^1^, Steven De Feyter^2^, Zeger Debyser^1^

###### ^1^Molecular Virology and Gene Therapy, KU Leuven, Leuven, Belgium; ^2^Division of Molecular Imaging and Photonics, KU Leuven, Leuven, Belgium; ^3^Department of Physics, Nanosystems Initiative Munich, and Center for NanoScience, LMU Munich, Munich, Germany; ^4^Nano-Life Science Institute, Kanazawa University, Kakuma-machi, Kanazawa, Japan

**Correspondence:** Tine Brouns

*Retrovirology* 2018, **15(Suppl 1)**:P5

Lens epithelium-derived growth factor p75 (LEDGF/p75) is a DNA binding transcriptional co-activator that interacts with multiple cargoes through the integrase binding domain (IBD). In the HIV replication cycle, it interacts with HIV integrase (IN) to guide the complex to a specific chromatin environment. LEDGF/p75 engages the chromatin with two distinct functional interfaces: (1) the N-terminal PWWP domain interacts specifically with methylated histon 3 (H3K36me3) and (2) the adjacent basic surface, binds DNA non-specifically [1]. This interface is composed of the nuclear localization signal and two AT-hook motifs. Previous studies with charge reversal mutations in the NLS and AT-hook-like domains of LEDGF/p75 (R147D, R149D, R183D, K192D, and R195D, LEDGF/p75^mut^), showed that despite their lack of DNA binding activity, these mutants retained the ability to efficiently stimulate HIV-1 integrase activity in vivo [2].

In our AlphaScreen assays, we found that the binding of LEDGF/p75^mut^ to HIV IN is comparable to the wild type LEDGF/p75. In contrast, the interaction of this mutant with cellular binding partners of LEDGF/p75, e.g. JPO2 and MLL, was completely disrupted. A similar effect was observed upon addition of nucleases to the assay, clearly indicating that the non-specific DNA binding significantly affects the interaction with the cellular partners, but not with HIV IN. To study this effect in molecular detail, the structural parameters of both proteins were investigated with high-speed AFM. This technique was used before to reveal that LEDGF/p75 can bind to DNA in a torque-dependent invasive mode, introducing flexible bends [3]. In this study we show that the LEDGF/p75^mut^ does not introduce similar bending. We postulate that LEDGF/p75-induced DNA bending allosterically regulates cargo binding at the IBD. Potentially, such a mechanism may trigger LEDGF/p75 functional tethering in the nucleus.

**Keywords:** LEDGF/p75; DNA; AFM; Protein–protein interactions

**References**

1. Eidahl J.O. *et. al.*, Structural basis for high-affinity binding of LEDGF PWWP to mononucleosomes. *Nucleic Acids Res.* 41 **2013**, 3924–3936.
2. Turlure F. *et. al.*, A tripartite DNA-binding element, comprised of the nuclear localization signal and two AT-hook motifs, mediates the association of LEDGF/p75 with chromatin in vivo. *Nucleic Acids Res.* 34 **2006**, 1653–1675.
3. Vanderlinden W. *et. al.,* Structure, mechanics, and binding mode heterogeneity of LEDGF/p75-DNA nucleoprotein complexes revealed by scanning force microscopy. *Nanoscale* 6 **2014**, 4611–4619.

#### P6 Structure-based mutagenesis of the lentiviral intasome

##### Vidya Chivukula, Allison Ballandras-Colas, Peter Cherepanov

###### Chromatin Structure and Mobile DNA Laboratory, The Francis Crick Institute, London, UK

**Correspondence:** Vidya Chivukula

*Retrovirology* 2018, **15(Suppl 1)**:P6

Integration of viral DNA (vDNA) into a host chromosome, by action of the viral enzyme integrase (IN), is an essential step of the retroviral lifecycle. To fulfil its function, IN assembles into a multimer on the vDNA ends, forming a highly stable nucleoprotein complex known as the intasome. The intasome architecture varies between the retroviral genera, and the maedi-visna virus (MVV) IN forms the largest intasome assembly observed to date, comprising a homo-hexadecamer. The conserved intasome core (CIC), found in all structurally characterized intasomes, is formed between a pair of MVV IN tetramers, each providing an active site, and is completed by the insertions of the synaptic C-terminal domains (CTDs) from the flanking tetramers. It was argued that this configuration is necessitated by the propensity of MVV INto form tetramers in solution and the alpha-helical structure of the MVV IN catalytic core domain (CCD)-CTD linkers. Furthermore, within the MVV IN hexadecamer, a pair of CTD tetrads bridge the IN tetramers by forming intra and inter tetramer interactions. Using site directed mutagenesis, we are probing the importance of these distinctive structural features. We show that the mutations disrupting the MVV CTD–CTD interfaces perturb the ability of MVV INto form multimers and assemble into stable intasomes and strongly affect its strand transfer activity in vitro. Likewise, mutations corrupting the alpha-helical configuration of the CCD-CTD linkers are highly disruptive to MVV IN strand transfer activity. Collectively, our observations indicate that the hexadecameric architecture is critical for the MVV IN function.

**Keywords:** Lentivirus; Integration; Intasome assembly; Conserved intasome core

#### P7 Structures-Functions relationship in HIV-1 pre-integration complexes

##### Julien Batisse^1^, Eduardo Bruch^1^, Nicolas Levy^1^, Benoit Maillot^1^, Sylvia Eiler^1^, Oyindamola Oladosu^1^, Karine Pradeau^1^, Corinne Crucifix^1^, Julio Ortiz Espinoza^1^, Patrick Schultz^1^, Bruno Kieffer^1^, Serge Bouaziz^2^, Olivier Delelis^3^, Vincent Parissi^4^, Marc Ruff^1^

###### ^1^IGBMC, Department of Integrated Structural Biology, UMR7104 (CNRS, INSERM, UDS), Illkirch, France; ^2^Université Paris Descartes, Sorbonne Paris Cité, Paris, France; ^3^Université Paris-Saclay, Cachan, France; ^4^Fundamental Microbiology and Pathogenicity Laboratory, University of Bordeaux, Bordeaux Cedex, France

**Correspondence:** Marc Ruff

*Retrovirology* 2018, **15(Suppl 1)**:P7

After retroviral infection of a target cell, during the early phase of replication, the HIV-1 genomic RNA is reverse transcribed by the viral reverse transcriptase to generate the double-stranded viral DNA that interact with viral and cellular proteins to form the pre-integration complex (PIC). Viral integrase (IN) is the key component of the PIC and is involved in several steps of replication notably in reverse transcription, nuclear import, chromatin targeting and integration. Viral components such as IN cannot perform these functions on their own and need to recruit host cell proteins to efficiently carry out the different processes. IN is a flexible protein, property allowing its interaction with multiple partners and enabling its multiple functions in viral replication. To study the molecular mechanisms of viral integration we use a bottom–up strategy by assembling in vitro and/or *in cellulo* multiprotein complexes around the integrase protein (core protein of the PIC) and DNA. Several complexes have been characterized in our team (IN/LEDGF, IN/LEDGF/INI1-IBD, IN/LEDGF/CA, IN/LEDGF/TNPO3, IN/VBP1/TNPO3, IN/LEDGF/Nucleosome). Two cryo-EM structures of the IN/LEDGF/DNA and IN/LEDGF/INI1-IBD/DNA complexes have been solved at low resolution [1, 2]. With the recent progress of the cryo-EM techniques and our improvement in the complexes preparations [3], new cryo-EM datasets are collected (IN/LEDGF/DNA-pal, IN/LEDGF/Nucleosome) which will enable us to increase the structure quality to near atomic resolution. To reveal the structure–function relationships of PIC complexes, we combine X-ray, NMR and Cryo-EM structures with biochemical and biological data. The latest results obtained will be presented at the meeting.

**Keywords:** HIV; Structural Biology; Pre-integration complexes; Cryo-EM

**References**

1. Michel et al., EMBO J., 28, 2009, 980–991
2. Maillot et al., PLoS ONE 8(4), 2013, e60734
3. Levy et al., Nature comm. 7, 2016, 10932

#### P8 Real-time fluorescence lifetime imaging of HIV-1 nuclear import

##### Nagma Parveen^1^, Irena Zurnic^2^, Johan Hofkens^1^, Zeger Debyser^2^, Jelle Hendrix^1, 3^

###### ^1^Laboratory for Photochemistry and Spectroscopy, Molecular Imaging and Photonics Division, Chemistry Department, KU Leuven, Leuven, Belgium; ^2^Laboratory for Molecular Virology and Gene Therapy, Department of Pharmaceutical and Pharmacological sciences, KU Leuven, Leuven, Belgium; ^3^Dynamic Bioimaging Lab, Advanced Optical Microscopy Centre and Biomedical Research Institute (BIOMED), Hasselt University, Diepenbeek, Belgium

**Correspondence:** Nagma Parveen

*Retrovirology* 2018, **15(Suppl 1)**:P8

Nuclear import of the HIV-1 pre–integration complex (PIC), the large nucleoprotein complex composed of double–stranded vDNA, cellular and viral proteins, is a critical yet ill-understood step in viral replication. The importance in unravelling the mechanistic details of the process is in developing anti-virals targeting different components of PIC, and also bioengineering retroviruses for gene therapy. Virology assays such as western blot have provided a great deal of information about the molecular processes involve in the nuclear import of PIC. However, the responses are ensemble-averaged and may not necessarily corresponds to the actual infectious complexes. Fluorescence microscopy techniques provide a platform to detect single PICs in infected live cells and even quantify the underlying molecular interactions using suitable analysis methods.

With the aim to shed more light on the nuclear import of HIV-1 PIC we imaged integrase (IN), a retroviral enzyme that catalyzes vDNA insertion into the host genome. We focused on examining the quaternary structural changes of IN during and upon the nuclear import, phenomenon reported to be crucial for the integration of HIV-1 PIC. For this, we used a Fröster resonance energy transfer (FRET) assay employing fluorescence lifetime imaging microscopy (FLIM) technique. The assay was implemented via imaging the fluorescence lifetime of fluorescently-labeled IN of single HIV-1 PICs in real-time. The changes in the fluorescence lifetime of a single PIC over its trajectory allows to analyze the real-time structural changes of HIV-1 IN.

**Figure:**
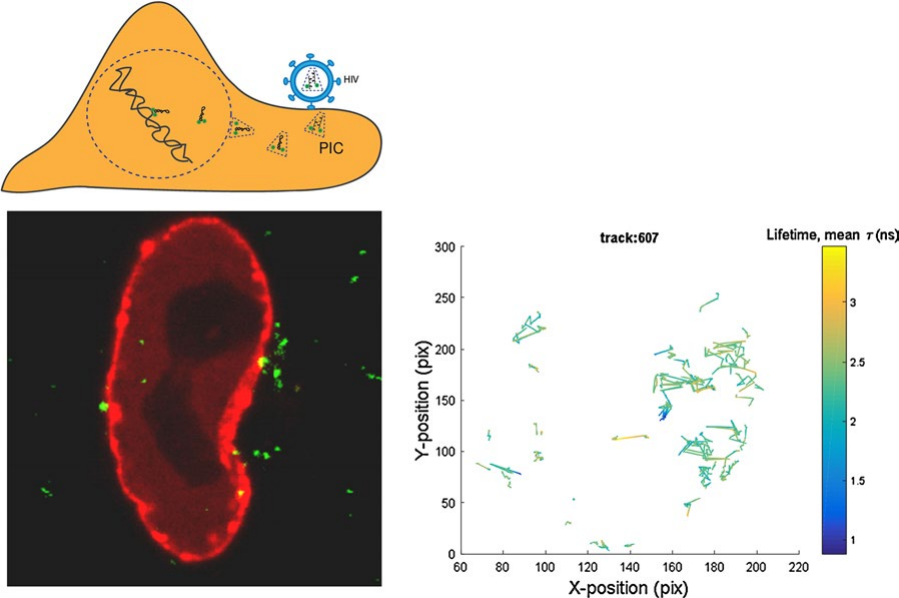
**Figure 1**. Scheme to illustrate the transport of HIV-1 PIC upon infection. A confocal image of a cell infected with HIV-1, where the green and red signal are from the fluorescently labeled IN of HIV-1 and fluorescently-labeled nuclear lamin, respectively. The graph on the right shows the tracks of single HIV-1 PICs with a colormap of their corresponding fluorescence lifetime

**Keywords:** HIV-1; FRET; Real-time imaging; Single particle tracking

#### P9 Visualisation of the Human Immunodeficiency Virus type 1 cDNA by click chemistry

##### Flore De Wit^1^, Akkaledevi Venkatesham^2^, Sambasiva Rao Pillalamarri^2^, Arthur Van Aerschot^2^, Zeger Debyser^1^

###### ^1^Molecular Virology and Gene Therapy, KU Leuven, Leuven, Belgium; ^2^Medicinal Chemistry, Rega Insitute for Medical Research, KU Leuven, Leuven, Belgium

*Retrovirology* 2018, **15(Suppl 1)**:P9

One of the key steps in the human immunodeficiency virus type 1 (HIV-1) replication cycle is the reverse transcription of viral RNA into a double-stranded copy DNA (cDNA), by the viral reverse transcriptase (RT). This cDNA is subsequently transported in the form of a pre-integration complex (PIC) to the nucleus where it is finally integrated into the host chromosome by the viral integrase (IN). Although much is known about the biochemistry and inhibition of reverse transcription, many questions remain about its timing and location within the cellular environment.

A recently developed single virus imaging technique enables us to study labelled IN (IN-eGFP) inside individual viral complexes during the early stages of the replication cycle [1]. Different parameters including the number of fluorescent viral complexes, their distance to the nuclear envelope and intensity can be determined from these experiments. Although we can now identify single viral complexes in infected cells, we are unable to identify whether they contain reverse transcribed DNA. For this purpose, we combined our existing assay with the labelling of viral DNA using click chemistry. Ethynyl-functionalised nucleosides can be incorporated by RT and allow covalent linkage with azide reactive fluorophores via a copper-catalyzed azide-alkyne cycloaddition. In 2014, Peng and colleagues showed that it is possible to visualize HIV cDNA using 5-ethynyl-2’-deoxyuridine (EdU) [2]. However, this technique has some drawbacks. There is a high off-target labelling of the nucleus and cytoplasm due to EdU incorporation by the host DNA polymerases. Therefore, this technique is limited to the use in non-diving cells such as monocyte-derived macrophages (MDM) [3].

To this end, we opt to develop RT specific ethynyl-functionalised nucleosides that are not incorporated by the cellular DNA polymerases. With these analogues we could already lower the off-target labelling of the cellular DNA and reached up to 2% co-localisation of the total number of IN-eGFP with the viral DNA staining in HeLa P4. The RT specificity was already shown in an in vitro primer extension assay. Further validation of the analogue specificity will be done with a RT catalytic inactive mutant.

**Keywords:** HIV; DNA labeling; Click chemistry; Imaging

**References**

1. Borrenberghs, D. et al. Sci. Rep. 6, 1–14 (2016).
2. Peng, K., Muranyi, W., Glass, B., Laketa, V. & Yant, S. R. Elife 3, e04114 (2014).
3. Stultz, R. D., Cenker, J. J. & Mcdonald, D. J. Virol. 91, e00034-17 (2017).

#### P10 Flexible but conserved: a new essential motif in the CTD of HIV-1 group M integrases

##### M Kanja^1^, P Cappy^1^, G Blanco-Rodriguez^2^, N Levy^3^, O Oladosu^3^, S Schmidt^4^, P Rossolillo^1^, R Gasser^1^, C Moog^4^, M Ruff^3^, F Di Nunzio^2^, M Negroni^1^, D Lener^1^

###### ^1^Retroviruses and Molecular Evolution, Architecture et Réactivité de l’ARN, Strasbourg University, Strasbourg, France; ^2^Molecular Virology and Vaccinology, Institut Pasteur, Paris, France; ^3^Chromatin Stability and DNA mobility, Department of Structural Biology and Genomic, Strasbourg University, Illkirch, France; ^4^Molecular Immuno-Rhumatology Laboratory, Université de Strasbourg, Strasbourg, France

**Correspondence:** D. Lener

*Retrovirology* 2018, **15(Suppl 1)**:P10

Several independent zoonotic transmissions have generated the different phylogenetic groups of HIV-1. Not all the groups have however undergone the same epidemiological success. Namely, HIV-1 group M is the one responsible for the pandemic, and group O is the second most abundant HIV, although with a largely lower epidemiologic success. The reasons for this discrepancy are only partially known to date.

HIV integrase (IN) catalyses the integration of the reverse transcribed viral DNA into the cell genome and has a central role in the control of the integration sites influencing the balance between latency of infection and viral replication. Being also involved in other steps of the infectious cycle, as reverse transcription, maturation of the viral particle and incorporation of the genomic RNA inside the viral core, the IN constitutes a keystone to the success of viral adaptation to new hosts.

Exploiting the natural genetic diversity existing among primary isolates of HIV-1 groups M and O, we have generated chimeras between IN of isolates of these groups and characterized their functionality. We have thus identified, in the C terminal domain of the enzyme, a new functional motif constituted of two lysines and two asparagines (NKNK motif). The NKNK motif is specific of group M isolates and is essential for integration. Indeed, the absence of lysines or asparagines results in decreased reverse transcription, marked reduction of nuclear import of reverse transcription products and reduced catalytic activity. A remarkable feature of the NKNK motif is its biochemical flexibility. Indeed, despite its strict conservation in vivo, the positions of the residues can be swapped in cell culture, often not affecting the integration process *per se*. This observation suggests that the motif could provide a surface of interaction with a partner yet to identify. Interestingly, though, the motif was not flexible with respect to reverse transcription, which was optimal exclusively with the canonical NKNK motif.

The biochemical versatility of this region of the integrase to carry out integration could have provided a major asset during viral evolution for acquiring additional functions during the infectious cycle, as its implication in reverse transcription.

**Keywords:** Integrase; Phylogenetic groups; Reverse transcription; Nuclear import

#### P11 The HIV-1 integrase interaction with Ku70 in the postintegrational gap repair

##### Marina Gottikh^1^, Andrey Anisenko^2^, Ekaterina Knyazhanskaya^3^, Dmitriy Mazurov^4,5^

###### ^1^Belozersky Institute of Physical and Chemical Biology, Lomonosov Moscow State University, Moscow, Russia; ^2^Faculty of Bioengineering and Bioinformatics, Lomonosov Moscow State University, Moscow, Russia; ^3^Chemical Department, Lomonosov Moscow State University, Moscow, Russia; ^4^NRC Institute of Immunology FMBA of Russia, Moscow, Russia; ^5^Cell and Gene Technology Group, Institute of Gene Biology RAS, Moscow, Russia

**Correspondence:** Marina Gottikh

*Retrovirology* 2018, **15(Suppl 1)**:P11

The integration of the lentiviral DNA into the host genome is completed by cellular factors that repair the short gaps flanking the proviral DNA. Several repair complexes have been implicated to participate in the repair of the integration intermediate including components of the non-homologous DNA repair pathway (NHEJ) [1]. NHEJ is generally initiated by the binding of Ku heterodimer to a double strand DNA break. However, no DSBs are formed during lentiviral integration. We have shown earlier, that HIV-1 integrase can directly interact with Ku70 repair factor, and this interaction is weakened by E212A/L213A substitutions in integrase [2]. Overexpression of an integrase-binding domain of Ku70 in cells reduces the transduction by a single round replication incompetent CMV-driven HIV-1 vector but has no influence on the vector bearing the E212A/L213A substitutions. This effect may be caused by an inhibition of the interaction between integrase and the endogenous Ku70 protein by an integrase-interacting Ku70 domain. Using CRISPR/Cas9 technology we have established a set of 293T derived sublines with a stable depletion of either Ku70, Ku80 or DNA-PKcs subunits of the DSB detection complex. The depletion of any subunit resulted in a decrease in HIV-1 single cycle replication which be caused by a compromised integration or by a reduction in the postintegrational gap repair. To choose between these two possibilities, we have established a qPCR-based approach for a quantitative measurement of postintegrational gap repair. We have shown that depletion of any of the DNA-PK components reduced gap repair efficiency in our system. The same effect has been detected in the presence of specific DNA-PKcs inhibitor Nu7441 on 293T cells as well as on the lymphoid Jurkat cells. Viral vector carrying E212A/L213A integrase substitutions demonstrates decreased gap repair and a reduced sensitivity to the DNA-PK components depletion while its integrational capacity remains at the wild type level. We speculate that integrase recruits DNA-PK complex to gaps and facilitates gap repair through a direct interaction with Ku70 subunit.

This work has been supported by an RSF grant 17-14-1107 (preparation of knocked down cell lines and qPCR-based approach) and by an RFBR grant 18-34-0039.

**Keywords:** HIV-1; Integrase; Postintegrational repair; Ku

**References**

1. Knyazhanskaya E.S., Shadrina O.A., Anisenko A.A., Gottikh M.B. *Molecular biology*. V. 50 (4) **2016**, 567–579.
2. Anisenko A., Knyazhanskaya E. et al.*Scientific reports*. V. 7(1) **2017**, 5649.

#### P12 tRNA-assisted maturation of HIV-1 reverse transcriptase

##### Tatiana V. Ilina^1^, Ryan L. Slack^1^, Stefan G. Sarafianos^2^, Rieko Ishima^1^

###### ^1^Department of Structural Biology, University of Pittsburgh School of Medicine, Pittsburgh, PA, USA; ^2^Department of Pediatrics, Emory University School of Medicine, Atlanta, GA, USA

*Retrovirology* 2018, **15(Suppl 1)**:P12

HIV-1 reverse transcriptase (RT) is a heterodimer consisting of two subunits p66 and p51, where subunit p51 is a product of HIV-1 Protease-catalyzed cleavage of the RNase H domain located at the C-terminus of p66. The processing of the p66/p66 homodimer to the mature p66/p51 heterodimer is a critical step in virus maturation and reverse transcription, and is essential for enabling infectivity. The proteolytic cleavage site in p66 subunit is sequestered in the middle of a β-sheet and inaccessible to the protease in the known p66/p51 RT structures. The molecular mechanism of how the mature p66/p51 heterodimer is formed is unknown. Here we report that the proteolytic processing of the RT p66/p66 homodimer to the mature p66/p51 heterodimer is significantly facilitated by interaction of the homodimer with tRNA. Other RNAs molecules have considerably less pronounced effect. Based on our biochemical and structural data, we propose a model in which interaction of the p66/p66 homodimer with tRNA introduces conformational asymmetry in the two subunits, permitting specific proteolytic processing of one of the two p66 subunits in p66/p66 to form the p66/p51 heterodimer.

**Keywords:** HIV-1 reverse transcriptase, p66, maturation, tRNA

**Acknowledgements:** This research was supported by NIH (R01 GM105401 and R01 AI100890).

#### P13 Studies on HIV Reverse Transcriptase inhibitory activity of isatin derivatives

##### Periyasamy Selvam^1^, RajKamal Tripathi^2^

###### ^1^Cherran College of Pharmacy, Coimbatore, TN India, India; ^2^Immunotoxicology Lab, Central Drug Research Institute (CDRI), Lucknow, India

**Correspondence:** Periyasamy Selvam

*Retrovirology* 2018, **15(Suppl 1)**:P13

**Background**: HIV Reverse Transcriptase (HIV RT) plays important roles in HIV virus replication, including conversion of viral RNA into cDNA. Identification of novel inhibitors of HIV Reverse Transcriptase has emerged as potential antiviral agents for the treatment of HIV/AIDS. Present work is to investigation of HIV Reverse Transcriptase inhibitory activity of isatin derivatives (2,3-dioxoindole) and its fused compounds

**Method**: Isatins and its condensed compounds indeno and Indolo (fused isatin compounds) were investigated for inhibition of HIV Reverse Transcriptase enzymatic activity to understand the mechanism of antiviral action.

**Results**: All compounds exhibited inhibitory activity against HIV-1 Reverse Transcriptase (25–95% inhibition at 100 μg/mL). The 5-bromo isatin and 5-chloro isatin displayed significant inhibitory activity against HIV RT enzymatic activity (66 and 95 at 100 µg/mL), respectively, whereas standard Nevirapine was found to be 99% activity at 10 µg/mL. The fused isatin derivatives such as Indolo and Indeno derivatives less active than isatin derivatives.

**Conclusion**: All the isatin derivaties inhibit the HIV Reverse Transcriptase (HIV RT) activity and 5-chloroisatin had significant activity against HIV 1 Reverse Transcriptase and ring fusion in isatin molecules leads to loss the HIV RT activity

**Keywords:** HIV 1; HIV RT; Isatin

**Reference**

1. Selvam P, Murugesh N, Chandramohan M, Debyser Z, Witvrouw M.Design, Synthesis and antiHIV activity of Novel Isatine-Sulphonamides.*Indian J Pharm Sci.* **2008** Nov;70(6):779–82

**Table:** **Table 1**. *In vitro* Anti-HIV-RT activity

Sample ID	% Inhibition at 10 µg/ml	% Inhibition at 100 µg/ml
Isatin	16.49	46.36
5-Chloro Isatin	39.10	95.09
5-Bromo Isatin	30.42	66.09
5-Chloro Indolo	17.83	25.90
Indeno	22.07	41.22
Nevirapine	99.61	

#### P14 Investigation of anti-HIV RT activity and anti-HIV activity of Poly Herbal Powder BH

##### Periyasamy Selvam^1^, S Balraj^2^, RajKamal Tripathi^3^, Dr Deepshikha^4^

###### ^1^Cherran College of Pharmacy, Coimbatore, TN India, India; ^2^Rovers Drugs, Perambalur, Tamilnadu, India; ^3^Immunotoxicology Lab, Central Drug Research Institute, Lucknow, India; ^4^Dept of Biochemistry, AIIMS, New Delhi, India

**Correspondence:** Periyasamy Selvam

*Retrovirology* 2018, **15(Suppl 1)**:P14

**Background**: HIV Reverse Transcriptase (HIV RT) plays important roles in several steps of HIV virus replication, including conversion of viral RNA into cDNA. Identification of novel inhibitors of HIV Reverse Transcriptase has emerged as promising antiviral agents for the treatment of HIV/AIDS. Present work is to investigation of anti-HIV activity and HIV Reverse Transcriptase inhibitory activity of various extracts of polyherbal powder (BH).

**Method**: Ethanolic extract of Polyherbal powder (BH-ET) were tested for anti-HIV activity against HIV-1(NL 4.3) replication in 5000 TZM B1 cells. BH extracts were investigated for inhibition of HIV Reverse Transcriptase enzymatic activity to understand the mechanism of antiviral action.

**Results**: All extracts exhibited inhibitory activity against HIV-1 Reverse Transcriptase (28–71% inhibition at 100 μg/mL). The ethanolic extract (BH-HT) and Hexane extract (BH-H) displayed significant inhibitory activity against HIV RT in enzymatic activity (63 and 71 at 100 µg/mL), respectively, whereas standard Nevirapine was found to be 99& at 100 µg/mL. The ethanolic extract also had 60% inhibition of the HIV-1 replication at the concentration of 100 µg/mL and standard AZT had 100% inhibition at 5 µM.

**Conclusion**: All the extracts inhibit the HIV Reverse Transcriptase (HIV RT) activity and ethanolic extract (BH ET) inhibit both HIV 1 replication and Reverse Transcriptase.

**Keywords:** HIV 1; HIV RT; Poly herbal Powder (BH); TEM B1 cells

**Reference**

1. Balraj S., Selvam P., Pommier Y., Metifiot M., Christophe M., Pannecouque C., and De Clercq E. Investigation of anti-HIV activity, cytotoxicity and HIV integrase inhibitory activity of polyherbal formulation BH extracts. *BMC Infect Dis.* **2014**; 14(Suppl 3): O23

**Table:** **Table 1**. *In vitro* Anti-HIV-RT activity

S. no.	Compound code	% Inhibition at 10 µg/ml	% Inhibition at 100 µg/ml
1.	BH-A	6.83	28.23
2.	BH-CH	26.69	30.10
3.	BH-Me	21.81	30.58
4.	BH-ET	61.51	63.48
5.	BH-H	52.61	71.74
6.	Nevirapine	99.45	

#### P15 Pinpointing recurrent proviral integration sites in new models for HIV-1 infection

##### Ulrike C. Lange^1,2,3^, Julia K. Bialek^1,2,§^, Thomas Walther^1,§^, Joachim Hauber^1^

###### ^1^Heinrich Pette Institute, Leibniz Institute for Experimental Virology, 20251 Hamburg, Germany; ^2^Department of Anesthesiology, University Medical Center Hamburg-Eppendorf, Hamburg, Germany; ^3^Center for Infection Research (DZIF), partner site Hamburg, Germany

**Correspondence:** Ulrike C. Lange

*Retrovirology* 2018, **15(Suppl 1)**:P15

HIV infection is characterized by accumulation of proviral sequences within the human host genome. Integration of viral-derived DNA occurs at preferential loci, suggesting a site-specific crosstalk between viral sequences and human genes. We here describe a genome engineering workflow to generate models for HIV-1 infection that for the first time recapitulate proviral integration at selected genomic loci and provide unique tools to study effects of HIV proviral integration site choice. Using this workflow, we have derived BACH2–HIV-1 reporter models that mimic largely latent integration in the clinically relevant BACH2 gene locus, which has been associated with recurrent integration and HIV-reservoir maintenance in chronically infected patients.

**Keywords:** HIV integration; HIV model systems; HIV latency; Genome engineering; CRISPR/Cas9

**Reference**

1. Lange UC, Bialek JK, Walther T, Hauber J, *Virus Research*, 249 **2018,** 69–75.

#### P16 Affinity switching of the LEDGF/p75 IBD interactome is governed by kinase-dependent phosphorylation

##### Subhalakshmi Sharma^1^, Katerina Cermáková^2,3,4,5^, Jan De Rijck^1^, Jonas Demeulemeestera^1^, Milan Fábryf^6^, Sara El Ashkar^1^, Siska Van Belle^1^, Martin Lepsík^2^, Petr Tesinab^6^, Vojtech Duchoslav^2^, Petr Novák^7^, Martin Hubálek^2^, Pavel Srb^2^, Frauke Christ^1^, Pavlína Rezácová^2,6^, H. Courtney Hodges^3,4,5,8^, Zeger Debyser^1^, and Václav Veverkab^9^

###### ^1^Molecular Virology and Gene Therapy, KU Leuven, 3000 Leuven, Belgium; ^2^Institute of Organic Chemistry and Biochemistry of the Czech Academy of Sciences, 166 10 Prague 6, Czech Republic; ^3^Department of Molecular & Cellular Biology, Baylor College of Medicine, Houston, TX 77030; ^4^Center for Precision Environmental Health, Baylor College of Medicine, Houston, TX 77030; ^5^Dan L Duncan Comprehensive Cancer Center, Baylor College of Medicine, Houston, TX 77030; ^6^ Institute of Molecular Genetics of the Czech Academy of Sciences, 142 20 Prague 4, Czech Republic; ^7^Institute of Microbiology of the Czech Academy of Sciences, CZ – 14220 Prague 4, Czech Republic; ^8^Center for Cancer Epigenetics, The University of Texas MD Anderson Cancer Center, Houston, TX 77030; ^9^Department of Cell Biology, Faculty of Science, Charles University, 116 36 Prague 1, Czech Republic

**Correspondence:** Frauke Christ

*Retrovirology* 2018, **15(Suppl 1)**:P16

Lens epithelium-derived growth factor/p75 (LEDGF/p75, or PSIP1) is a transcriptional coactivator that tethers other proteins to gene bodies. The chromatin tethering function of LEDGF/p75 is hijacked by HIV integrase to ensure viral integration at sites of active transcription. LEDGF/p75 is also important for the development of mixed-lineage leukemia (MLL), where it tethers the MLL1 fusion complex at aberrant MLL targets, inducing malignant transformation. However, little is known about how the LEDGF/p75 protein Q:11 interaction network is regulated. Here, we obtained solution structures of the complete interfaces between the LEDGF/p75 integrase binding domain (IBD) and its cellular binding partners and validated another binding partner, Mediator subunit 1 (MED1).We reveal that structurally conserved IBD-binding motifs (IBMs) on known LEDGF/p75 binding partners can be regulated by phosphorylation, permitting switching between low- and high-affinity states. Finally, we show that elimination of IBM phosphorylation sites on MLL1 disrupts the oncogenic potential of primary MLL-rearranged leukemic Q:12 cells. Our results demonstrate that kinase-dependent phosphorylation of MLL1 represents an oncogenic dependency which may be harnessed in the treatment of MLL-rearranged leukemia.

**Keywords:** LEDGF/p75; Integrase; IBD; Phosphorylation

### TOPIC 3: Transcription and assembly

#### P17 Exploring the functions of Rev required for viral interference

##### Alexandra Tauzin, Fabrizio Mammano

###### IUH-University Paris-Diderot, Hospital Saint-Louis, Paris, France

**Correspondence:** Alexandra Tauzin

*Retrovirology* 2018, **15(Suppl 1)**:P17

Viral interference is a phenomenon by which infection of a cell by a virus renders it less susceptible to a second infection by the same (or a related) virus [1]. By preventing reinfection, viral interference reduces the extent of cell death by apoptosis and/or by virus-induced toxicity. The best characterized form of interference is due to the occupation of the cellular receptors by the first virus, but receptor-independent viral interference has also been described. A key role of the viral protein Rev in receptor-independent HIV-1 interference was reported by different groups, including our own. In particular, by systematically deleting each viral gene, we could show that in the first 24 h after initial infection, expression of Rev was responsible for the vast majority of interference [2]. Our current aim is to determine the mechanism of Rev-mediated interference.

To this end we have explored the functions of Rev that are required for interference. Rev is a shuttling protein that moves between the nucleus and the cytoplasm. In the nucleus, Rev binds the Rev responsive element (RRE) present in all viral mRNA molecules whose export depends on Rev. Distinct domains in Rev mediate RNA binding, multimerization, nuclear export, and nuclear localization, and mutants were described that knockout these functions. We have thus constructed a series of stably transduced cell lines expressing Rev protein either in its wild-type form, or carrying specific mutations that prevent one or two Rev functions.

The loss of function of Rev mutants, was confirmed by complementation experiments using a Rev-defective virus clone. We next confirmed that expression of wild-type Rev is sufficient to significantly reduce cellular susceptibility to HIV infection. In particular, similar interference levels were observed when we measured the expression of Rev-dependent and -independent reporter genes. Finally, exposure of cells expressing the different Rev mutants to HIV showed that all four main functions of Rev are individually required to establish viral interference. The step of the virus replication cycle affected by Rev is being investigated by dedicated approaches.

In conclusion, our study confirms the significant reduction of susceptibility to infection of cells expressing HIV Rev, and demonstrates that all the main functions of Rev must be preserved for this phenotype.

**Keywords:** Viral interference; Rev; RNA export; Mutants

**References**

1. Nethe M, Berkhout B, van der Kuyl AC. 2005. Retroviral superinfection resistance. Retrovirology 2:52
2. Remion A, Delord M, Hance AJ, Saragosti S, Mammano F. 2016. Kinetics of the establishment of HIV-1 viral interference and comprehensive analysis of the contribution of viral genes. Virology 487: 59–67

#### P18 Independent roles of SL3 stem-loop nucleotides on HIV-1 biological properties

##### Jun-ichi Sakuragi

###### RIMD, Osaka University, Osaka, Japan

**Correspondence:** Jun-ichi Sakuragi

*Retrovirology* 2018, **15(Suppl 1)**:P18

Retroviral genome RNA packaging process is a highly specific and efficient machinery. It is mediated by the interaction between viral gag proteins and cis-acting RNA packaging signals (psi) on viral unspliced RNA. The unspliced RNA species is also utilized as a template for producing the viral Gag and Gag-Pol proteins through translation process. In human immunodeficiency virus type 1 (HIV-1), it is a consensus that about 350 nucleotides from 5’ end of viral unspliced RNA forming complicated conformation operates as a psi. The region contains many essential RNA domains such as TAR, a polyA addition signal, PBS, a major splicing donor, and Gag AUG codon. Some of these domains also play important roles on genome packaging.

SL3, now denoted as “narrowly-defined psi” positions at the lower part in psi and is one of the most important and conserved region in the psi sequence. It contains a GGAG RNA tetraloop that binds nucleocapsid (NC) within viral Gag protein with high affinity [1]. Although structural and biochemical properties of SL3 and NC have been well reported, there are not many consideration about biological properties of SL3 tetraloop nucleotides.

To elucidate the functions of Psi and the mechanism of packaging more precisely, we introduced base-substitution mutation at each base on the tetraloop of proviral clone of HIV-1 (NL4-3). With these mutants, we performed many assays to measure viral activity such as packaging, transcription, virion budding, splicing, genome dimerization, and replication.

In addition, we noticed a unique sequence inserted between SL3 and Gag AUG only conserved among SIV of Apes and several HIV-1 subtypes. We also generated a mutant with this insertion and measured virological properties of it.

So far, the preliminary data shows some of the mutations affect the viral functions other than genome packaging. The studies are now ongoing and the comprehensive results will be presented.

**Keywords:** Viral RNA; Packaging; Genome dimerization; Psi

**Reference**

1. De Guzman RN, Wu ZR, Stalling CC, Pappalardo L, Borer PN, Summers MF: Structure of the HIV-1 nucleocapsid protein bound to the SL3 psi-RNA recognition element. *Science* 1998, 279:384–388.

#### P19 Post-translational modifications of CTIP2 in transcriptional regulation of HTLV-1

##### Sylvain Fauquenoy^1^, Nadège Delacourt^1^, Anna Kula^1^, Anthony Rodari^1^, Caroline Vanhulle^1^, Maxime Bellefroid^1^, Christian Schwartz^2^, Benoit Van Driessche^1^, Virginie Gautier^3^, Olivier Rohr^2^, Carine Van Lint^1^

###### ^1^Service of Molecular Virology, Université Libre de Bruxelles, Gosselies, Belgium; ^2^IUT Louis Pasteur, Université de Strasbourg, Schiltigheim, France; ^3^Center for Research in Infectious Diseases, University College Dublin, Dublin, Ireland

**Correspondence:** Sylvain Fauquenoy

*Retrovirology* 2018, **15(Suppl 1)**:P19

The Human T-Lymphotropic Virus 1 is a complex oncogenic retrovirus infecting around 10 to 20 millions people. HTLV-1 is responsible for two major diseases, an aggressive lymphoproliferative disease (adult T-cell Leukemia/Lymphoma) and a neurological degenerative syndrome (HTLV-1-associated myelopathy). HTLV-1 infection is characterized by viral latency of infected cells and the absence of viremia. These features are thought to be due to transcriptional silencing of proviral expression in vivo, allowing tumor development.

The aim of the present work is to further elucidate the mechanisms involved in HTLV-1 transcriptional silencing and epigenetic control. We focus our work on the contribution of the cellular Sp transcription factors family and CTIP2/Bcl11b transcriptional co-factor. These factors can regulate positively and/or negatively eukaryotic transcription, depending on the promoter and/or the epigenetic environment. Our laboratory has recently demonstrated that CTIP2 is associated to at least two distinct nuclear complexes: the inactive P-TEFb complex, and a chromatin-modifying complex containing histone-deacetylases and -methyltransferase.

We demonstrated that CTIP2 repressed Tax-transactivated HTLV-1 transcription. It has been reported that CTIP2 interacts with the histone acetyltransferase p300 and is involved in transcriptional activation of the IL-2 promoter in T lymphocytes. We postulate that the function of CTIP2 might be modulated by post-translational modifications. We evaluated post-translational modifications of overexpressed CTIP2 protein and identified at least 2 acetylated residues as well as other modified residues. Interestingly, we showed that the substitution of a single particular acetylable residue by an arginine, a non-acetylable residue, impeded global acetylation of CTIP2 and interfered with the ability of CTIP2 to repress Tax-mediated HTLV-1 transactivation. Functional role of the CTIP2 modified residues will be further presented.

A better understanding of the epigenetic and non-epigenetic mechanisms responsible for HTLV-1 transcriptional repression could bring new insights into oncogenic mechanisms associated with CTIP2 transcriptional regulation in humans.

**Keywords:** HTLV-1; Leukemia; Transcriptional regulation; CTIP2/Bcl11b; Latency

#### P20 The Ku-mediated HIV-1 transcription regulation depends on the Ku-RNA binding

##### Olga Shadrina^1^, Irina Garanina^2^, Ekaterina Knyazhanskaya^3^, Marina Gottikh^3,4^

###### ^1^Faculty of Bioengineering and Bioinformatics, Lomonosov Moscow State University, Moscow, Russia; ^2^Federal Research and Clinical Center of Physical-Chemical Medicine of Federal Medical Biological Agency, Moscow, Russia; ^3^Chemical Department, Lomonosov Moscow State University, Moscow, Russia; ^4^Belozersky institute of Physical and Chemical Biology, Lomonosov Moscow State University, Moscow, Russia

**Correspondence:** Olga Shadrina

*Retrovirology* 2018, **15(Suppl 1)**:P20

The Ku protein plays a key role in the DNA double-strand break repair by non-homologous end-joining mechanism. In addition, Ku is considered to participate in HIV-1 replication at the stages of integration and transcription although the exact mechanism of Ku-dependent transcriptional regulation is unclear. We identified RNA structural motifs important for Ku binding. The highest Ku affinity was detected toward a hairpin RNA structure containing a bulk bulge close to the loop. TAR RNA forms a hairpin at the 5’-end of HIV mRNA and has a Ku preferred structure. TAR RNA lacking the bulge had significantly lower affinity towards Ku. We constructed a set of reporter vectors containing firefly luciferase gene under the control of either wild type HIV LTR or its deletion mutants: lacking the region coding for the whole TAR RNA or the region corresponding to the bulge of TAR RNA. CRISPR/Cas9 depletion of Ku in HEK293 led to a significant decrease in luciferase expression from HIV LTR whereas influenced the mutant constructs only slightly. HIV TAR binding protein Tat activates elongation by recruiting P-TEFb complex to HIV promoter for the phosphorylation of RNAP II. Ku overexpression reduced Tat-mediated transcription activation, and Ku depletion increased the effect of Tat. Moreover, the effect of Ku depletion on transcription from LTR promoter was abolished by Tat expression. The catalytic subunit of DNA-dependent protein kinase (DNA-PKcs) is activated upon binding with Ku. *In vitro* binding of Ku with TAR RNA did not lead to DNA-PKcs activation. Depletion of DNA-PKcs had no significant impact on transcription from the reporter vectors. These data give reason to suggest that Ku regulates transcription from LTR promoter due to its interactions with TAR RNA and in a DNA-PKcs-independent manner. We also identified binding of recombinant Ku with hairpins of 7SK RNA that participates in transcription regulation of HIV-1 genes by scavenging P-TEFb in inactive complex. Analysis of data of eCLIP experiments in HepG2 and K562 cells available from the ENCODE confirmed interaction of 7SK RNA and Ku protein. This interaction was also supported by our data on RNA immunoprecipitation assay in HEK293 cells. The Ku-RNA interactions described in our work might be important for understanding the mechanism of Ku-mediated transcription regulation. This work was supported by grants RFBR 18-34-00393 and RSF 14-24-00061 (Ku-TAR binding).

**Keywords:** HIV; Transcription; Ku protein; TAR RNA

#### P21 Factors intrinsic to the virus significantly influence HIV silencing and inducibility

##### Nicholas J Norton^1^, Hoi Ping Mok^1^, Fatima Sharif^1^, Jack C Hirst^1^, Andrew ML Lever^1,2^

###### ^1^Department of Medicine, University of Cambridge, Cambridge, UK; ^2^Yong Loo Lin School of Medicine, Singapore

**Correspondence:** Nicholas J Norton

*Retrovirology* 2018, **15(Suppl 1)**:P21

Transcriptionally silent HIV proviruses form the major obstacle to eradicating HIV. Many studies of HIV latency have focused on the cellular mechanisms that maintain silencing of proviral DNA. We examined viral sequence variations affecting splicing of *tat* mRNA leading to variable rates of silencing and variable responses to reactivation signals.

We studied naturally occurring and engineered polymorphisms in a recently identified exonic splice enhancer (ESE_tat_) that regulates *tat* mRNA splicing and constructed viruses predicted to display increased (M1), reduced (M2) or completely absent (ERK) binding of splicing factors essential for optimal production of *tat* mRNA. The mutations affected viral replication, with M1 having wild type kinetics, M2 exhibiting reduced kinetics and with replication completely abrogated in ERK. Using single round GFP expressing viruses to study proviral gene expression, we observed progressively greater rates of silencing relating to the degree of ESE_tat_ disruption. With WT virus 53% of proviruses were silenced, M2 69% and ERK 94%. By stimulating infected cells with PMA as well as the latency reversing agents panobinostat and JQ1, we observed that the dose required to achieve 50% of the maximum signal was lowest in WT, intermediate in M2 and highest in ERK, indicating a progressively higher threshold for reactivation.

These results suggest that the ability of silent proviruses to reactivate from latency is variable and that minor differences in the viral sequence can alter the proportion of silenced viruses as well as the threshold required to induce silenced viruses to reactivate and express.

**Keywords:** HIV; Latency; Expression; Splicing

#### P22 A closer look at HIV packaging by in-gel RNA-protein SHAPE

##### Ziqi Long^1^, Jingwei Zeng^1^, Andrew Lever^1,2^, Julia Kenyon^1,3,4^

###### ^1^Department of Medicine, University of Cambridge, Cambridge, UK; ^2^Department of Medicine, National University of Singapore, Singapore; ^3^Department of Microbiology and Immunology, National University of Singapore, Singapore; ^4^Homerton College, University of Cambridge, Cambridge

**Correspondence:** Julia Kenyon

*Retrovirology* 2018, **15(Suppl 1)**:P22

HIV packages two copies of its genomic RNA into each virion via interactions with the viral structural protein Gag. For the virion to mature into an infectious particle these genomes must adopt a specific dimeric structure; however whether Gag recognises this dimer for packaging or whether it selects a monomer and aids its structural remodeling remains an area of debate.

We recently published techniques that enable us to probe the structures of different HIV RNA conformers within a mixed structural population (in-gel SHAPE (selective 2’OH acylation analysed by primer extension) [1]) and to pinpoint Gag protein binding sites more accurately, alongside concurrent RNA structural changes (XL-SHAPE [2]). Here, we present a related technique, in-gel RNA–protein SHAPE, which enables us to visualise the RNA structure inside specific nucleoprotein complexes within a mixed population of structures.

To interrogate the structural events surrounding genomic HIV encapsidation the HIV packaging signal RNA was incubated with NC or Gag protein and electrophoresed on native acrylamide gels. Complexes were localised by staining a portion of the gel. The unstained complexes were then probed with SHAPE reagent and electroeluted. Protein was removed with protease treatment and RNA secondary structural changes were modeled by determination of the amount of SHAPE reagent reacting at each nucleotide position. Using this technique, we were able to separately probe monomeric and dimeric RNAs bound to increasing numbers of Gag/NC molecules. The technique is highly reproducible and enables separation and modelling of the RNA structures in complexes of different RNA and protein stoichiometry. Our results support a model where monomeric RNA interacts in the absence of protein to form loose dimers and the structure of these dimers is stabilised and then further altered by Gag binding.

**Keywords:** HIV-1; SHAPE; packaging; RNA structure

**References**

1. Kenyon JC, Prestwood LJ, Le Grice SF, Lever AM. Nucleic Acids Res. 2013 Oct;41(18):e174.
2. Kenyon JC, Prestwood LJ, Lever AM. Sci Rep. 2015 Oct 9;5:14369.

#### P23 DDX17 is an essential factor controlling the HIV-1 A4/5 splice acceptor cluster

##### Nyarie Sithole^1^, Claire Williams^1^, Aisling Vaughan^1^, Julia Kenyon^1,2^, Andrew Lever^1,2^

###### ^1^University of Cambridge, Cambridge, UK; ^2^National University of Singapore, Singapore

**Correspondence:** Andrew Lever

*Retrovirology* 2018, **15(Suppl 1)**:P23

HIV splicing involves 5 splice donor and 8 splice acceptor sequences which, together with cryptic splice sites, generate over 100 mRNA species. 90% of both partially spliced and fully spliced transcripts utilise the intrinsically weak A4/A5 3’ splice site cluster. Using knockdown and rescue we show that DDX17, but not its close paralog DDX5, specifically controls usage of this splice acceptor group. In its absence production of the viral envelope protein and other regulatory and accessory proteins are grossly reduced whilst Vif, which uses the A1 splice acceptor is unaffected. Knockdown of DDX17 is associated with a profound decrease in viral export from the cell. Loss of Vpu expression causing restoration of cellular Tetherin levels compounds the phenotype. The activity of DDX17 is RNA dependent and we identify RNA binding motifs essential for its role whilst the Walker A, Walker B (DEAD), Q motif and the glycine doublet motif are dispensable. DDX17 interacts with cellular splicing factors in a model consistent with it facilitating the interaction between SRSF1/SF2 and the heterodimeric auxiliary factor U2AF65/35.

**Keywords:** HIV; splicing; helicase; DDX17

#### P24 Visualizing the Translation of HIV-1 Full-length RNA

##### Jianbo Chen^1^, Yang Liu^1^, Bin Wu^2^, Vinay K. Pathak^3^, Wei-Shau Hu^1^

###### ^1^Viral Recombination Section, HIV Dynamics and Replication Program, National Cancer Institute at Frederick, Frederick, Maryland, USA; ^2^Department of Biophysics and Biophysical Chemistry, Johns Hopkins University, Baltimore, Maryland, USA; ^3^Viral Mutation Section, HIV Dynamics and Replication Program, National Cancer Institute at Frederick, Frederick, Maryland, USA

**Correspondence:** Jianbo Chen

*Retrovirology* 2018, **15(Suppl 1)**:P24

Full-length HIV-1 RNA plays a central role in viral replication, serving as a template for Gag/Gag-Pol translation and as a genome for the progeny virion. By following individual RNA molecules in living cells, our previous studies have provided important insights into the mechanisms of HIV-1 full-length RNA trafficking and packaging (for simplicity, referred to as HIV-1 RNA hereafter). However, very little is known about HIV-1 RNA translation and how it is regulated. To advance our understanding on these questions, we established a system to visualize protein translation on individual HIV-1 RNA.

Recently, a system has been developed to image protein translation in living cells by detecting fluorescently-labelled RNA and nascent proteins. We have adopted this system to visualize HIV-1 RNA translation. For this purpose, a sequence encoding multiple GCN4 epitopes (Suntag) was inserted into the *gag* gene and Gag-Suntag translation product was detected in living cells using a GCN4-recognizing single chain variable fragment (scFV) fused to super folder GFP (sfGFP). Additionally, stem-loop sequences recognized by bacteriophage MS2 coat protein were inserted into the *pol* gene to enable the detection of HIV-1 RNA. To visualize HIV-1 RNA translation, we transfected the HIV-1 construct into a human cell line expressing scFV-sfGFP and the Halotag-MS2 coat protein and captured images of protein and RNA signals. We observed bright green signals that colocalized and comigrated with RNA signals in the cytoplasm. These bright green spots disappeared rapidly upon treatment of cells of puromycin, which induces premature translation termination and release of nascent polypeptides from the ribosomes, but not cycloheximide, which inhibits translocation of ribosomes on the mRNA and induces translation arrest, indicating that the detected signals represent the scFV-sfGFP bound to the nascent peptides associated with the translating ribosomes. Using this method, we found that ~ 30 to 80% of HIV-1 RNAs in the cytoplasm participated in translation. We are currently studying factors that regulate HIV-1 RNA translation.

**Keywords:** HIV-1; Full-length RNA; Translation; Single molecule imaging

#### P25 Genetic Diversity of CNS-derived LTR modulates HIV-1 Replication in TBM

##### M Fiala^1^, T Ndung’u^1,2,3^, P Madlala^1^

###### ^1^HIV Pathogenesis Programme (HPP), Nelson R Mandela School of Medicine, University of KwaZulu-Natal, Durban, South Africa; ^2^Ragon Institute of Massachusetts General Hospital, Massachusetts Institute of Technology and Harvard University, Boston, Massachusetts, USA; ^3^Africa Health Research Institute (AHRI), University of KwaZulu-Natal, Durban, South Africa

**Correspondence:** M Fiala

*Retrovirology* 2018, **15(Suppl 1)**:P25

The human immunodeficiency virus type 1 (HIV-1) enters the central nervous system (CNS) within two weeks of infection and replicates at lower levels compared to peripheral blood. Interestingly, there are higher RNA levels in cerebrospinal fluid (CSF) than in plasma of tuberculosis meningitis (TBM) co-infected patients. The underlying mechanisms responsible for higher replication in CNS of TBM patients have not been fully characterized. However, genetic polymorphisms within the HIV-1 LTR element that drive virus gene transcription in a cell-specific manner have been described. We hypothesized that TBM patients will display unique LTR genetic mutations in the CSF consistent with higher replication in this compartment.

Viral RNA was extracted from matched CSF and plasma samples obtained from 20 patients (17 TBM and 3 non-TBM) using using QIAmp viral RNA Mini kit.

The extracted viral RNA was reverse transcribed into viral DNA using SuperScript III OneStep kit and nested PCR performed to specifically amplify the U3 region of the LTR using the KAPA HiFi PCR kit. The DNA sequencing was done using BigDye^®^ Sequencing Kit. Generated sequences were manually edited using Sequencher v5.0, and then aligned using ClustlaW default. Phylogenetic analysis to compare intra- and inter-patient diversity was performed by Neighbour-Joining trees with 1000 bootstrap replicates.

The plasma and CSF derived HIV-1 LTR sequences clustered per patient. Notably, 80% of the TBM patients displayed longer branch length for CSF compared to plasma derived LTR. Our data showed that all sequences exhibited 3 NF-κβ binding sites, consistent with features of the HIV-1 subtype C LTR. Although the first and second NF-κβ domains were conserved, the third NF-κβ was variable with 15% of the TBM patients displaying GGGGCGT**G**CC (variants C NF-kB) instead of GGGGC**G**T**T**CC in the CSF. Interestingly patients infected with a mutated C NF-kB virus had significantly lower viral loads compare to those with no mutated C NF-KB (p = 0.03). Approximately 90% of patients with mutations in RBE II binding site also had the most frequent naturally occurring length polymorphism (MFNLP) insertion. The MFNLP is an insertion of approximately 15–30 nucleotides upstream first NF-κβ binding site, which predominantly results in the duplication of RBE III motifs. Functional studies to determine the effect of mutations on the LTR transcription activity are needed.

**Keywords:** HIV-1; LTRs; CNS; TBM

#### P26 Effect of CNS derived Tat diversity on HIV-1 disease in TB Meningitis Patients

##### J. Ramruthan^1*^, T. Ndung’u^1,2,3^, P. Madlala^1^

###### ^1^HIV Pathogenesis Programme, Doris Duke Medical Research Institute, Nelson R. Mandela School of Medicine, University of KwaZulu-Natal (UKZN), Durban, South Africa; ^2^African Health Research Institute, Durban, South Africa; ^3^Ragon Institute of Massachusetts General Hospital, Massachusetts Institute of Technology and Harvard University, Cambridge, USA

**Correspondence:** J Ramruthan

*Retrovirology* 2018, **15(Suppl 1)**:P26

Human immunodeficiency virus type 1 (HIV-1) transactivator of transcription (Tat) is a regulatory protein that activates viral gene transcription. The HIV-1 enters the central nervous system (CNS) and replicates at low levels compared to peripheral blood. However, there is higher HIV-1 RNA levels in the in the cerebrospinal fluid (CSF) compared to plasma of tuberculosis meningitis (TBM) co-infected patients. Consistent with high viral replication in CNS of TBM patients, these patients are shown to have higher CSF *env* diversity compared to plasma. However, the mechanisms that drive higher viral replication in the CNS of TBM patients are not well understood. We hypothesized that TBM patients will display genetically distinct *tat* variants in the CSF as a driver or consequence of higher viral replication in this compartment compared to plasma.

Viral RNA was extracted from matched CSF and plasma samples obtained from 20 patients (17 TBM and 3 non-TBM) using QIAmp viral RNA Mini kit. Extracted viral RNA was reverse transcribed into viral DNA using SuperScript IV and nested PCR was amplified using Platinum Taq High Fidelity PCR kit. DNA sequencing was performed using the BigDye^®^ Sequencing Kit. Sequence editing and analysis was performed using the Sequencher software. Phylogenetic analysis and compartmentalization of *tat* nucleotide sequences from matched CSF and plasma derived viral isolates were aligned using Clustal W and assessed by neighbour-joining phylogenetic analysis using FigTree.

The data from our study show intrasubtype genetic variation which clustered per patient instead of compartment. Our data do not show significant nucleotide differences between the CSF and plasma *tat* sequences. The Tat diversity seen in previously described mutations; E2D, V4I, P21A, K24S, S46Y, P59S and S62G significantly reduce the ability of Tat to transactivate the HIV-1 viral promoter. Conversely, the P21A mutation was associated with an increased viral load in the CSF of TBM patients compared to patients without P21A (p = 0.08). Ongoing experiments include sequencing more samples and a phenotypic assay of different variants.

The P21A mutation in Tat is associated with higher viral replication in the CSF of TBM co-infected individuals. Functional analysis of this mutation in relation to virus replication capacity in cerebrospinal-derived cells is warranted.

**Keywords:** HIV-1; Tat; TB Meningitis

#### P27 A live single molecule localization microscopy study of HIV-1 Gag assembly in host CD4 T cells reveals a spatio-temporal coordination by the viral genome

##### Charlotte Floderer^1^*, Jean-Baptiste Masson^2^*, Elise Boilley^1^, Sonia Georgeault^3^, Peggy Merida^1^, Mohamed El Beheiry^4^, Maxime Dahan^4^, Philippe Roingeard^3^, Jean-Baptiste Sibarita^5^, Cyril Favard^1^ and Delphine Muriaux^1^

###### ^1^Infectious Disease Research Institute of Montpellier (IRIM), UMR9004 CNRS, University of Montpellier, 1919 route de Mende 34293 Montpellier, France; ^2^Decision and Bayesian Computation, UMR 3571 CNRS, Pasteur Institute, Paris, France; ^3^INSERM U966 and IBiSA EM Facility, University of Tours, France; ^4^Light and Optical Control of Cellular Organization, Curie Institute, UMR 168 CNRS, Paris, France; ^5^Interdisciplinary Institute for Neuroscience, UMR 5297 CNRS, University of Bordeaux, Bordeaux, France

*Retrovirology* 2018, **15(Suppl 1)**:P27

The human immunodeficiency virus type 1 (HIV-1) is a retrovirus that infects mainly CD4 T lymphocytes and is still responsible of a pandemia in humans. This virus of approximatively 130 nm in diameter can produce thousands of viruses in one infected T cell. The mechanisms that drive the dynamics of HIV-1 particle assembly in these cells is still not deciphered at the single molecule level. Here, we combine single molecule nanoscopy with cutting-edge quantitative analysis tools to quantify millions of individual viral Gag molecules motions in living CD4 T cells. We observe how single viral Gag proteins are recruited to the budding sites once at the cell membrane and quantify the relative contributions of Gag–Gag protein, Gag-ESCRT or Gag-RNA interaction during this process. Our results show that (1) the average time of HIV assembly in CD4 T cells is 5 min, the particle completion and release needs 15 min, (2) that the Gag-RNA interaction domain is mandatory for the recruitment of Gag at the assembling site, in comparison with capsid–capsid or Gag-ESCRT interactions. Finally, we found that a perfect spatio-temporal coordination of the assembly process only occurs in the presence of the viral genome. Our study provides new insights into the molecular mechanisms of HIV-1 assembly in living host CD4 T cells.

**Keywords:** HIV; CD4 T cells; Gag assembly; Live PALM

### TOPIC 4: Pathogenesis and evolution

#### P28 Characterization of infectious HIV clones from the reservoir of patients

##### Alexandre Nicolas^1,2^, Julie Migraine^1,2^, Jacques Dutrieux^1,2^, Maud Salmona^1,3^, Alexandra Tauzin^1,2^, Jean-Michel Molina^1,2,3^, François Clavel^1,2,3^, Allan J. Hance^1,2^, Fabrizio Mammano^1,2^

###### ^1^INSERM U941, Paris, France; ^2^University Paris-Diderot, Paris, France; ^3^Hospital Saint-Louis, Paris, France

**Correspondence:** Alexandre Nicolas

*Retrovirology* 2018, **15(Suppl 1)**:P28

In the vast majority of HIV-infected patients, combined antiretroviral therapy results in durable control of viremia. A rapid rebound of viremia, however, is almost invariably observed within weeks if treatment is interrupted. Viral rebound is generally considered to originate from viral genomes integrated in resting memory CD4+ T-lymphocytes. While clonal expansion of latently infected cells has been explored, the diversity of replication-competent proviruses present in the reservoir of treated patients has been only partly investigated. We established and validated a workflow to explore the genotype and phenotype of individual clonal viruses from the reservoir of treated-patients. This characterization is relevant for the design of strategies aiming the reduction of the HIV reservoir.

We isolated resting CD4+ T-lymphocytes from 40 ml of peripheral blood. Cells were stimulated by antibodies anti-CD3 and -CD28, and cultured in two different dilution conditions with donor CD4+ T-lymphocytes, to permit clonal virus outgrowth. Viral replication was monitored by p24 quantification, and individual clonal viruses were isolated by short-term culture. We measured the replication kinetics and per-particle infectivities of individual clones from two successfully-treated patients. The near full-length genome of individual clones was sequenced.

Clonal replication-competent viruses from 12 wells from each patient were investigated. Within each patient, individual viral clones exhibited different replication kinetics generating peaks of p24 production between day 4 and 9 (patient 1), or between day 9 and 14 (patient 2). Among viruses that reached peak production on the same day, the amount of virus produced in the supernatant varied extensively. Single-cycle per-particle infectivities differed by 4-fold (patient 1) and 2-fold (patient 2). Taking advantage of the close phylogenetic relatedness of individual clones from each patient, near full-length genome comparison can be used to identify genetic determinants of the observed variances.

This work unveiled differences in the genotypic and phenotypic properties of reservoir viruses from treated patients. The workflow described here can be used to identify treatment conditions (e.g. delay between transmission and treatment initiation, duration of effective treatment, etc.) associated with increased diversity of the viral *quasispecies*.

**Keywords:** Viral reservoirs; Replicative capacity; Genotype; Phenotype

#### P29 Impact of HIV-1 Subtype C Tat genetic variation and evolution on disease outcome

##### Z.Z. Mkhize^1^, T. Ndung’u^1,2,3^, P. Madlala^1^

###### ^1^HIV Pathogenesis Programme, Doris Duke Medical Research Institute, Nelson R. Mandela School of Medicine, University of KwaZulu-Natal, Durban, South Africa; ^2^African Health Research Institute (AHRI), University of KwaZulu-Natal, Durban, South Africa; ^3^Ragon Institute of Massachusetts General Hospital, Massachusetts Institute of Technology, and Harvard University, Cambridge, MA, USA

**Correspondence:** Mkhize ZZ

*Retrovirology* 2018, **15(Suppl 1)**:P29

Although antiretroviral therapy has dramatically reduced human immunodeficiency virus type 1 (HIV-1) associated mortality and morbidity, it is unable to eradicate the virus due to the development of viral reservoir. The HIV-1 transactivator of transcription (Tat) regulates viral gene transcription and is important for pathogenesis [1]. Interestingly, intersubtype and intrasubtype variation in Tat transactivation activity has been reported during chronic infection [2], suggesting that Tat mutations may influence the establishment or reversal of viral latency in vivo. Although it has well been established that HIV-1 latency is established early in infection, there is still paucity of data on the HIV-1 *tat* genetic variation of the transmitter/founder (T/F) virus and diversity during primary infection. The major aim of this study was to characterize the impact HIV-1 *tat* genetic variation during acute infection and evolution by one post infection viral replication. To this effect, viral RNA was extracted from plasma samples obtained during acute and primary infection of 30 acutely infected patients and reverse transcribed using SuperScript IV followed by nested polymerase chain reaction (PCR) amplification using the Platinum Taq DNA polymerase. The PCR products were subsequently sequenced using the BigDye cycle sequencing kit v3.1 and sequences were analyzed using phylogenetic tools. Interestingly, our data demonstrate that T/F virus *that* intrasubtype differences during acute infection and evolved from acute to one year post infection in approximately 37% of the patients. Our data show the at V4I and P21A positions, previously shown to reduce transactivation activity of Tat [3] occurred in approximately 39% of the patients. The core and basic domains of HIV-1 Tat were highly conserved compared to the rest of the protein. Our data demonstrated that the viruses harboring P21A Tat mutation are associated with significantly lower median viral loads compared to patients without this mutation (p = 0.04). Combined effect of previously reported detrimental mutations trended towards lower viral loads compared to enhancing mutations (p = 0.05). Understanding of how HIV-1 gene transcription is regulated, may guide future studies on cure strategies. Ongoing experiments in the laboratory include phenotypic assays of these HIV-1 Tat mutations.

**Keywords:** HIV-1; Tat; Acute; Transactivation

**References**

1. E. Clark, B. Nava, M. Caputi, *Tat is a multifunctional viral protein that modulates cellular gene expression and* functions, volume **8**: 27569–27581
2. U. Neogi, S. Gupta, P.N. Sahoo, A. Shet, S.D. Rao, U, Ranga, V.R. Prasad, *Genetic characterization of HIV type 1 Tat exon 1 from a southern Indian clinical cohort: identification of unique epidemiological signature residues*, **28**, 1152–1156
3. R. Rossenkhan, I. J. Macleod, T. K. Sebunya, E. Castro-nallar, M.F. Mclane, R. Musonda, B. A. Gashe, V. Novitsky, M. Essex, m. 2013. tat Exon 1 exhibits functional diversity during HIV-1 subtype C primary infection. J Virol, 87, 5732–45.

#### P30 Nef-mediated CD3 downmodulation results in decreased lentiviral cell–cell spread

##### Dejan Mesner^1^, Ann-Kathrin Reuschl^1^, Maitreyi Shivkumar^1^, Xenia Snetkov^1^, Tafhima Haider^1^, Dominik Hotter^2^, Frank Kirchhoff^2^, Clare Jolly^1^

###### ^1^Division of Infection and Immunity, University College London, London, UK; ^2^Institute of Molecular Virology, University of Ulm, Ulm, Germany

**Correspondence:** Dejan Mesner

*Retrovirology* 2018, **15(Suppl 1)**:P30

HIV-1 can disseminate by cell-free infection or by direct cell–cell transmission. Cell–cell spread occurs at virological synapses (VS) formed between infected and uninfected CD4 T cells, resulting in polarised viral budding and highly efficient viral spread. Previous work from our lab has shown that VS-formation triggers antigen-independent T cell receptor (TCR) signalling in infected T cells to drive viral spread. Interestingly, the viral accessory protein Nef of most SIV lineages, but not HIV-1, can downmodulate CD3, the signalling component of TCR complex. This impairs signalling at the immunological synapse and is thought to interfere with antiviral responses and prevent aberrant immune activation. Why HIV-1 does not exploit this potential immune evasion strategy has remained enigmatic.

Considering our recent findings, we hypothesised that retaining the TCR-CD3 complex on the surface of infected T cells allows for more efficient cell–cell spread. To assess the role of CD3 downmodulation on cell–cell spread we infected primary CD4 T cells with chimeric HIV-1 NL4.3 viruses expressing different SIVsmm Nef alleles and mutants thereof that differ in their ability to downmodulate CD3.

Our results showed that viral cell–cell spread is diminished upon Nef-mediated CD3 downmodulation. To understand the mechanism of increased cell–cell spread, we used multicolour flow cytometry, phosphoflow and viral assays. We found that retained expression of CD3 was correlated with increased signalling at the VS, increased cell activation and cell death, and an increase in the release of infectious virus from infected T cells.

We conclude that retained expression of CD3 contributes to signalling at the VS and thus enables more efficient cell–cell spread of HIV-1.

**Keywords:** HIV-1; cell–cell spread; TCR signalling; Nef

#### P31 Comparing latency profiles of HIV-1 and HIV-2

##### Anne Bruggemans, Gerlinde Vansant, Paulien Vandevelde, Irena Zurnic, Barbara Van Remoortel, Zeger Debyser

###### Molecular Virology and Gene Therapy, KU Leuven, Leuven, Belgium

**Correspondence:** Anne Bruggemans

*Retrovirology* 2018, **15(Suppl 1)**:P31

During antiretroviral treatment, HIV persists in a reservoir of latently infected cells. This reservoir is now considered the major hurdle for an HIV cure. However, the mechanisms determining latency and reactivation are still not fully understood. HIV-2 is a lentivirus related to HIV-1 that originated from a separate zoonotic transmission. It has remained limited to West-Africa and infection with HIV-2 results in a milder phenotype with slower progression to AIDS and lower viral loads. In contrast, the proviral DNA load appears to be similar, which suggests that HIV-2 may have a more latent phenotype. By comparing these viruses in vitro, we hope to further elucidate the mechanisms governing HIV latency and the effect of latency on the clinical HIV phenotype.

We infected SupT1 cells with single round, HIV-1 and HIV-2 based, VSV-G pseudotyped reporter viruses containing eGFP in the Nef position. Cells were washed on day 3 post-infection and reactivated with tumor necrosis factor alpha (2 ng/ml) or left untreated on day 8. Samples were analyzed after reactivation via flow-cytometry (FC) for eGFP expression and viral p24 protein levels in cell culture supernatant. Additionally, we developed an HIV-2 qPCR to measure copy number. Results show a significantly larger fold reactivation for HIV-1 over HIV-2. When examining the eGFP intensity of eGFP positive cells, HIV-1 has a Gaussian distribution, where HIV-2 seems to create 2 populations, one with low and one with high eGFP intensity. Reactivation is also Gaussian for HIV-1, where for HIV-2 mostly the cells with low eGFP expression seem to reactivate. Different factors may contribute to the observed difference in latency profiles: different levels of viral protein expression, different toxicity of the two viruses and off course different mechanisms of latency establishment and maintenance. Further investigation will be necessary to determine the role of each of these factors moving forward.

**Figure:**
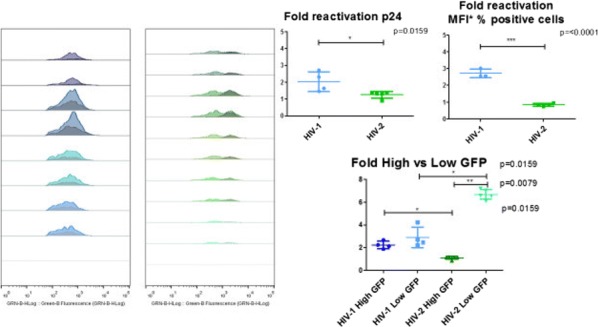
**Figure 1**. Comparison of HIV-1 and HIV-2 reactivation. Results of 1 experiment are shown for different virus dilutions upon infection. **a** Histograms of eGFP intensity for HIV-1 and HIV-2 infected cells before (black) and after (blue or green) reactivation. **b** Fold reactivation (result TNF treated cells/result TNF untreated cells) of HIV-1 and HIV-2 for p24, eGFP expression (as measured by median fluorescence intensity (MFI) × the percentage of positve cells) and percentage of cells with low and high eGFP expression

**Keywords:** HIV-1; HIV-2; Latency; Reactivation

#### P32 Pol-driven replicative capacity impacts disease progression in HIV-1 subtype C

##### Doty B.A. Ojwach^1^, Tarylee Reddy^2^, Vladmir Novitsky^3^, Bruce D. Walker^1,4,5,6^, Daniel MacMillan^7^, Zabrina L. Brumme^7,8^, Mark A. Brockman^7,8^, Thumbi Ndung’u^1,6,9,10^, Jaclyn K. Mann^1^

###### ^1^HIV Pathogenesis Programme, Doris Duke Medical Research Institute, University of KwaZulu-Natal, Durban, South Africa; ^2^ Medical Research Council, Biostatistics Unit, Durban, South Africa; ^3^Harvard T.H. Chan School of Public Health, Boston, MA, USA; ^4^Department of Immunology and Infectious Diseases, Harvard School of Public Health, Boston, MA, USA; ^5^Howard Hughes Medical Institute, Chevy Chase, Maryland, USA; ^6^Ragon Institute of Massachusetts General Hospital, Massachusetts Institute of Technology and Harvard University, Cambridge, MA, USA; ^7^Simon Fraser University, Burnaby, Canada; ^8^British Columbia Centre for Excellence in HIV/AIDS, Vancouver, Canada; ^9^Africa Health Research Institute, Durban, South Africa; ^10^ Max Planck Institute for Infection Biology, Berlin, Germany

**Correspondence:** Jaclyn K. Mann

*Retrovirology* 2018, **15(Suppl 1)**:P32

CD8+ T cell mediated escape mutations in Gag can reduce HIV-1 replication capacity (RC) and alter disease progression, but less is known about immune-mediated attenuation in other HIV-1 proteins. We generated 487 recombinant viruses encoding RT-integrase from individuals with chronic (n = 406) and recent (n = 81) HIV-1 subtype C infection and measured their in vitro RC using a GFP-reporter T-cell assay.

In recently-infected individuals, RT-integrase driven RC correlated significantly with viral load set point (*r *= 0.25; *p *= 0.03) and CD4+ T cell decline (*p *= 0.013). Moreover, significant associations between RT-integrase driven RC and viral load (*r *= 0.28; *p *< 0.0001) and CD4+ T cell count (*r *= − 0.29; *p *< 0.0001) remained in chronic infection. In early HIV infection, host expression of the protective HLA-B*81 allele was associated with lower RC (*p *= 0.05), as was expression of HLA-B*07 (*p *=0.02), suggesting early immune-driven attenuation of RT-integrase by these alleles. In chronic infection, HLA-A*30:09 (in linkage disequilibrium with HLA-B*81) was significantly associated with lower RC (p = 0.05), and all 5 HLA-B alleles with the lowest RC measurements represented protective alleles, consistent with long-term effects of host immune pressures on lowering RT-integrase RC. The polymorphisms V241I, I257V, P272K and E297K in reverse transcriptase and I201V in integrase, all relatively uncommon polymorphisms occurring in or adjacent to optimally-described HLA-restricted CTL epitopes, were associated with reduced RC.

Together, our data suggest that RT-integrase-driven RC is clinically relevant, and provide evidence to support that immune-driven selection of mutations in RT-integrase can compromise RC.

**Keywords**: HIV-1 subtype C; RT-integrase; Replication capacity; HLA-polymorphisms

#### P33 The evolution of ERVs in bats and their potential contribution to the immune response

##### Emilia C Skirmuntt, Aris Katzourakis

###### University of Oxford, UK

**Correspondence:** Emilia C Skirmuntt

*Retrovirology* 2018, **15(Suppl 1)**:P33

Animals host many different types of viruses, allowing them to evolve along with their host. This relationship is typically parasitic in nature, and only benefits the virus. For the animal host, viral infection is a challenge that needs to be overcome. In the host-virus arms race, animals acquire defence mechanisms against pathogens, while pathogens acquire ways to circumvent or subvert those defences. Some viruses achieve this by capturing genes from their host. However, the opposite can also take place: where a host uses viral genes to its own advantage. These viral genes in the host genome can arise when viruses become permanently integrated into a host cell’s genome, either by viral replication (as with retroviruses) or chance (other viruses). If this occurs in germline cells, the integrated virus can be transmitted vertically between generations. Integrations of this kind are called endogenous viral elements (EVEs) or in the case of retroviruses, endogenous retroviruses (ERVs). Occasionally, such sequences can be used by their host, as is the case with EVE-derived immunity (EDI). Thus, while some endogenous sequences are just evolutionary ‘left-overs’, fragmented and mutated remnants of ancient infections affecting our ancestors, some have been altered and used as genes in the viral-host arms-race and act as stand-alone genes in host biological processes. Several examples of such genes have been discovered very recently, one of the most widely studied examples being the retroviral envelope glycoproteins known as syncytins which take part in placental morphogenesis during pregnancy in mammals.

In our study, we screened all publicly available bat genomes from six bat families in which we have identified several envelope sequences of retroviral origin. Retroviral sequences were then analysed with Bayesian methods to generate a phylogenetic tree. Orthology was determined for candidate orthologous sequences by analysis of the genomic neighbourhood, and selection analyses were performed to test for potential function. Our study showed that bat genomes are rich in large and intact potential co-opted envelopes in open reading frames, some of which showed clear evidence of selection. These candidate envelopes allow us to pinpoint ERVs for further laboratory experiments to mark out which of these sequences might have immunosuppressive potential, and if any are substantially expressed in tissues, particularly the placenta.

**Keywords:** Paelovirology; Bats; ERVs; EDIs

### TOPIC 5: Restriction/innate immunity

#### P34 Genetic screens for understanding TRIM5alpha-mediated retroviral restriction

##### Valérie Courgnaud^12^, Lina Castaño^1^, Jean-Luc Battini^1^

###### ^1^Institut de Recherche en Infectiologie de Montpellier, UMR9004 CNRS, Université de Montpellier, Montpellier, France; ^2^Institut de Génétique Moléculaire de Montpellier, UMR5535 CNRS, Université de Montpellier, Montpellier, France

**Correspondence:** Jean-Luc Battini

*Retrovirology* 2018, **15(Suppl 1)**:P34

Many cellular factors that can potentially interfere with the progression of retroviral infections have been identified in mammals. The expression of these restriction factors is often induced by type I interferon and constitute the first line of the host’s innate immune response. Among them, TRIM5alpha, a cellular E3 ubiquitin ligase recognizing the incoming capsid of several retroviruses including HIV, blocks infection before the completion of reverse transcription. The mechanism by which TRIM5alpha prevents viral infection involves a premature uncoating step, but the details of this restriction and the TRIM5alpha-associated cellular proteins remain unknown.

In order to identify new cellular partners involved in TRIM5alpha restriction activity, we have developed a genome-wide loss-of-function screen in human haploid HAP1 cell line by retrovirus-mediated insertional mutagenesis. This cell line contains a fully functional TRIM5alpha gene that allows infection by NB-tropic murine leukemia virus (NB-tropic MLV) but restricts the infection by N-tropic MLV. From a library of 3 millions of independent integration sites, we performed two sequential rounds of infection/selection with N-tropic MLV carrying the *neo* or *blasticidin* resistant genes. After the 2 rounds, we obtained a pool of cells showing a significant increase in N-MLV infection (more than 40%) compared to control HAP1 cells (4%). Three pools of selected cells from 3 independent screens were then subjected to deep sequencing in order to identify gene-trap retrovirus integration sites. In parallel, we isolated single clones and found that they were all fully infectable by N-MLV as efficiently as NB-MLV. The analysis of their integration sites revealed that 3 of them presented independent integrations in the *TRIM5* gene while the other clones had integrations in novel genes never suspected for retroviral infection or restriction. To verify the requirement of these genes in the mechanism of restriction by TRIM5alpha, we are currently testing their capacity to restore the restriction in HAP1 clones stably depleted for the corresponding genes.

Experiments are underway to assess the role of these new genes in TRIM5alpha-mediated HIV restriction.

**Keywords:** TRIM5alpha; Genetic screens; N-tropic MLV; HIV

#### P35 Genome-wide CRISPR-Cas9 screens to identify new interferon-regulated HIV-1 inhibitors

##### Boris Bonaventure, Olivier Moncorgé, Antoine Rebendenne, Caroline Goujon

###### Institut de Recherche en Infectiologie de Montpellier, UMR9004 CNRS, Université de Montpellier, Montpellier, France

**Correspondence:** Boris Bonaventure

*Retrovirology* 2018, **15(Suppl 1)**:P35

Type I interferon (IFN) treatment induces a potent block to HIV-1 infection in primary CD4^+^ T cells, monocyte-derived-macrophages, and in some immortalized cell lines. We, and others, have shown that this block is partially exerted by the interferon-induced dynamin-like MX2 (or MxB) GTPase, which inhibits HIV-1 nuclear import and integration. Importantly, IFN treatment also induces a potent block to HIV-1 DNA accumulation and the cellular factors responsible for this block remain unknown.

In order to identify new cellular effectors of the IFN-induced HIV-1 inhibition, we have developed a whole-genome CRISPR-Cas9 knock-out functional genetic screen. Briefly, the GeCKO library from F. Zhang’s laboratory (MIT, USA) was used to generate cell populations knocked-out for around 19,050 genes. To select mutants unable to inhibit HIV following IFN pre-treatment, the GeCKO cell populations were successively treated with IFN in order to induce interferon-stimulated gene expression, challenged with HIV-1-based lentiviral vectors coding for an antibiotic resistance cassette, selected by drug treatment, and amplified. Several independent screens were performed in 2 model cell lines, with, each time, several rounds of IFN treatment, infection and selection. Next generation sequencing was used to identify the genes that were knocked-out in the final enriched cell populations. Genes belonging to the type 1 IFN response pathway (e.g. IFNAR1, IRF9) represented around 43% of the identified hits, which validated the rationale of the approach. Furthermore, several ISGs were identified as highly enriched, including genes belonging to antiviral effectors families, as well as genes of unknown function. Strikingly, the individual knock-out of some of these genes allowed a partial rescue of HIV-1 infection following IFN treatment, indicating their role in the antiviral state against HIV-1. Moreover, preliminary results show that the overexpression of at least one of these genes is sufficient to partially inhibit HIV-1 infection. The mechanism of action of these new anti-HIV inhibitors is currently being investigated, both in cell lines and primary cells.

This work has been supported by the ATIP-Avenir programme, ANRS, Sidaction, and an ERC Starting Grant

**Keywords:** HIV; interferon; antiviral state; restriction factors; CRISPR-Cas9 whole-genome screen

#### P36 Genome-wide mRNA expression correlates of HIV-1 restriction upon CCL2 blocking

##### Daniela Angela Covino^1^, Jing Lu^2^, Cristina Purificato^1^, Laura Catapano^1^, Mauro Andreotti^1^, Maria Cristina Gauzzi^1^, Matteo Pellegrini^2^, Laura Fantuzzi^1^

###### ^1^National Center for Global Health, Istituto Superiore di Sanità, Rome, Italy; ^2^Department of Molecular, Cell, and Developmental Biology, University of California Los Angeles, Los Angeles, CA, USA

**Correspondence:** Laura Fantuzzi

*Retrovirology* 2018, **15(Suppl 1)**:P36

Residual viremia and low-grade persistent inflammation in cART-treated subjects are nowadays considered the main challenges to achieve a cure. The CCL2/CCR2 axis plays key roles in chronic inflammation in these patients. We found that CCL2 blocking by specific antibodies (Ab) in monocyte-derived macrophages (MDMs) restricts HIV-1 replication by inhibiting viral DNA accumulation independently of SAMHD1. The aim of this study was to identify cellular factors modulated by CCL2 blocking in MDMs and potentially involved in the inhibition of HIV-1 replication.

MDMs were treated with anti-CCL2 or control Ab, and infected with HIV-1BaL. Total RNA was extracted and subjected to poly (A) selection, reverse transcription, generation of cDNA libraries and sequencing on an Illumina Hiseq 2500 platform. Differential expression analysis was done using DESeq2. Genes with logFC ≥ 1 (up-regulated) or ≤ − 1 (down-regulated) and adjusted p value < 0.1 were classified as significantly differentially expressed genes (DEGs). Functional annotation was done using DAVID and STRING. The differential expression profile of some genes was confirmed by qPCR.

In uninfected MDMs, anti-CCL2 Ab treatment for 4 and 20 h resulted in the differential expression of 1558 and 117 genes, respectively. The GO terms enriched in the up-regulated DEGs were associated with immune functions, and those enriched in the down-regulated DEGs were associated with metabolic processes. In HIV-1-infected MDMs, CCL2 neutralization resulted in the differential expression of 79 and 251 genes at 1 and 4 days post-infection. The up-regulated DEGs were associated with immune functions and cytoskeleton organization, whereas down-regulated genes showed no functional enrichment. Among the common up-modulated DEGs annotated in categories related to innate immunity, 16 genes were involved in the defense response to virus. The differential expression profile of some of these genes (APOBEC3A, MX2, and ISG15) was confirmed by qPCR. The alignment of unmapped reads to the HIV-1BaL genome revealed that CCL2 blocking down-modulated viral transcripts.

Overall, these data highlight an association between activation of innate immune pathways and inhibition of HIV-1 gene expression upon CCL2 blocking in macrophages. Thus, targeting the CCL2/CCR2 axis may represent an effective therapeutic strategy to strengthen host innate immunity and control HIV-1 replication.

This work is supported by the Italian Ministry of Health (RF-2011-02347224).

**Keywords:** CCL2; Macrophage; Restriction factor; Transcriptional profiling

#### P37 USP18, a pleiotropic regulator of HIV-1 infection

##### Edmund Osei Kuffour^1^, Ananda Ayyappan Jaguva Vasudevan^1^, Jessica Holler^2^, Baek Kim^2^, Philipp A. Lang^3^, Karl S. Lang^4^, Renate König^5^, Dieter Häussinger^1^, Carsten Münk^1^

###### ^1^Clinic of Gastroenterology, Hepatology and Infectious Diseases, Medical Faculty, University Hospital Düsseldorf, Germany; ^2^Department of Pediatrics, Emory University, Atlanta, Georgia, USA; ^3^Department for Molecular Medicine II, Medical Faculty, University Hospital Düsseldorf, Germany; ^4^Institute of Immunology, University Hospital, University of Duisburg-Essen, Essen, Germany; ^5^Host-Pathogen-Interactions, Paul-Ehrlich-Institut, Langen, Germany

**Correspondence:** Carsten Münk

*Retrovirology* 2018, **15(Suppl 1)**:P37

Innate immune recognition and sensing of HIV-1 is crucial for induction of robust antiviral responses that is characterized by induction of antiviral proteins, such as the type I interferon, which drives a plethora of interferon stimulated genes (ISGs) and activation of antiviral proteins including SAMHD1. How HIV-1 interacts with host cellular factors to escape immune sensing and causes immunopathology is a subject of intense investigation. Here we studied the role of ISG15-specific ubiquitin-like protease 43 (USP18) in HIV-1 innate immune sensing.

HIV-1 infection induces the expression of USP18 in PMA-differentiated THP-1 cells. These cells are resistant to HIV-1 infection due to the expression of unphosphorylated SAMHD1. This restriction is ablated when USP18 is exogenously expressed. We found that USP18 increased HIV-1 infection by more than 40-fold and HIV-2Δvpx by over 7-fold. The increase in HIV-1 infection rose drastically by more than 100-fold in the absence of SAMHD1 in USP18 expressing cells with a dramatic increase in dNTP pool. Knockout of USP18 abrogated significantly the infection of HIV-1 by more than 6-folds, which correlated strongly with reduced phosphorylated SAMHD1.

USP18 blocked the interferon-induced signalling in the THP-1 cells measured by ISG induction by real time PCR. SAMHD1 degradation by VPX protein in wild type THP-1 cells was needed to detect sensing of HIV-1 in differentiated THP-1 cells. However, the expression of USP18 abolished the HIV-sensing as we lose the induction of ISG54 and ISG56.

USP18 bound directly to SAMHD1 in the cell nucleus and complexed with cyclin A, CDK1 and 2. Also USP18 interacted with S-phase kinase associated protein 2 (SKP2), retained cylin A and down regulated p21 in differentiated THP-1.USP18 cells. These activities of USP18 were independent of its ISG15 isopeptidase activity.

This report provides first evidence that USP18 contributes to the block to innate immune sensing of HIV-1.

**Keywords:** HIV; Innate immunity; SAMHD1; Sensing

#### P38 Positive Selection in Viral Restriction Factor SAMHD1 Throughout Mammalian Evolution

##### Christopher Monit^1^, Elizabeth R. Morris^2^, Christopher Ruis^1^, Bartosz Szafran^3^, Grant Thiltgen^1^, N. Avrion Mitchison^1^, Kate N. Bishop^4^, Jonathan P. Stoye^3^, Ian A. Taylor^2^, Richard A. Goldstein^1^, Ariberto Fassati^1^

###### ^1^Division of Infection and Immunity, University College London, London, UK; ^2^Macromolecular Structure Laboratory, The Francis Crick Institute, London, UK; ^3^Retrovirus-Host Interactions Laboratory, The Francis Crick Institute, London, UK; ^4^Infection and Replication of Retroviruses Laboratory, The Francis Crick Institute, London, UK

**Correspondence:** Ariberto Fassati

*Retrovirology* 2018, **15(Suppl 1)**:P38

Viruses and their hosts engage in intricate molecular warfare. A good example is the vertebrate protein SAMHD1, a deoxynucleoside triphosphohydrolase that regulates cellular dNTP concentration, reducing their level below that required by primate lentiviruses, and other DNA synthesizing viruses, to replicate in myeloid cells. To counter this threat, some primate lentiviruses encode accessory proteins that bind to SAMHD1 and target it for degradation. It might be expected that primate SAMHD1 would evolve to evade these virus countermeasures, and indeed evidence of positive diversifying selection has been observed in regions of primate SAMHD1 bound by these lentiviral proteins.

Evolutionary analysis of SAMHD1 has focused on primates, however many mammalian species are infected by retroviruses, including lentiviruses. Furthermore, SAMHD1 reduces the accumulation of endogenous nucleic acids that may trigger innate sensing and inflammation, maintaining at the same time the fine balance of intracellular dNTP levels required for cell survival. It remains unclear If and how these different functions might have shaped SAMHD1 evolution.

We examined the deeper evolutionary history of SAMHD1 by testing whether signatures of positive selection extend to other mammalian groups, and exploring the molecular basis of this co-evolution. Using codon-based likelihood models, we found positive diversifying selection in SAMHD1 across mammals and within each mammal lineage for which sequence data are available. This suggests a molecular ‘arms race’ between SAMHD1 and viruses has been waged throughout mammalian evolution. We observed a significant clustering of sites under positive selection around T592, a residue that is phosphorylated to regulate SAMHD1 by destabilising its tetramer state, reducing catalytic activity. We verified experimentally that mutations within this cluster affect tetramer stability, catalytic rate and HIV-1 restriction, suggesting virus-host co-evolution has required adaptation both at the interaction interfaces with viral proteins and at the level of enzymatic function. Thus persistent positive selection may have involved the adaptation of SAMHD1 function and regulation.

**Keywords:** SAMHD1; Mammals; dNTPs; Evolution

#### P39 Efficient SERINC5 antagonism does not enhance HIV-1 replication and cytopathicity

##### Dorota Kmiec^1^, Bengisu Akbil^1^, Swetha Ananth^2^, Dominik Hotter^1^, Konstantin Sparrer^1^, Birthe Trautz^2^, Ahidjo Ajouba^3^, Martine Peeters^3^, Oliver T Fackler^2^, Frank Kirchhoff^1^

###### ^1^Institute of Molecular Virology, Ulm University Medical Center, Ulm, Germany; ^2^Department of Infectious Diseases, University Hospital Heidelberg, Heidelberg, Germany; ^3^Laboratoire Retrovirus, IRD and Université Montpellier, Marseille/Montpellier, France

**Correspondence:** Dorota Kmiec

*Retrovirology* 2018, **15(Suppl 1)**:P39

SERINC5 is a restriction factor that impairs infectivity of HIV-1 and other primate lentiviruses and is counteracted by the viral accessory protein Nef [1, 2]. SERINC5 antagonism is highly conserved among primate lentiviral Nefs [3] but its relevance for viral replication and cytopathicity remained to be determined. Here, we show that Nef proteins of the highly divergent SIVcol lineage infecting mantled guerezas (Colobus guereza) are potent antagonists of SERINC5 [3], although they lack the CD4, CD3 and CD28 downmodulation activities exerted by other primate lentiviral Nefs. In addition, SIVcol Nef decreases CXCR4 cell surface expression, suppresses TCR-induced actin remodelling, and counteracts colobus but not human tetherin to promote virus release. Unlike HIV-1 Nef, SIVcol Nef induces efficient proteasomal degradation of SERINC5 and counteracts orthologues from frogs and fish. We identified a single tyrosine residue (Y86) in SIVcol Nef that is critical for SERINC5 antagonism but not for CXCR4 downmodulation. This allowed us to engineer HIV-1 proviral constructs differing in their ability to counteract SERINC5 to analyse the relevance of this function for HIV-1 replication. We found that Nef-mediated SERINC5 counteraction modestly enhanced viral infectivity but did not significantly affect viral replication and CD4+ T cell depletion in ex vivo infected human CD4+ T cells and lymphoid tissues. In conclusion, SIVcol Nef lacks several activities that are conserved in other primate lentiviruses and uses a distinct mechanism to counteract SERINC5. Our finding that evolutionarily distinct SIVcol Nefs show potent anti-SERINC5 activity supports a relevant role of SERINC5 antagonism for viral fitness in vivo. However, the reasons for this need further study since SERINC5 counteraction did not enhance viral replication in primary human cells and ex vivo infected lymphoid tissue. Viral constructs expressing SIVcol Nefs differing specifically in anti-SERINC5 activity will be useful tools to examine the relevance of this restriction factor in vivo.

**Keywords:** SERINC5; Nef; HIV-1; SIVcol

**References**

1. Rosa A. et al. HIV-1 Nef promotes infection by excluding SERINC5 from virion incorporation. Nature. 2015; 526:212–217.
2. Usami Y. et al. SERINC3 and SERINC5 restrict HIV-1 infectivity and are counteracted by Nef. Nature. 2015; 526:218–223.
3. Heigele A. et al. The Potency of Nef-Mediated SERINC5 Antagonism Correlates with the Prevalence of Primate Lentiviruses in the Wild. Cell Host Microbe. 2016; 20:381–391.

#### P40 Regulation of SAMHD1 restriction activity by SUMOylation

##### Charlotte Martinat^1^, Arthur Cormier^1^, Noé Palmic ^1^, Joëlle Tobaly-Tapiero^1^, Si Ana Coggins ^2^, Kim Baek^2^, Ali Saïb^1,3^, Alessia Zamborlini^1,3^

###### ^1^Inserm U944 – CNRS/P7 UMR 7212, IUH, Paris, France; ^2^Emory School of Medicine, Atlanta, USA; ^3^Laboratoire PVM Cnam, Paris, France

**Correspondence:** Alessia Zamborlini

*Retrovirology* 2018, **15(Suppl 1)**:P40

SAMHD1 is a cellular triphosphohydrolase (dNTPase) that degrades dNTPs and inhibits HIV-1 replication at the reverse transcription step in non-cycling immune cells. Viruses of the HIV-2/SIVsmm lineage overcome this restriction by encoding the Vpx protein which inactivates SAMHD1 by inducing its proteasomal degradation.

How SAMHD1-dependent restriction is regulated has been subjected to intense investigation in recent years. Phosphorylation of residue T592 is proposed to downregulate the anti-HIV-1 function of SAMHD1 in cycling cells. Accordingly, non-cycling cells expressing of a phosphomimetic mutant (T592D/E) support HIV-1 infection. However the SAMHD1 T592D/E mutant (unable to block HIV-1 infection) can still hydrolyze dNTPs. On the other hand, expression of an unphosphorytable mutant (T592A) is not sufficient to render cycling cells resistant. These data raise the possibility that SAMHD1-mediated restriction may not exclusively rely on its dNTPase activity, and/or its regulation may not depend only phosphorylation of T592.

We found that SAMHD1 modified by Small Ubiquitin-like modifier (SUMO) proteins and identified three conjugation sites (CS-1 to -3), one of which overlaps with the Vpx-interaction interface. Next, we expressed SUMOylation-defective SAMHD1 variants in U937 cells which were differentiated by PMA treatment and infected with HIV-1, HIV-2 or HIV-2ΔVpx to test restriction. Mutation of CS-1 and -3 either alone or in combination do not alter the antiviral activity of SAMHD1. In contrast, inactivation of CS-2 renders SAMHD1 unable to restrict HIV-1 and HIV-2ΔVpx. notably, all variants lower the dNTP pool to the same extent as WT SAMHD1 indicating that their dNTPase activity is intact.

Our data showing that SUMO-conjugation at CS-2 is required for antiviral activity but not dNTP hydrolysis further support the notion that the two functions of SAMHD1 can be dissociated.

**Keywords:** SAMHD1; HIV; Restriction; SUMOylation

#### P41 HIV-2 and HIV-1 Vifs Target APOBEC3G to Different Protein Degradation Pathways

##### Dongfei Qi^1^, Belete A. Desimmie^1^, Ryan C. Burdick^1^, Michael Nekorchuk^1^, Narasimhan J. Venkatachari^1^, Scott Martin^2^, Eugen Buehler^2^, Wei-Shau Hu^3^, and Vinay K. Pathak^1^

###### ^1^Viral Mutation Section, HIV Dynamics and Replication Program, National Cancer Institute at Frederick, Frederick, Maryland, USA; ^2^National Center for Advancing Translational Sciences, National Institutes of Health, Rockville, Maryland, USA; ^3^Viral Recombination Section, HIV Dynamics and Replication Program, National Cancer Institute at Frederick, Frederick, Maryland, USA

*Retrovirology* 2018, **15(Suppl 1)**:P41

Lentiviral Vif proteins overcome inhibition by host APOBEC3 proteins by inducing their degradation. HIV-1 Vif (Vif1) induces MG132-sensitive proteasomal degradation of APOBEC3 proteins; consequently, all lentiviral Vifs are thought to induce APOBEC3 proteasomal degradation, but few studies have examined the mechanism by which other lentiviral Vifs block APOBEC3 restriction. Vif1 and HIV-2 Vif (Vif2) exhibit only ~ 30% sequence identity, and we recently showed that completely different structural determinants of Vif2 and APOBEC3G (A3G) are essential for their interaction and A3G degradation. Here, we examined the mechanism by which Vif2 induces degradation of A3G, and surprisingly found that Vif2 induces A3G degradation by utilizing a bafilomycin A1-sensitive lysosomal degradation pathway. Vif1 and Vif2 both interact with elongin b/c, but their interactions with cullin 5 and cullin 2, respectively, are essential for A3G degradation. While poly-ubiquitination involving ubiquitin K48 linkages is necessary for Vif1-mediated A3G degradation, none of the ubiquitin lysines are essential for A3G degradation by Vif2, indicating that multi-ubiquitination or linear ubiquitination is sufficient for Vif2-mediated A3G degradation. HIV-2 and SIV of rhesus macaques (SIVmac) arose from transmission of SIV sooty mangabey (SIVsm) into humans and rhesus macaques, respectively. Interestingly, SIVsm and SIVmac Vifs utilized both the proteasomal and the lysosomal degradation pathways. Taken together, these results show that Vif1 and Vif2, which evolved in different non-human primate hosts, adapted to overcome the APOBEC3 restriction factors by exploiting distinct cellular protein degradation pathways.

**Keywords:** HIV-2; Vif; APOBEC3G; Lysosomal degradation

#### P42 Insights into the role of Proteasome Inhibitors as HIV-1 latency reversal agents

##### Uddhav Timilsina, Dibya Ghimire, Shilpa Sharma, Ritu Gaur

###### Faculty of Life Sciences and Biotechnology, South Asian University, New Delhi, India

**Correspondence:** Ritu Gaur

*Retrovirology* 2018, **15(Suppl 1)**:P42

Patients suffering from HIV/AIDS are treated using Antiretroviral therapy (ART) which involves a combination of at least 3 drugs targeting different steps in the viral life cycle. Despite more than 25 FDA approved antiviral drugs currently available, complete cure for HIV/AIDS is not acheivable. This is due to presence of persistent residual virus in ART-treated patients arising from latent viral reservoirs and/or residual viral replication. The mechanism of HIV latency and the size of the viral reservoir is poorly understood. Lack of availability of potent latency-reversing agents (LRAs) and resistance to immunological clearance of reactivated viruses further complicates the HIV-1 eradication strategy. Though several classes of molecules can reactivate latent HIV-1, very few of them have entered human clinical trials. Recently, proteasome inhibitors (PIs) were reported to act as HIV-1 LRAs. We systematically analysed the effect of several PIs to reactivate latent HIV-1. We observed that the PIs could not only reactivate latent HIV-1, but they also led to production of non-infectious viruses. We explored the mechanism for the loss of infectivity of PI reactivated virus using several virological and biochemical assays. We observed that a family of HIV restriction factors, APOBEC3 plays an important role in reducing viral infectivity. These proteins are packaged in the reactivated viruses in Vif independent manner resulting in hypermutation of the viral genome. Our study highlights the potential of Bortezomib as a bifunctional HIV-1 antagonist and suggests that it can inhibit the Vif-APOBEC3 interaction. Further ongoing work on unravelling the detailed mechanism of action of Bortezomib as LRAs will be discussed.

**Keywords:** HIV-1 latency; Viral reservoir; Proteasome inhibitors; APOBEC3

#### P43 The anti-retroviral activity of human SERINC genes

##### Cinzia Bertelli^1^, Claudia Firrito^1^, Teresa Vanzo^1^, Ajit Chande^2^, Massimo Pizzato^1^

###### ^1^Centre for Integrative Biology, University of Trento, 38123 Trento, Italy; ^2^Department of Biological Sciences, Indian Institute of Science Education and Research Bhopal, Bhopal 462 066, Madhya Pradesh, India

**Correspondence:** Cinzia Bertelli

*Retrovirology* 2018, **15(Suppl 1)**:P43

The multipass transmembrane proteins SERINC5 and SERINC3 inhibit retrovirus infectivity and are counteracted by Nef of primate lentiviruses, by glycoGag of gammaretroviruses and by S2 of equine infectious anemia virus. SERINC5 was also found to alter the sensitivity of HIV-1 to neutralizing antibodies (nAbs) targeting the MPER in gp41. Among the five human SERINC paralogs, it has been established that SERINC2 does not affect infectivity nor susceptibility to nAbs. However, the activities of SERINC1 and SERINC4 remain to be investigated.

We observed that, similarly to SERINC3, SERINC1 carries a modest anti-retroviral activity. In contrast, SERINC4 exerts a potent activity against HIV-1 even at low expression levels. All SERINC paralogs are equally effective at inhibiting HIV-1 and MLV, indicating a similarly broad anti-retroviral activity. The ability of inhibiting HIV-1 infectivity mirrors also the effect of the SERINC paralogs on HIV-1 susceptibility to neutralization by antibodies targeting MPER, suggesting a functional link between the effects on infectivity and neutralization.

Irrespectively of their potency, all SERINC proteins are incorporated into virus particles. However, we observed that virion-associated SERINC2 and SERINC1 are specifically and efficiently cleaved within the fourth intracellular loop, indicating that once incorporated, these proteins are accessible to the HIV-1 protease. Given that SERINC1 and SERINC2 display poor antiviral activity, we are currently investigating whether proteolysis affects the ability of both proteins to inhibit infectivity.

Having observed the strong activity of SERINC4 against HIV-1, we studied its expression levels in blood cells. Our data indicate that SERINC4 is not expressed in cells that are the natural target of HIV-1, such as CD4+ T-lymphocytes. Furthermore, according to public RNAseq databases, the SERINC4 transcript is poorly detected or absent in any human tissues, raising questions about the regulatory mechanisms governing its expression level. Interestingly, transfection of cells with the native human SERINC4 cDNA does not result is any detectable protein expression and inhibits production of a green fluorescence protein from the same cistron. We therefore hypothesize that a yet unknown post-transcriptional mechanism could regulate SERINC4 expression.

Altogether, the role of the different SERINC paralogs in retrovirus infection, as well as their cellular core function, remain elusive.

**Keywords:** Restriction factor; SERINC5; Infectivity; HIV-1

#### P44 Modulation of HIV infection and nuclear morphology by SUN1 and SUN2

##### Anvita Bhargava^1^, Xavier Lahaye^1^, Mathieu Maurin^1^, Mabel Jouve^1^, Nicolas Manel^1^

###### ^1^Institut Curie U932, 12 Rue Lhomond, 75005 Paris, France

**Correspondence:** Anvita Bhargava

*Retrovirology* 2018, **15(Suppl 1)**:P44

The nuclear envelope (NE) is a critical barrier that HIV must cross to establish infection. The viral capsid plays a determining role in nuclear entry and genome invasion. These processes implicate interactions of the capsid with host factors. We have previously shown that the NE protein SUN2 promotes cyclophilin A (CypA)-dependent steps of HIV infection in primary human CD4+ T cells, mouse dendritic cells and HeLa cells. However, how SUN2 modules viral infection is not understood. SUN2 and its paralog, SUN1, are localized at the inner nuclear membrane and are constituents of the LINC complex that anchors the nucleus to the cytoskeleton. Here, we have studied the impact of modulating levels of SUN1 and SUN2 expression on infection and NE architecture. We show that overexpression of both SUN1 and SUN2 leads to reduced infection by HIV-1 and HIV-2 in HeLa cells and primary monocyte-derived macrophages (MDMs). We find that the anti-viral activity of SUN1 and SUN2 over-expression is cell-intrinsic. Disruption of SUN-Nesprin interaction through expression of a dominant-negative KASH domain shows that antiviral activity is independent of LINC complex formation. Interestingly, SUN1 and SUN2 show strain specificity by restricting more potently HIV-1 and HIV-2, respectively. By generating chimaeric proteins between SUN1 and SUN2, we show that this selectivity is driven by their nucleoplasmic, N-terminal domains that are known to interact with nuclear lamins. SUN1 and SUN2 overexpression, though not impacting viability, also leads to changes in NE morphology resulting in deformed, lobulated nucleus. Interestingly, Lamin A/C depletion induces similar reduction of nuclear circularity as SUN1 or SUN2 overexpression but does not inhibit HIV infection, indicating that nuclear deformation is not sufficient to induce an antiviral effect. We also find that endogenous Lamin expression is not required for the antiviral activity of SUN overexpression. Finally, we show that the SUN1 and SUN2 overexpression abolishes the effects of CypA on HIV-1 and HIV-2 in monocyte-derived macrophages. We are currently exploring further the relationship between nuclear deformations induced by SUN proteins, HIV infection and CypA. Overall, our results highlight the dynamic role of NE and its components in HIV infection.

**Keywords:** HIV; Nuclear Envelope; SUN; Cyclophilin A

#### P45 Peptides derived from Beclin1 modulate antiviral restriction during virus entry

##### Saliha Majdoul and Alex A. Compton

###### HIV Dynamics and Replication Program, National Cancer Institute, Frederick, MD USA

**Correspondence:** Saliha Majdoul

*Retrovirology* 2018, **15(Suppl 1)**:P45

Human cells have evolved multiple strategies to protect against viral infections, but a complete understanding of the antiviral pathways that restrict infection is lacking. Recently, a synthetic fusion peptide between cellular protein Beclin1 and lentiviral protein Tat (Tat-Beclin1 or TB1) was shown to inhibit the replication of several viruses, including human immunodeficiency virus type 1 (HIV-1) in a process purported to involve autophagy. We subsequently studied the action of TB1 on infection mediated by lentiviral vectors and found, unexpectedly, that it increased infection by promoting the virus-cell adhesion and fusion. Our findings highlighting an enhancement effect of TB1 contrasts with its previously reported inhibitory activity yet was apparent for vectors derived from both HIV-1 and AAV8 (Adeno-associated virus 8). Therefore, the actions of the peptide can either inhibit or promote infection, warranting a closer examination of its precise mode of action. To this end, we have explored the roles played by TB1 during early and late stages of infection by using replication-incompetent pseudoviruses as well as infectious HIV-1. Here we report that TB1 is taken up into the cell interior and facilitates lentiviral infection in a cell type- and viral envelope glycoprotein-dependent manner, indicating the involvement of a cellular factor regulating virus entry. Using RNA interference and CRISPR/Cas9, we tested the hypothesis that TB1 interferes with the interaction between Beclin1 and one or more of its interaction partners (UVRAG, Atg14L, and GAPR-1). Furthermore, we employed mass spectrometry to reveal systems-wide effects on the cellular proteome. Our collective efforts towards understanding the molecular footprint of TB1 suggest the existence of a novel restriction pathway impacting HIV-1 and other viruses.

**Keywords:** HIV; Autophagy; Endocytosis; Gene therapy

### TOPIC 6: Other retroviruses

#### P46 Characterization of HERV9 Elements within the Human Genome

##### Keylie M Gibson^1^, Gary A Hovespian^2^, Miguel de Mulder^2^, R Brad Jones^2^, Keith A Crandall^1^, Douglas F Nixon^2^, Matthew L Bendall^1,2^

###### ^1^Computational Biology Institute, Milken Institute of Public Health, The George Washington University, Ashburn, VA, USA; ^2^Department of Microbiology, Immunology, & Tropical Medicine, School of Medicine and Health Sciences, The George Washington University, Washington, DC, USA

**Correspondence:** Keylie M Gibson

*Retrovirology* 2018, **15(Suppl 1)**:P46

Human Endogenous Retroviruses (HERVs) are the genomic remains of ancient retroviruses that infected vertebrate genomes millions of years ago. Over evolutionary time, these proviruses have lost their infectious capacity due to an accumulation of mutations in the coding regions and long terminal repeats (LTRs), and most are believed to be transcriptionally silent in normal human tissue. However, recent evidence has shown several mechanisms by which HERV expression can influence homeostatic processes, including alternative enhancers for protein coding genes, activation of non-coding genomic regions, and expression of retroviral transcripts or proteins. The HERV9 family of endogenous retroviruses is of particular interest because it represents one of the more recent endogenization events, and is thus expected to retain more of its functional capacity than older HERV families. Despite this relatively young evolutionary age, HERV9 has been given relatively little attention compared to other HERV families such as HERV-W and HERV-K (HML-2). Finally, HERV9 and its long terminal repeat, LTR12, has been shown to regulate the activity of certain proapoptotic genes involved in prevention of cancer, specifically TP63 and TNFRSF10B in testicular cancer. In the present work, we identified and detailed the location and genomic context of 190 HERV9 elements in humans. Using Telescope, this bioinformatic analysis has led to a characterization of all near-complete HERV9 elements in the human reference genome (hg38), with a report on the genomic and epigenomic context of their insertions throughout the genome and a phylogenetic classification of HERV9 subfamilies. Our exploratory analyses show dynamic connectivities within the HERV9 families. This body of work illustrates the importance of HERV9 elements and possible contributions to human homeostasis and pathogenesis. The goal of our study is to provide an exhaustive reference library for HERV9 to be used in understanding its role in both pathology and cooption throughout human evolution.

**Figure:**
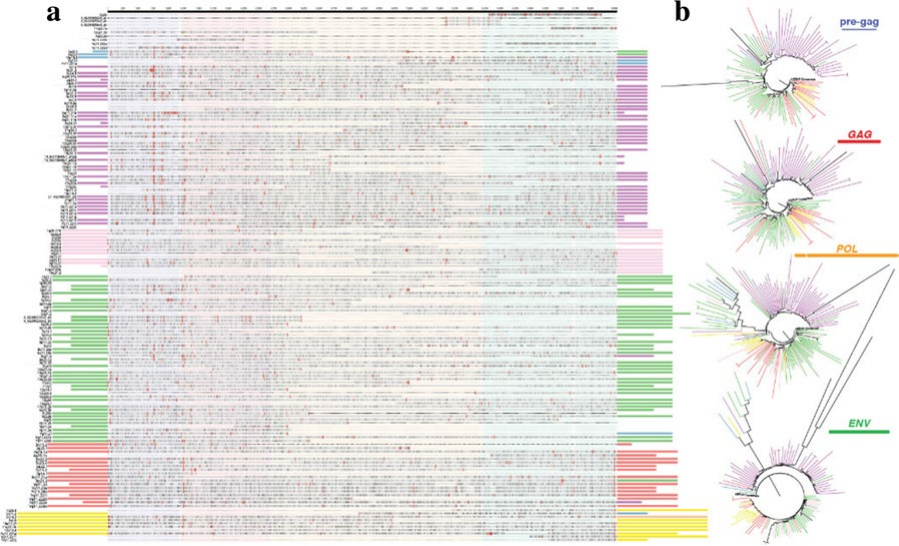
**Figure 1**. HERV9 elements in the human genome (hg38) and their phylogenetic relationships

**Keywords:** HERV9; retrovirus; Telescope; LTRs

#### P47 Expansion microscopy (ExM) as a novel tool to follow retroviral replication

##### Aline Acke^1^, Flore De Wit^2^, Doortje Borrenberghs^1^, Susana Rocha^1^, Volker Leen^1^, Zeger Debyser^2^, Johan Hofkens^1^, Kris P.F. Janssen^1^

###### ^1^Molecular Imaging and Photonics, KU Leuven, Leuven, Belgium; ^2^Molecular Virology and Gene Therapy, KU Leuven, Leuven, Belgium

**Correspondence:** Aline Acke

*Retrovirology* 2018, **15(Suppl 1)**:P47

Discovery of effective anti-viral agents is an arduous process. While potential therapeutic agents targeting host-factors have on occasion been shown to successfully interfere with the replication of viruses, these efforts are often curtailed due to e.g. toxicity effect or poor efficacy in vivo. To increase success in discovering new therapeutics a better understanding of the infection process is needed. Fluorescence microscopy is a powerful tool to shed further light on the interaction between viral pathogens and host cellular pathways. Efforts to access this world experimentally has triggered the development of so called super resolution fluorescence microscopy (SRFM). Here, spatial and or temporal modulation of illumination light allowed scientists to visualize structures with resolutions that exceed the diffraction limit (~ 200 nm). Although effective, these approaches still impose a great number of experimental preconditions, which might impede wide-spread use of SRFM modalities in drug discovery. More recently, an alternative to SRFM was proposed where, instead of engineering the microscope, the sample was modified in order to obtain higher resolutions by physically expanding it, referred to as **expansion microscopy** (ExM). By infusing biological samples with suitable monomers, a super-absorbent polymer can be formed throughout the sample, which can subsequently be expanded and produce a perfectly transparent matrix, to which the biomolecules of interest are cross-linked such that their original geometry is preserved (Fig. 1). The versatility of ExM allows for imaging fundamental biomolecules at resolutions exceeding the diffraction limit on simple, diffraction limited microscopes. ExM is promising in the field of virology, where besides the diffraction limit, the number of dyes is a limiting factor for the use of other existing SRFM methods. For the first time, an accurate study of viruses via conventional fluorescence microscopy seems within reach, providing new insights into the nuclear entry of retroviruses such as human immunodeficiency virus 1 and murine leukemia virus (MLV). More specifically, ExM can be used in an accurate and visual comparison between localization of integration sites among a MLV wild type and MLV mutant.

**Fig. 1:**
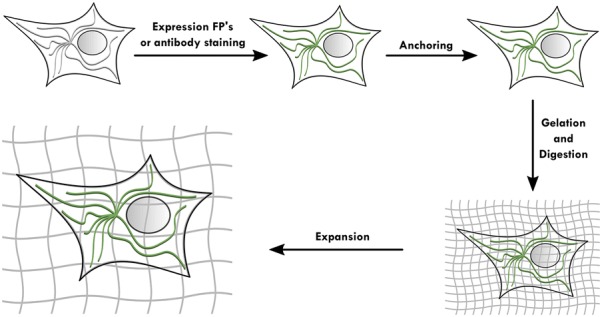
Principle ExM: structures of interest are labelled and functionalized with a chemical compound (anchoring) that crosslinks with the gel. After gelation and digestion, expansion is performed (Tillberg et al., 2016)

**Keywords:** Fluorescence microscopy; Expansion microscopy; Murine leukemia virus MLV; Pre-integration complex

#### P48 Genetic variability of small ruminant lentiviruses between blood and colostrum

##### Monika Olech, Jacek Kuźmak

###### Department of Biochemistry, National Veterinary Research Institute, Pulawy, Poland

**Correspondence:** Monika Olech

*Retrovirology* 2018, **15(Suppl 1)**:P48

Small ruminant lentiviruses (SRLV), comprising caprine arthritis encephalitis virus (CAEV) and maedi visna virus (MVV), are responsible for a persistent infection which induces chronic inflammatory lessons in joints and central nervous system in goats and inflammation of lung and mammary gland in infected sheep. In goats, ingestion of colostrum and milk from infected mothers has a significant importance in virus transmission to their offspring. High genetic diversity of SRLV can generate the population of variants which can be compartmentalized after virus adaptation to certain cells or tissues. This study aimed to analyze heterogeneity of *env (V4V5)*, *gag (CA)* and LTR sequences of proviral DNA from 3 goats naturally infected with a newly identified subtype A17 SRLV, in order to assess virus compartmentalization in colostrum. The results showed that different genes evolved differently. Intra-host genetic diversity was lower for *gag* and LTR fragments compared to *env* gene. Compartmentalization was assessed using six assays, based on topology of phylogenetic trees or calculation genetic distances between sequences. Statistically significant evidence of compartmentalization between sequences in blood and colostrum was found in all goats on the basis of all analyzed fragments, however, blood and colostrum derived sequences were intermingled on phylogenetic trees. The codon selection and signature sequence analysis failed to identify convincing patterns in all examined samples. Nevertheless, dN/dS values of some sequences, especially *env* fragment, were substantially higher, suggesting that some residues may be subjected to positive selection. Statistically significant differences in the numbers of potentially N-glycosylation sites were noted between blood and colostrum *env* sequences. Altogether, this study demonstrated that compartmentalization between blood and colostrum concerns not only *env* sequences but also *gag* and LTR of subtype A17 SRLV. The characterization of distinct virus subpopulationsin colostrum will be useful for better knowledge of biological proprieties of the virus and its transmission.

**Keywords:** Compartmentalization; Small ruminant lentiviruses; Colostrum, Goat

#### P49 New insights into Bovine Leukemia Virus (BLV) transcriptional regulation

##### Maxime Bellefroid^1,*^, Estelle Plant^1,*^, Anthony Rodari^1^, Benoit Van Driessche^1^, Sylvain Fauquenoy^1^, Caroline Vanhulle^1^, Lorena Nestola^1^, Arsène Burny^1^, Carine Van Lint^1^

###### ^1^Service of Molecular Virology, Department of Molecular Biology (DBM), Université Libre de Bruxelles (ULB), Gosselies, Belgium

**Correspondence:** Maxime Bellefroid

*Retrovirology* 2018, **15(Suppl 1)**:P49

*These authors contributed equally to this work

Bovine leukemia virus (BLV) is a B-lymphotropic oncogenic deltaretrovirus infecting cattle and closely related to human T-cell leukemia viruses I and II (HTLV-I and II). Despite the well-established repression of the 5’LTR-driven viral gene expression, we and others have discovered and characterized two alternative viral promoters [1, 2, 3, 4], allowing a high expression of viral miRNAs [2, 3] and antisense viral transcripts [4], potentially contributing to tumor development. In addition, our data have suggested a collision phenomenon between the RNAPIII transcribing the miRNA cluster and the RNAPII coming in an antisense orientation from the 3’LTR [1]. These latter results have indicated that transcriptional interference could be seen as a new mechanism used by BLV to regulate its three transcriptional activities.

In this work, we investigated the interplay between the three BLV promoter activities and showed putative critical functions of the transcriptional interference to drive or repress BLV transcriptional activities. In addition, we highlighted the implication of new transcription factors in BLV transcriptional and epigenetic regulations but also in BLV-mediated pathogenesis.

Overall in this study, we further investigated new alternative ways used by BLV to regulate its transcriptional and epigenetic status and provided new fundamental insights into BLV transcriptional and epigenetic regulations which could explain the escape from the host immune system and/or the BLV-induced pathogenesis.

**Keywords:** Bovine Leukemia Virus; Transcription; Epigenetic; Regulation

**References**

1. Van Driessche B., Rodari A., Delacourt N., Fauquenoy S., Vanhulle C., Burny A., Rohr O., Van Lint C., *Characterization of new RNA polymerase III and RNA polymerase II transcriptional promoters in the Bovine Leukemia Virus genome*, Sci Rep 6, **2016**.
2. Rosewick N., Momont M., Durkin K., Takeda H., Caiment F., Cleuter Y., Vernin C., Mortreux F., Burny A., Georges M., and Van den Broeke A., *Deep sequencing reveals abundant noncanonical retroviral microRNAs in B*-*cell leukemia/lymphoma,* PNAS, 110, **2013**.
3. Kincaid R. P., Burke J. M., Sullivan C. S., *RNA virus microRNA that mimics a B*-*cell oncomiR*, PNAS, 109, **2012**.
4. Durkin K., Rosewick N., Artesi M., Hahaut V., Griebel P., Burny A., Georges M., Van den Broeke A., *Characterization of novel Bovine leukemia Virus (BLV) antisense transcripts by deep sequencing reveals constitutive expression in tumors and transcriptional interaction with viral a microRNAs,* Retrovirology 13:33, **2016.**

### TOPIC 7: Novel antiviral strategies

#### P50 Avian Leukosis Virus Receptors and Biotechnological Approach to Host Resistance

##### Jiří Hejnar^1^, Jiří Plachý^1^, Pavel Trefil^2^, Anna Koslová^1^, Markéta Reinišová^1^, Dana Kučerová^1^, Jitka Mucksová^2^, Jiří Kalina^2^

###### ^1^Institute of Molecular Genetics, Czech Academy of Sciences, Prague, Czech Republic; ^2^BIOPHARM, Research Institute of Biopharmacy and Veterinary Drugs, Jilove u Prahy, Czech Republic

**Correspondence:** Jiří Hejnar

*Retrovirology* 2018, **15(Suppl 1)**:P50

Avian sarcoma and leukosis virus (ASLV) diversified into six phylogenetically relative subgroups (A, B, C, D, E, and J) present as either exogenous or endogenous viruses in domestic chicken. These subgroups are unequivocally classified by the subgroup-specific receptor usage. ALV subgroups enter the cell through Tva, a protein belonging to the family of low-density lipoprotein receptors, Tvb, a tumor necrosis factor receptor-related protein, Tvc, a protein of the butyrophilin family with two immunoglobulin-like domains, or Tvj identified as the chicken Na+/H+ exchanger type 1 (chNHE1) with twelve predicted transmembrane segments and prominent extracellular loop 1. For all ASLV receptors, virus-resistant alleles exist, mostly due to the frame shift mutations or amino-acid substitutions. For example, single W38 deletion or substitution makes teh NHE1 receptor molecule resistant to virus entry. Some of ASLV resistant receptor alleles segregate in domestic chicken and can be used for breeding the ASLV-resistant lines. On the other hand, resistant alleles for NHE1 have not been found in chicken.

In addition, we describe a new technique of transgenesis in chicken, which improves the efficiency of gene modification (including gene introduction, CRISPR/Cas-9-mediated knock-outs and knock-ins) and skips the chimeric G_0_ stage. This technique might become the state-of-art for biotechnological creation of ALV-resistance in the future.

**Keywords:** Avian leukosis virus; Host resistance; Transgenesis in chicken; Receptor

#### P51 Vif-Resistant APOBEC3G-Expressing Lentiviral Vectors for HIV-1 Gene Therapy

##### Krista Delviks-Frankenberry^1^, Daniel Ackerman^1^, Nina Timberlake^2^, Maria Hamscher^1^, Olga Nikolaitchik^3^, Wei-Shau Hu^3^, Bruce Torbett^2^, Vinay K. Pathak^1^

###### ^1^Viral Mutation Section, HIV Dynamics and Replication Program, NCI at Frederick, Maryland, USA; ^2^The Scripps Research Institute, La Jolla, California, USA; ^3^Viral Recombination Section, HIV Dynamics and Replication Program, NCI at Frederick, Maryland, USA

**Correspondence:** Krista Delviks-Frankenberry

*Retrovirology* 2018, **15(Suppl 1)**:P51

Combination antiretroviral therapy (ART) is associated with high cost, drug toxicities, lack of adherence, and drug resistance. Therefore, alternate strategies to suppress HIV-1 replication in the absence of ART are needed to achieve a functional cure. APOBEC3G (A3G) is a host restriction factor that inhibits HIV-1 replication by inducing lethal hypermutation and inhibiting reverse transcription and integration. However, HIV-1 encodes the protein Vif, which induces A3G degradation, allowing successful viral replication. Here, we developed novel self-activating lentiviral vectors to express Vif-resistant A3G mutants that inhibit HIV-1 replication and evaluated their potential as a therapeutic strategy to control HIV-1 replication and viremia.

Standard lentiviral vectors cannot be used for delivery of A3G because their expression in the virus-producing cells inactivates the therapeutic virus. Novel self-activating lentiviral vectors were created that maintain an inactive Vif-resistant A3G mutant (D128K) in virus-producing cells using directly-repeated nucleotide sequences. Upon infection, direct repeats are removed during reverse transcription to express functional A3G-D128K in the target cells. HIV-1 replication kinetics were evaluated in infected T cell lines expressing A3G-D128K and tested for the emergence of resistant virus.

Self-activating vectors allowed for successful virus production and delivery of A3G-D128K to target cells; direct-repeat deletion was 88–98% efficient. CD4+ T cell lines CEM, CEMSS and PM1 expressing A3G-D128K successfully restricted NL4-3 replication. Subtype C and intersubtype recombinant subtype AE also failed to replicate in A3G-D128K expressing cells as well as patient isolates exhibiting higher genetic diversity; however, SIV and HIV-2 could replicate since their Vif proteins could counteract A3G-D128K. No A3G-D128K-resistant NL4-3 virus emerged in CEM/A3G-D128K cells in culture after passaging for 3.5 months, suggesting a high genetic barrier for selection of viral variants that can overcome A3G-D128K restriction. Analysis of proviral DNA showed G-to-A hypermutation patterns in the A3G context, consistent with inhibition by A3G-D128K expression. Infectious titers of > 10E8/ml allowed for efficient delivery of A3G-D128K to CD34+ hematopoietic stem cells without cytotoxicity. These studies establish feasibility of the gene therapy strategy using Vif-resistant A3G-D128K to achieve a functional cure for HIV-1 infection.

**Keywords:** APOBEC3G; Gene Therapy; HIV-1; Functional Cure

#### P52 HIV-1 progeny formation inhibited by modulating the Tsg101:Gag p6 interaction

##### Maike Voges^1,2^, Birgit Schäfer^1^, Alexander Buntru^3^, Ilona Hauber^1,2^, Jan Chemnitz^1,2^, Erich Wanker^3^, Stefan Pöhlmann^4^, Karl-Heinz Wiesmüller^5^, Gerald Bacher^6^ and Joachim Hauber^1,2^

###### ^1^Heinrich Pette Institute – Leibniz Institute for Experimental Virology, Martinistrasse 52, D-20251 Hamburg, Germany; ^2^German Center for Infection Research (DZIF), partner site Hamburg, Germany; ^3^Max Delbrück Center for Molecular Medicine, Robert-Rössle-Straße 10, D-13092 Berlin, Germany; ^4^Infection Biology Unit, German Primate Center, D-37077 Göttingen, Germany; ^5^EMC microcollections GmbH, Sindelfinger Strasse 3, D-72070 Tübingen, Germany; ^6^Novartis Pharma AG, CH-4002, Switzerland

**Correspondence:** Maike Voges

*Retrovirology* 2018, **15(Suppl 1)**:P52

The current combination antiretroviral therapy (cART) significantly prolongs the life expectancy of infected patients. However, effective cART requires lifelong treatment, and unfortunately, may be accompanied by substantial toxicities and/or the occurrence of drug-resistant viruses. Therefore, new antiretroviral strategies are needed to improve and significantly expand current cART options, which act by targeting retroviral enzymes and virus attachment or entry [1, 2]. However, no antiretroviral drugs that specifically interfere with HIV assembly, budding or particle release are currently available [3]. Thus, targeting the virus at these particular steps in its lifecycle may provide novel antivirals with a unique mode of action.

The endosomal sorting complexes required for transport (ESCRT-I, -II, and -III) orchestrate the sorting of membrane proteins into lysosomes via multivesicular bodies (MVB), mediate membrane abscission in cytokinesis, and are involved in virus budding [4, 5]. The complexes mediate human immunodeficiency virus type 1 (HIV-1) particle release by direct binding of the host protein ESCRT-I component called tumor susceptibility gene 101 (Tsg101) to the p6 domain of the retroviral Gag polyprotein. This step is essential in the HIV-1 life cycle.

Here we demonstrate that an experimental drug, the all D-amino acid immunomodulatory peptide RDP58, inhibits the release of R5- and X4-tropic HIV-1, as well as antiretroviral drug-resistant viruses in T cell lines and primary human CD4+ T lymphocytes by promoting binding between host Tsg101 and HIV-1 p6Gag proteins. No measurable drug-induced adverse effects on cell cycle transition, apoptosis, and general cell viability were observed. These findings indicate that components of the ESCRT machinery, such as Tsg101, may serve as promising drug targets for developing novel anti-retroviral therapies.

**Keywords:** cART; ESCRT; HIV therapy; Experimental drug; HIV release

**References**

1. Pau AK, George JM. Antiretroviral therapy: current drugs. Infect Dis Clin North Am 2014;28:371–402.
2. Spearman P. HIV-1 Gag as an Antiviral Target: Development of Assembly and Maturation Inhibitors. Curr Top Med Chem 2016;16:1154–1166.
3. Chen H, Liu X, Li Z, Zhan P, De Clercq E. TSG101: a novel anti-HIV-1 drug target. Curr Med Chem 2010;17:750–758.
4. Slagsvold T, Pattni K, Malerod L, Stenmark H. Endosomal and non-endosomal functions of ESCRT proteins. Trends Cell Biol 2006;16:317–326.
5. Votteler J, Sundquist WI. Virus budding and the ESCRT pathway. Cell Host Microbe 2013;14:232–241.

#### P53 Quantification of HIV-1 Protease Autoprocessing and Its Drug Resistance

##### Liangqun Huang^1^, Linfeng Li^2^, Chihfeng Tien^1^, Daniel V. LaBarbera^2^, Chaoping Chen^1*^

###### ^1^Department of Biochemistry and Molecular Biology, Colorado State University, Fort Collins, Colorado, USA; ^2^High-Throughput Screening and Chemical Biology Core Facility, Skaggs School of Pharmacy and Pharmaceutical Sciences, University of Colorado Anschutz Medical Campus, Aurora, Colorado, USA

**Correspondence:** Chaoping Chen

*Retrovirology* 2018, **15(Suppl 1)**:P53

HIV-1 protease autoprocessing liberates the free mature protease from its Gag-Pol polyprotein precursor through a series of highly regulated autoproteolysis reactions. We here report optimization and evaluation of a cell-based functional assay in 384-well format for autoprocessing quantification using fusion precursors in combination with AlphaLISA (amplified luminescent proximity homogeneous assay ELISA). This AlphaLISA quantification (Fig. 1) and conventional western blotting detection demonstrated reasonable consistency with the AlphaLISA being high throughput screen compatible and using significantly less samples. By screening a collection of 130 known protease inhibitors, the AlphaLISA assay confirmed that all 11 anti-HIV protease inhibitors in the library were able to suppress precursor autoprocessing at micromolar concentrations. Meanwhile, the other protease inhibitors had no impact on precursor autoprocessing. Several pilot screens against a total of ~ 23,000 compounds demonstrated satisfactory performances with z’ factors > 0.45 and S/N ratios > 15 on average. About 150 positive hits were cherry-picked and subsequently determined to be false positive upon retesting with the same assay. These results illustrated a high selectivity of this assay with an approximate 0.1% false positive rate. Furthermore, AlphaLISA quantification of fusion precursors carrying mutations known to cause resistance to protease inhibitors (PIs) faithfully recapitulated the reported resistance, suggesting that precursor autoprocessing is a critical step attributed to PI resistance. Taking together, this reported AlphaLISA assay would be a useful tool suitable for identification and characterization of HIV-1 protease autoprocessing specific inhibitors.

**Fig. 1:**
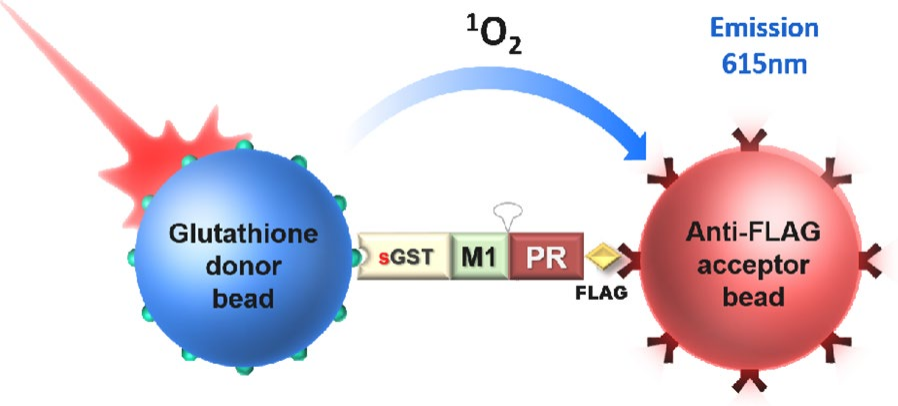
AlphaLISA detection of the fusion precursor

**Keywords:** AlphaLISA; Autoprocessing; High-throughput screen; HIV-1; Protease; Protease inhibitor; Resistance

### TOPIC 8: HIV cure minisymposium

#### P54 A novel sensitive indicator cell line to quantify the reactivatable latent viral load

##### David W. Gludish^1^, Fanny Salasc^2^, Hoi Ping Mok^2^, Isobel Jarvis^2^, Andrew M.L. Lever^2^, David G. Russell^1^

###### ^1^Cornell University College of Veterinary Medicine, New York, USA; ^2^Department of Medicine, University of Cambridge, UK

**Correspondence:** Fanny Salasc

*Retrovirology* 2018, **15(Suppl 1)**:P54

The quantitative virus outgrowth assay measures the size of the HIV latent reservoir. The assay utilises cells originating from a rigorously defined latent compartment and only includes replication competent latent viruses in its measurement. It is thus a definitive minimal estimate of the reservoir size and remains the gold standard assay in HIV latency research. However the conventional assay is laborious, limiting its utility.

We have previously reported our modification of the virus outgrowth assay, using SupT1-CCR5 instead of allogeneic PBMC to support the replication of reactivated latent viruses rendering the assay more scalable. Indeed, the use of SupT1-CCR5 cell line also significantly improves the reproducibility of the assay. Here we explored the use of a sensitive indicator cell line to further improve the assay. A construct that expresses both Gaussia luciferase and GFP in a Tat and Rev dependent manner was engineered into SupT1-CCR5. This results in a novel indicator cell line, SupT1-GGR5 that supports the replication of both X4 and R5-tropic HIV and expresses Gaussia luciferase and GFP upon HIV infection. Using Gaussia luciferase or GFP expression as readouts instead of conventional supernatant p24 ELISA significantly reduces the labour required for the virus outgrowth assay. Gaussia luciferase is particularly valuable, as it can be sampled from the supernatant without sacrificing the culture. To assess the utility of this cell line in the virus outgrowth assay, we compared the overall sensitivity and the kinetics of virus outgrowth assays using SupT1-GGR5 with that using SupT1-CCR5. The results will be presented in the meeting.

**Keywords:** HIV; Latency; Viral outgrowth assay; Indicator cells

#### P55 Beneficial impact of early treatment on restriction factor expression profile

##### Clarissa Van Hecke^1^, Wim Trypsteen^1^, Eva Malatinkova^1^, Ward De Spiegelaere^2^, Karen Vervisch^1^, Chris Verhofstede^1^, Margaret Johnson^2^, Sabine Kinloch-de Loes^2^, Magdalena Sips^1^, Linos Vandekerckhove^1^

###### ^1^HIV Cure Research Center, Ghent University, Corneel Heymanslaan 10, Gent, Belgium; ^2^Department of Morphology, Faculty of Veterinary Medicine, Ghent University, Salisburylaan 133, Merelbeke, Belgium; ^3^Royal Free Hospital, Pond St, Hampstead, London, United Kingdom

**Correspondence:** Clarissa Van Hecke

*Retrovirology* 2018, **15(Suppl 1)**:P55

Host restriction factors become upregulated early in HIV infection as part of the innate immune response to suppress viral infectivity, and activity of some of them, e.g. SLFN11 has been linked to non-progressive phenotype of HIV infection. Early treated cohorts comprising of patients treated during acute seroconversion are considered a promising group to reach functional cure by acquisition of non-progressive phenotype. We evaluated HIV host restriction factors, cofactors and interferon-stimulated genes (ISGs) in early and late treated cohorts and compared their profile with progressive and non-progressive HIV infection to further characterize their role in controlling infection.

The expression profile of seven HIV restriction factors, two cofactors and two ISGs (APOBEC3G, SAMHD1, BST2 (encoding TETHERIN), TRIM5, MX2, SLFN11, PAF1, PSIP1 (encoding LEDGF/p75), NLRX1, MX1 and IFIT1) was evaluated by qPCR in 104 HIV infected patients: patients treated during seroconversion (Early treated) or chronic infection (Late treated), recent ART-naïve seroconverters, ART-naïve progressors, ART-naïve non-progressors and non-infected controls. Patients were recruited in Royal Free Hospital London and Ghent University Hospital. Principal Component Analysis (PCA) and Kruskal–Wallis (KW) statistical analysis were performed.

Both, univariate and PCA analysis demonstrated completely distinctive expression pattern of restriction factors in early- and late-treated cohorts. Restriction factor levels of early treated HIV patients were significantly upregulated in comparison to late treated patients (APOBEC3G: p < 0.01; SAMHD1: p < 0.001; SLFN11: p < 0.001; BST2: p < 0.001). Further analysis demonstrated similarities between early treated patients and ART-naïve non-progressors, such as upregulation of SLFN11 and BST2.

In conclusion, early treatment potentially prevents depletion of innate antiviral responses in comparison to late treated subjects. Elevated expression of SLFN11 and BST2 in ART-naïve non-progressors and early treated subjects implies that these restriction factors actively contribute to the non-progressive phenotype in these cohorts.

**Keywords:** HIV; Restriction factors; Cohorts; Early treatment

#### P56 Heme-arginate as a latency reversing agent for HIV-1 cure

##### Madlenakova Michaela^1,2^, Shankaran Prakash^1^, Hajkova Vera^1,2^, Jilich David^3,^, Machala Ladislav^3^, Martasek Pavel^2^, Melkova Zora^1,2^

###### ^1^Department of Immunology and Microbiology, Charles University, 1^st^ Faculty of Medicine, Studnickova 7, 12800 Prague, Czech Republic; ^2^BIOCEV, Biotechnology and Biomedicine Center of the Academy of Sciences and Charles University, Prumyslova 595, 252 50 Vestec, Czech Republic; ^3^AIDS Center, Na bulovce Hospital, Budínova 67/2, 180 81 Prague, Czech Republic

**Correspondence:** Madlenakova Michaela

*Retrovirology* 2018, **15(Suppl 1)**:P56

Current antiretroviral therapy can suppress HIV-1 infection to undetectable levels of plasma viremia, but integrated HIV-1 genomes persist in highly stable reservoir of latently infected cells. This latent HIV-1 reservoir is a major barrier to HIV-1 cure. Presently, there is a substantial ongoing effort to identify therapeutic approaches that would eliminate or reduce the size of the latent HIV-1 reservoir. Current strategies towards HIV-1 cure involve namely attempts to reactivate and purge HIV-1 latently infected cells.

Normosang (Heme-arginate, HA) was approved for treatment of acute hepatic porphyria. In our laboratory, we have demonstrated stimulatory effect of HA on reactivation of latent HIV-1 in cell lines and also in PBMCs of HIV+ patients ex vivo. To confirm original hypothesis that HA could be used as a latency reversing agent (LRA) in human, we have administered Normosang to HIV+ subjects on cART including integrase inhibitor. Here we describe in vivo effects of Normosang on reactivation of the latent HIV-1 at specific time-points after its administration characterized using quantitation of cell-associated HIV-1 RNA in PBMCs of HIV+ subjects by means of 2-step semi-nested RT-qPCR.

Current strategies involving latent HIV-1 reactivation do not seem to affect the size of the latent reservoir, thus it is important to search for new approaches towards LRAs. The stimulatory effects of Normosang involve a heme/iron-mediated Fenton reaction resulting in the increased redox stress, thus leading to latent HIV-1 reactivation. In summary, our work defines a new redox-based approach towards HIV-1 latency reversal in vivo and could provide a basis for new therapeutic approaches towards HIV-1 cure.

**Keywords:** Reactivation; Heme-arginate; Normosang; Latency reversal

#### P57 Novel ImmTAV™ molecules for the treatment of HIV

##### Jacqui Brener^1^, Bea Choi^1^, Mary Connolly^1^, Shelley Cook^1^, Andrew Knox^1^, Joshua Long^1^, Ruth Martinez Hague^1^, Sam Paston^1^, Praveen Singh^1^, Andrew Walker^1^, Katrin Wiederhold^1^, Hongbing Yang^2^, Lucy Dorrell^1,2^, Kevin Pojasek^1^, Namir Hassan^1^, Bent Jakobsen^1^

###### ^1^Immunocore Ltd., 101 Park Drive, Milton Park, Abingdon, OX14 4RY, UK; ^2^Nuffield Department of Medicine, University of Oxford, NDM Research Building, Roosevelt Drive, Oxford OX3 7FZ

**Correspondence:** Jacqui Brener

*Retrovirology* 2018, **15(Suppl 1)**:P57

Immunotherapeutic strategies harness the body’s own immune system to eradicate cancer cells and pathogens, such as bacteria or viruses. T cells play a critical role in directing potent and antigen-specific immune responses. The T cell receptor (TCR) enables T cell antigen recognition by interacting with short peptides derived from intracellularly processed proteins presented on the cell surface by human leukocyte antigens (HLA). As the majority of the proteome, as well as foreign proteins, are processed and presented by HLA molecules on the surface of target cells, and hence can be targeted by a TCR, TCR based therapies represent a distinct advantage over antibody-based therapies, that are limited to targeting only secreted or cell surface proteins.

A limitation of natural antigen-specific TCRs is their low to moderate (uM to nM range) binding affinities that may not effectively bind target specific antigens. With this limitation in mind, at Immunocore, we have developed Immune mobilising monoclonal TCRs Against Cancer (ImmTAC™) with affinity-enhanced (pM affinity) monoclonal T cell receptors fused to anti-CD3 scFv, which have the capability to redirect and activate effector T cells, hence enhancing the T cell response.

Building on the ImmTAC platform, here we describe how we are applying our TCR technology to address unmet needs in infectious diseases, including HIV, with the development of Immune mobilising monoclonal TCRs against viruses (ImmTAV). While anti-retroviral therapy has proved successful in suppressing disease burden in HIV-infected individuals, the immune system is unable to eradicate the latent reservoir. ImmTAV technology has the potential to re-direct non-specific T cells to eliminate latently infected cells directly or following treatment with latency reversing agents.

Our platform involves isolating T cell clones that specifically recognise viral antigens. To confirm antigen binding identified TCRs are expressed in *E. coli*, refolded in vitro, and their binding affinities to the target pHLA tested by surface plasmon resonance (SPR). Specific TCRs are affinity matured through directed evolution using phage display, and ImmTAV molecules are generated through combining mutations and coupling of an anti-CD3 scFv effector function. ImmTAV molecules are tested in ex vivo cellular assays that model the mode of action in vivo.

HIV-specific ImmTAV molecules aim to reduce cellular reservoirs and thus provide a functional cure.

**Keywords:** Immunotherapeutic; Bi-specific; HIV treatment; HIV functional cure

#### P58 Contribution of lncRNAs in the establishment of HIV latency in CD4+ T cell models

##### Wim Trypsteen^1^, Clarissa Van Hecke^1^, Tinus Schynkel^1^, Cory White^2^, Christopher Woelk^2^, Alberto Bosque^3^, Celsa Spina^4^, Steve Lefever^5^, Pieter Mestdagh^5^, Linos Vandekerckhove^1^*, Nadejda Beliakova-Bethell^4^*

###### ^1^HIV Cure Research Center, Ghent University, Belgium; ^2^Merck Cambridge Exploratory Science Center, Southampton, UK; ^3^Microbiology, Immunology & Tropical Medicine, G. Washington University, USA; ^4^VA San Diego Healthcare System, University of California, San Diego, USA; ^5^Center Medical Genetics, Ghent University, Belgium

*Retrovirology* 2018, **15(Suppl 1)**:P58

*Contributed equally

HIV cure research has been hampered by the existence of a latent viral reservoir that persists in infected individuals receiving antiretroviral therapy. To date, most of the cure research has focused on protein-coding genes but recently the interest in the study of long non-coding RNA (lncRNA) has risen, as these molecules could provide insight in new therapeutic strategies and further complete insight in the HIV life cycle.

Transcriptome profiling was performed (total RNA-Seq) in two primary HIV latency models of CD4 T cells to investigate changes in lncRNA expression, Spina and Planelles model. Subsequently, differentially expressed mRNAs and lncRNAs were identified in both models and a guilt-by-association analysis was implemented to infer biological roles for the lncRNAs in HIV latency.

In the primary HIV latency models, we respectively identified 826 & 471 mRNAs (87.8% & 76.2%) and 115 & 147 lncRNAs (12.2% & 23.8%) that were significantly differentially expressed (FDR < 0.05) between uninfected and latently infected CD4+ T cells. Between models, 10 lncRNAs were overlapping (e.g. NEAT1 and PVT1) and many of these lncRNAs were associated with pathways involved in cell cycle regulation and pathways with a link to HIV latency: mTOR, IL-7, PTEN and CCR5. In addition, a cluster of 17 lncRNAs was associated with the p53 pathway and corroborate earlier findings in the Planelles model that illustrated p53-dependent latency establishment. One of these upregulated p53-linked lncRNAs, 7SLRNA, has a characterized inhibitory role in the p53 pathway and would suit as a possible new therapeutical target.

Altogether, this study demonstrates that several lncRNAs play a role in HIV latency and can be linked to biological pathways with importance in HIV latency establishment and maintenance. Some of these lncRNAs, i.e. NEAT1, PVT1 or 7SLRNA, represent possible targets for reversing HIV latency and contribute to a HIV cure.

**Keywords:** HIV-1 Latency; Long non-coding RNAs; Transcriptome; HIV-1 cure

#### P59 HIV-1 proviral DNA Methylation profiles show differential methylation between patient groups

##### Sam Kint^1,2^, Wim Van Criekinge^1^, Linos Vandekerckhove^2^

###### ^1^Biobix, Ghent University, Coupure Links 653, 9000 Ghent, Belgium; ^2^HCRC, Ghent University, C. Heymanslaan 10, 9000 Ghent, Belgium

**Correspondence:** Sam Kint

*Retrovirology* 2018, **15(Suppl 1)**:P59

The last hurdle to an HIV cure is to understand and interact with HIV latency. This latent reservoir is responsible for viral rebound following treatment interruption, and by interacting with its mechanisms, latency can be reversed (shock and kill) or consolidated (block and lock). Epigenetic modifications such as DNA methylation and histone acetylation in the integrated proviral genome are at least partially responsible for HIV latency initiation and maintenance. One of the most important and most studied epigenetic modifications is DNA methylation. This modification is in general associated with silencing of affected genes. This silencing regulation in HIV latency is clearly shown in different in vitro studies, but due to low proviral DNA abundancy and high genomic heterogeneity, obtaining reliable and reproducible patient-derived data is hampered, resulting in different contradictory publications. To reliably measure the DNA methylation in the HIV provirus, we developed a bisulfite-based next generation sequencing assay to measure the methylation state of four out of five CpG Islands found in the proviral HIV genome. We compared methylation in PBMC samples in four different patient cohorts: early treated seroconverters (ET), late treated patients (LT), ART- naïve seroconverters (SRCV) and long-term non-progressors (LTNP). The findings of this study are that (i) CpG-Islands of the promoter region have low overall methylation percentages, and (ii) that they are slightly less methylated in ET and LTNP (differential methylation (DM) < 5%). (iii) The CpG-Islands in the ENV region show no DM between ET and LT patients, but a decrease of 33.83% in SCRV, and an increase of 9.69% in LTNP compared to ET/LT.

These results show that DNA methylation in the promoter region is an early process, resulting in an early latent reservoir formation, while ENV methylation is a later process, potentially involved in latency maintenance.

**Keywords:** DNA Methylation; Epigenetics; HIV Latency; Bisulfite sequencing

